# FDXR upregulation by p63/p73 is a prognostic and therapeutic marker of adult T-cell leukemia/lymphoma

**DOI:** 10.1038/s44321-026-00535-0

**Published:** 2026-10-12

**Authors:** Lara Gallucci, Maria Josephine, Yasunori Kogure, Alex Ap Rosini Silva, Vincent Cantaloube-Ferrieu, Marcos Eduardo Souza Abreu, Trusha Ramteke, Phoebe Kibble, Guilherme Lucchesi D Angelo Braz, Rubens de Assis Santos Sebastião, Luiza Amoroso Lambaz, Aijun Lin, Beth Hoare, Felipe Custódio Carvalho dos Santos, Sophie Shackley, Alex Quin, Sofia Luari Moelas Giuzio, Philip A Lewis, Wujuan Zhang, Youko Nukui, Juliana Pereira, Hebert Fabricio Culler, Takuro Kameda, Kazuya Shimoda, Kate Heesom, Aaron R Goldman, Ester Cerdeira Sabino, Seishi Ogawa, Fabio Eudes Leal, Mattia Forcato, Keisuke Kataoka, Sheila de Oliveira Garcia Mateos, Iart Luca Shytaj

**Affiliations:** 1 School of Biochemistry and Biomedical Sciences, University of Bristol, Bristol, United Kingdom; 2 Division of Molecular Oncology, National Cancer Center Research Institute, Tokyo, Japan; 3 Faculdade de Medicina da Universidade Municipal de São Caetano do Sul, São Caetano do Sul, Brazil; 4 The Wistar Institute, Philadelphia, PA USA; 5 Faculdade de Medicina da Universidade de São Paulo, São Paulo, Brazil; 6 Division of Immunology, Department of Infectious Diseases, Faculty of Medicine, University of Miyazaki, Miyazaki, Japan; 7 Department of Pathology and Tumor Biology, Graduate School of Medicine, Kyoto University, Kyoto, Japan; 8 Institute for the Advanced Study of Human Biology (WPI-ASHBi), Kyoto University, Kyoto, Japan; 9 Kindai University Faculty of Medicine, Osakasayama, Japan; 10 Brazilian National Cancer Institute, Rio de Janeiro, Brazil; 11 Department of Molecular Medicine, University of Padova, Padova, 35122 Italy; 12 Division of Hematology, Department of Medicine, Keio University School of Medicine, Tokyo, Japan

**Keywords:** Cancer, Haematology, Microbiology, Virology & Host Pathogen Interaction

## Abstract

Infection with HTLV-1 can lead to severe diseases, including adult T-cell leukemia/lymphoma (ATL), for which current therapies are inadequate. Selective elimination of chronically infected cells requires identifying cellular markers dysregulated by HTLV-1 that can be exploited therapeutically. Combining metabolomics and proteomics analyses, we identified increased expression of the mitochondrial enzyme ferredoxin reductase (FDXR) as a hallmark of HTLV-1-infected cells. In patient cells, FDXR upregulation mirrored disease severity, was enriched in proliferating tumour cells and predicted worse ATL prognosis. Mechanistically, FDXR upregulation in ATL cells is driven by the transactivating isoforms of two p53 family members, TAp63 and TAp73, and TAp63 expression is, in turn, induced by the IRF4/BATF3 transcriptional axis. In silico drug susceptibility predictions, followed by targeted cytotoxicity, proliferation and Drug-Seq assays demonstrated that FDXR upregulation is associated with increased sensitivity of ATL cells to repurposed chemotherapeutic agents, particularly fluorouracil. Overall, our findings establish FDXR as a novel biomarker of HTLV-1-associated disease progression and ATL prognosis, elucidate its upstream molecular regulation, and highlight its predictive potential for chemotherapeutic sensitivity of ATL cells.

## Synopsis

Human T-cell lymphotropic virus 1 can cause adult T-cell leukemia/lymphoma (ATL), which is prone to chemoresistance and relapse. FDXR is identified as a biomarker of ATL progression and chemosensitivity.

- FDXR upregulation in patient-derived cells mirrors the severity of HTLV-1-associated disease.
- Elevated FDXR levels are linked to worse ATL prognosis.
- FDXR upregulation in HTLV-1+/ATL cells is driven by a pathway converging on two p53 family members, p63 and p73.
- Higher basal or drug-induced FDXR expression is associated with increased sensitivity of ATL cells to fluorouracil and bleomycin.

## Abstract (web summary)

Human T-cell lymphotropic virus 1 can cause adult T-cell leukemia/lymphoma (ATL), which is prone to chemoresistance and relapse. FDXR is identified as a biomarker of ATL progression and chemosensitivity.

## Graphical abstract

**Figure:**
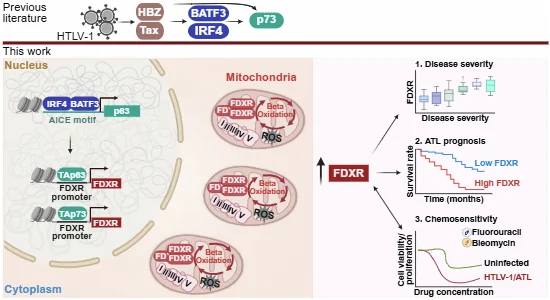

The paper explained **Problem**

Human T-lymphotropic virus type 1 causes chronic infection of multiple immune cell subpopulations. While the infection can remain asymptomatic, it is associated with an estimated lifetime risk of up to 10% for severe HTLV-1-associated disease, including a type of blood cancer called adult T-cell leukemia/lymphoma (ATL). Treatment options for HTLV-1-associated diseases remain limited, with ATL displaying high chemoresistance and frequent relapse, particularly in its most aggressive forms. Biomarkers capable of predicting disease outcomes and treatment response are needed to improve therapeutic selection and patient stratification.

**Results**

Through analyses of multi-omics data of HTLV-1-infected cell models and patient-derived cells, we identified ferredoxin reductase (FDXR) as a marker upregulated with disease severity and associated with poorer ATL prognosis. FDXR had previously been linked to enhanced proliferation and increased sensitivity to chemotherapeutic agents acting through the p53 pathway in solid tumors. Combining in silico transcription factor activity analysis, ChIP-seq data mining, ChIP–qPCR, biochemical assays, and CRISPR-Cas experiments, we reconstructed the regulatory pathway leading to FDXR upregulation. These analyses revealed that FDXR is induced in ATL cells by the transactivating isoforms of two p53 family members, TAp63 and TAp73, and that p63 expression is itself dependent on the IRF4-BATF3 axis, a key regulatory circuit known to support the survival of HTLV-1-infected and ATL cells. We also found that FDXR levels significantly correlated with in vitro susceptibility to repurposed chemotherapeutic agents, particularly fluorouracil and bleomycin, which have not previously been used against ATL. These drugs induced activation of the p53 pathway in ATL patient-derived cells and reduced their proliferation, supporting the potential therapeutic relevance of FDXR as both a biomarker of disease progression and therapeutic vulnerability in ATL.

**Impact**

Our paper extends the relevance of FDXR beyond solid tumors, highlighting it as a novel prognostic biomarker for ATL. Moreover, our results suggest that repurposed chemotherapeutic drugs that activate the p53 family may exploit basal and treatment-induced FDXR expression, potentially providing new therapeutic options to reduce the high chemoresistance and relapse rates typical of ATL.

## Introduction

Adult T-Cell leukemia/lymphoma (ATL) is a hematological malignancy caused by infection with the human T-lymphotropic virus 1 (HTLV-1) (Bangham, 2023). Although ATL is relatively uncommon, occurring in up to 10% of infected individuals after decades of latency, the disease progresses aggressively, particularly in the acute and lymphomatous subtypes, which are characterized by a median survival of less than one year (Valcarcel et al, 2025). HTLV-1 infection can also cause other severe conditions, such as HTLV-1-associated myelopathy/tropical spastic paraparesis (HAM/TSP), a chronic and progressive neurodegenerative disorder (Bangham et al, 2015). Therapeutic options for ATL and HAM/TSP remain inadequate, with the former displaying high relapse rates following chemotherapy, immunotherapy, and even allogeneic stem cell transplant, which is currently the only potentially curative intervention (Cook and Phillips, 2021).

Intensive efforts have focused on the identification of molecular biomarkers enabling risk stratification and targeted therapy. These include viral parameters [e.g., HTLV-1 proviral load (Iwanaga et al, 2010) and clonality (Saito et al, 2025)], soluble immune mediators (Sato et al, 2013), patterns of somatic alterations (Kataoka et al, 2015b) and/or dysregulated expression of host genes (Manivannan et al, 2016; Polakowski et al, 2023; Sugata et al, 2016) and microRNAs (Yamagishi et al, 2012b). Among these, host genes and pathways which are selectively upregulated in infected cells offer opportunities for targeted therapeutic treatment. A proof of concept is provided by CCR4, which is upregulated in the majority of HTLV-1-infected cells (Sugata et al, 2016). The monoclonal antibody mogamulizumab, which selectively targets CCR4, has shown remarkable effects in decreasing proviral load and has displayed anti-tumor activity in clinical trials (Cordover et al, 2024; Ishida et al, 2012; Sato et al, 2024; Yonekura et al, 2020). Nevertheless, overall survival in ATL remains poor, even among responders (Cordover et al, 2024; Yonekura et al, 2020), and no significant clinical benefit could be detected in a recent phase III clinical study of HAM/TSP patients (Sato et al, 2024).

Multi-omics integration has become an increasingly powerful strategy for robust biomarker discovery. Studies combining whole-genome sequencing, epigenomic, and transcriptomic data and, more recently, plasma metabolomics, have enabled the identification and validation of key pathways dysregulated in ATL (Kataoka et al, 2015b; Myers et al, 2024; Tanaka et al, 2025; Toyoda et al, 2023; Van Weyenbergh et al, 2025). On the other hand, proteomic datasets remain underexplored in the field, and the intracellular metabolome of HTLV-1-infected or ATL cells has not been characterized. In this regard, our work on cells infected with the human immunodeficiency virus-1 (HIV-1), which, like HTLV-1, is a retrovirus with preferential tropism for activated CD4 T cells, shows that incorporating intracellular metabolomics into multi-omics pipelines can uncover metabolic vulnerabilities amenable to therapeutic targeting (Shytaj et al, 2021).

In the present study, we integrate multiple layers of bulk and single-cell omics data from cell models and patient-derived samples and demonstrate that HTLV-1-infected cells upregulate the mitochondrial enzyme ferredoxin reductase (FDXR) as part of a broader metabolic reprogramming toward enhanced mitochondrial activity. *FDXR* mRNA expression correlates with disease progression and poor prognosis in ATL, and is more frequent, at a single cell level, in proliferating tumor cells. Mechanistically, FDXR induction is driven by the infected-cell-specific expression of p53 family members p63 and p73, specifically their transactivating isoforms (TAp63 and TAp73). The ectopic expression of TAp63 in ATL cells is, in turn, regulated by the IRF4/BATF3 transcriptional axis, ultimately linking FDXR upregulation with a key driver (Nakagawa et al, 2018c) of HTLV-1-associated disease. By combining gene expression profiling with drug-susceptibility screening, we identify FDXR expression levels as a predictor of sensitivity to several chemotherapeutic agents not previously tested in ATL and include validation of fluorouracil and bleomycin responses in vitro and ex vivo.

Together, our results uncover FDXR upregulation as a mitochondrial hallmark of HTLV-1 infection and pathogenesis that may serve as a prognostic tool and therapeutic vulnerability to selectively target ATL cells.

## Results

### Mitochondrial FDXR upregulation correlates with HTLV-1-associated disease severity and ATL mortality

**Figure EV1:**
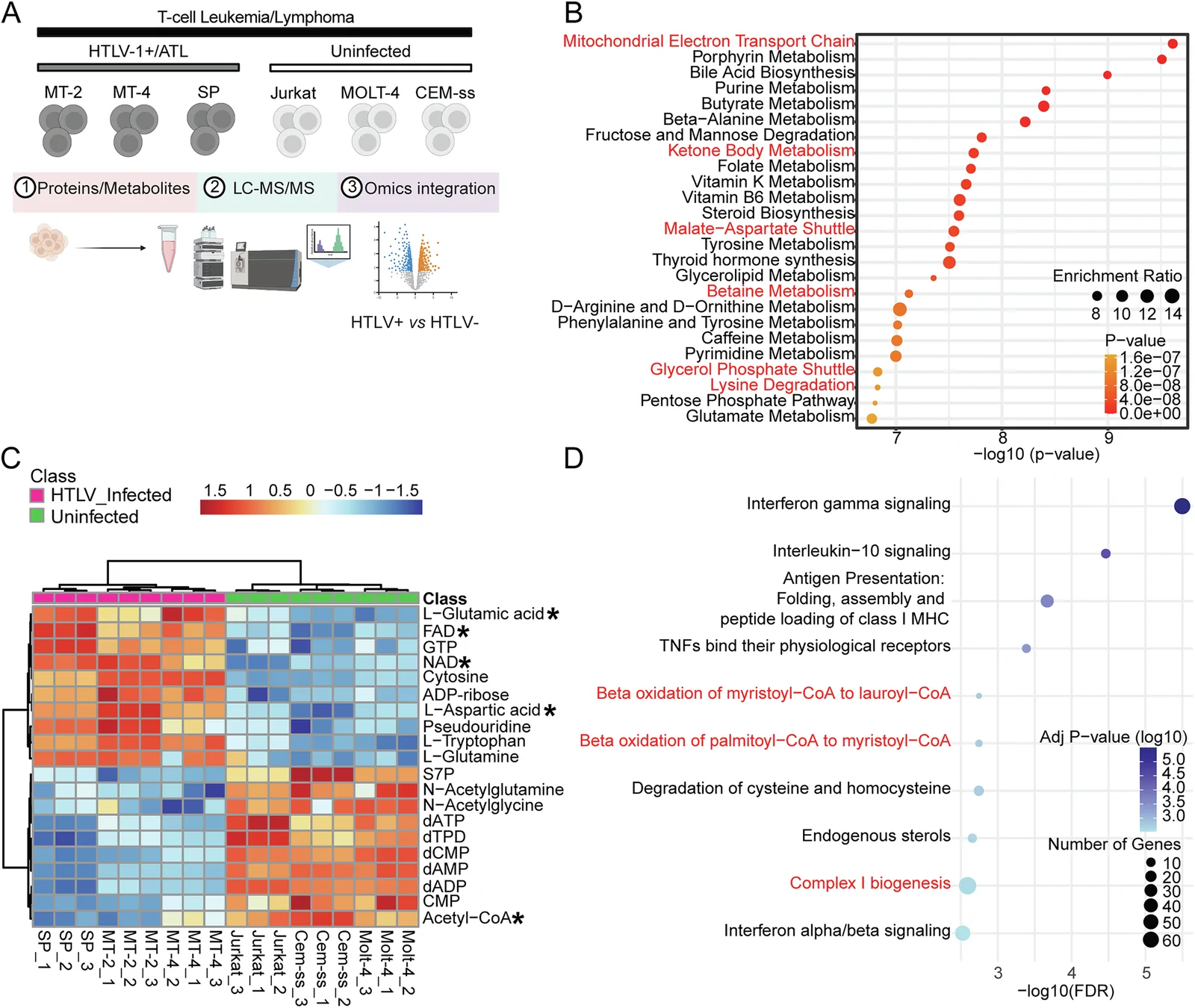
Mitochondrial metabolic alterations in HTLV-1^+^/ATL cell lines. (**A**) Experimental design. The metabolome and proteome of three HTLV-1^+^/ATL cell lines (MT-2, MT-4, SP) were compared with three uninfected lymphoid cell lines (Jurkat, MOLT-4, and CEM-ss). Cell lysates were analysed in triplicate for metabolomics and in quadruplicate for proteomics. (**B**) Pathway enrichment analysis of metabolomic profiles comparing HTLV-1^+^ and uninfected cells. The analysis was performed on SMPDB metabolite sets using the GlobalTest score test implemented in MetaboAnalyst. (**C**) Heatmap of the twenty most significantly altered metabolites in HTLV-1^+^ versus uninfected cells, ranked by *t*-test followed by Benjamini–Hochberg FDR correction. (**D**) Proteomic pathway analysis showing the ten most upregulated pathways in HTLV-1–infected cells. The analysis was performed on Reactome gene sets using a two-sided CAMERA gene set test followed by Benjamini–Hochberg FDR correction. Pathways that are exclusively or predominantly mitochondrial in panels (**B**, **D**) are highlighted in red, and metabolites from these pathways that appear in the heatmap (**C**) are indicated by asterisks.

To identify cellular markers of metabolic dysregulation associated with HTLV-1 infection, we initially conducted a parallel metabolomic and proteomic analysis using lymphoid cell models (Fig. EV1A). We used two HTLV-1-infected cell lines (MT-2 and MT-4), one ATL-derived cell line (SP), and compared them to three uninfected controls (Jurkat, MOLT-4, and CEM-ss). Metabolomic profiling identified multiple mitochondrial pathways as significantly enriched in infected cells, with the electron transport chain, part of oxidative phosphorylation (OXPHOS), emerging as the most significantly enriched pathway in the overall analysis (Fig. EV1B). Metabolites represented in the enriched mitochondrial pathways were among the most significantly altered and were predominantly upregulated in infected cells, with the exception of acetyl-CoA (Fig. EV1C). Consistent with these findings, proteomic analysis revealed significant upregulation of pathways involved in fatty acid β-oxidation and mitochondrial Complex I biogenesis (within the OXPHOS system) in HTLV-1-infected cells (Fig. EV1D; Dataset EV1).

**Figure 1:**
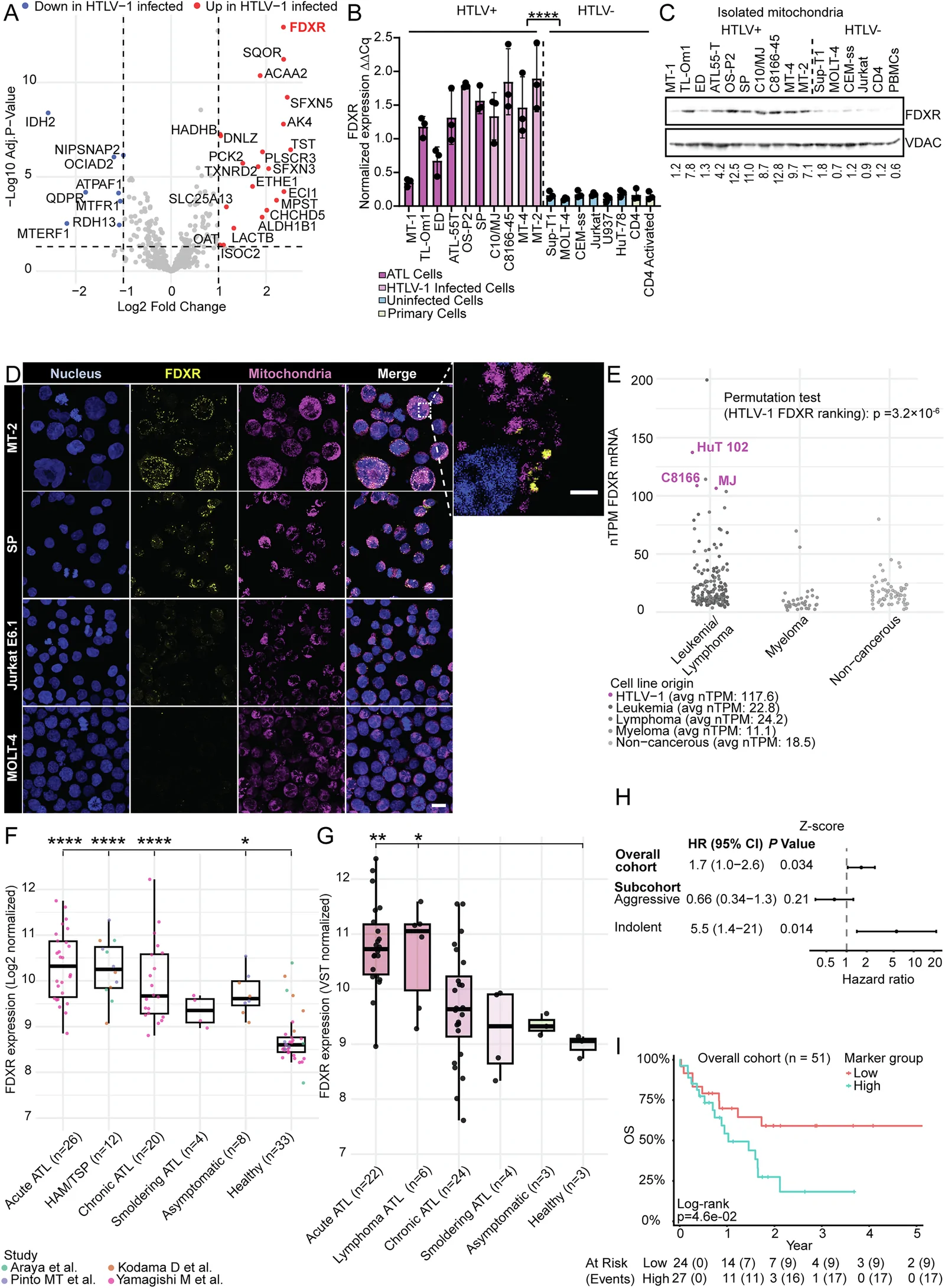
FDXR upregulation in HTLV-1^+^/ATL cells predicts disease severity and ATL mortality. (**A**) Three HTLV-1^+^/ATL (MT-2, MT-4, and SP) and three uninfected (Jurkat, MOLT-4, and CEM-ss) lymphoid cell lines were lysed in quadruplicate and subjected to proteomic analysis. The volcano plot shows differentially expressed mitochondrial proteins. Mitochondrial proteins were filtered using the Mitocarta database v3.0. Differential expression was assessed using a two-sided empirical Bayes-moderated *t*-test followed by Benjamini–Hochberg FDR correction (limma package). (**B**–**D**) FDXR expression in HTLV-1^+^, ATL, and uninfected cell lines and primary cells as measured by qPCR (**B**), western blot of isolated mitochondria (**C**), and immunofluorescence (**D**). In panel (**B**), each dot represents an independent qPCR assay (*n* = 3), and data were presented as mean ± SD. Relative FDXR mRNA levels were calculated by ΔΔCq using TBP expression as a reference. Data were analyzed by the nonparametric Wilcoxon rank-sum test (*p* = 4.6e-05). Panel (**C**) shows a representative blot, with relative protein levels reported below each lane and quantified using VDAC as the loading control. Panel (**D**) shows representative z-projection images of nuclei (DAPI), FDXR (yellow), and mitochondria stained with MitoTracker (magenta). The boxed region displays a single optical section highlighting mitochondrial localization of FDXR. Scale bars: 10 μm (main) and 2 μm (inset). (**E**) FDXR mRNA expression (nTPM) across 259 blood cancer and non-cancerous cell lines, with HTLV-1⁺ cell lines highlighted in magenta. Median expression values by cell origin are shown below. The significance of the enrichment of HTLV-1 cell lines among the top cell models for FDXR expression in the dataset was assessed by permutation testing (*p* = 3.2e-06). RNA-Seq data for all cell lines were retrieved from the Protein Atlas. (**F**) Meta-analysis of microarray datasets of CD4 T cells isolated from PLHTLV stratified by disease condition and healthy controls (Data ref: Araya et al, 2025; Data ref: Kodama et al, 2020; Data ref: Pinto et al, 2014; Data ref: Yamagishi et al, 2012a). Data were background-corrected, normalized, and batch-adjusted using the limma (Ritchie et al, 2015) and sva (Leek et al, 2012) (ComBat) packages. Each dot depicts log2-normalized FDXR expression levels from a single donor, while box plots show median ± IQR for each subgroup. Differences in FDXR expression were tested using a linear mixed-effects model, with disease status as a fixed effect and study as a random intercept. Post hoc contrasts comparing each disease group with uninfected controls were performed using study-adjusted estimated marginal means (using the emmeans package), followed by Holm correction for multiple testing (Acute ATL *p* = 4.8e-11; HAM/TSP *p* = 6.4e-5; Chronic ATL *p* = 2.3e-07; Smoldering ATL *p* = 0.07; Asymptomatic *p* = 0.03). (**G**) RNA-Seq data (Data ref: Kataoka et al, 2015a) from tumor and CD4 T cells isolated from PLHTLV stratified by disease condition and healthy controls. Each dot depicts variance-stabilized (VST) FDXR expression levels from a single donor, while box plots show median ± IQR for each subgroup. Differential expression between each subgroup and healthy controls was tested using DESeq2, with *p* values adjusted for multiple testing by Benjamini–Hochberg FDR correction. (Acute ATL *p* = 0.001; Lymphoma ATL *p* = 0.01; Chronic ATL *p* = 0.15; Smoldering ATL *p* = 0.77; Asymptomatic *p* = 0.90). (**H**, **I**) Association of FDXR expression with overall survival in ATL patients. (**H**) Cox proportional-hazards analysis of continuous Z-score–normalized FDXR expression levels in the full cohort (*n* = 51) and in aggressive (acute, lymphoma; *n* = 27) and indolent (chronic, smoldering; *n* = 24) ATL subtypes. Error bars indicate 95% CI. (**I**) Kaplan–Meier analysis of overall survival in patients stratified into high and low FDXR expression groups based on the median Z-score. FDXR expression values were derived from RNA-Seq (Data ref: Kataoka et al, 2015a) and analysed in ATL patients with available survival data [*n* = 51 (Kataoka et al, 2015b; Kogure et al, 2022)]. Overall survival in (**H**, **I**) was censored at hematopoietic stem cell transplantation, and survival differences in (**I**) were assessed using the log-rank test. ****p < 0.0001; **p < 0.01; *p < 0.05. Source data are available online for this figure.

Differential expression analysis of proteomic data, performed by filtering the results to proteins with known mitochondrial localization using the MitoCarta repository (Rath et al, 2021), uncovered ferredoxin reductase (FDXR) as the most significantly upregulated mitochondrial protein in infected cells (Fig. 1A; Dataset EV2). Functionally, FDXR contributes to lipid homeostasis, iron-sulfur cluster biogenesis, and redox regulation, and depends on flavin adenine dinucleotide (FAD) as a cofactor (Slone et al, 2020; Zhang et al, 2022), which our metabolomic analysis revealed as significantly increased in infected cells (Fig. EV1C). We thus tested FDXR expression by qPCR, western blotting of isolated mitochondria, and immunofluorescence using a panel of sixteen cell lines as well as activated and resting primary CD4 T cells and PBMC. The results confirmed a significant upregulation of FDXR in HTLV-1-infected and ATL cell lines (Fig. 1B,C) as well as its mitochondrial localization (Fig. 1C,D). Two ATL models, MT-1 and ED cells, exhibited elevated FDXR mRNA, but not protein levels (Fig. 1B,C). The increase in FDXR levels was detectable in infected cells irrespective of the expression pattern of viral structural genes and proteins (Gag/Pol, p24, and gp46) or regulatory transcripts and proteins (Tax/Rex, Hbz, and Tax), as measured by qPCR and western blot (Appendix Fig. S1A–E). To further broaden the comparison, we analyzed RNA-Seq data from 259 blood cancer cell lines available in the Human Protein Atlas. The analysis confirmed FDXR expression as upregulated in HTLV-1-infected cells (Fig. 1E). In particular, the HTLV-1-infected cell lines (Hut-102, MJ, and C8166) in the dataset ranked among the top five overall for FDXR expression (permutation test *p* = 3.2e-06) (Fig. 1E).

To evaluate the physiological relevance of FDXR upregulation, we first reanalyzed previously published transcriptomic data of CD4 T cells and tumor samples from multiple cohorts, including Brazilian and Japanese patients. We conducted a meta-analysis of microarray datasets from four studies (Data ref: Araya et al, 2025; Data ref: Kodama et al, 2020; Data ref: Pinto et al, 2014; Data ref: Yamagishi et al, 2012a), each including samples from people living with HTLV-1 (PLHTLV), stratified by disease status, alongside healthy controls. Across all groups of PLHTLV, FDXR expression levels were elevated compared with healthy controls, reaching statistical significance in subgroup comparisons for Acute ATL, HAM/TSP, Chronic ATL, and asymptomatic carriers. Notably, FDXR expression mirrored disease severity (Fig. 1F). A similar pattern was observed upon reanalysis of RNA-Seq data derived from a dataset (Data ref: Kataoka et al, 2015a) including PLHTLV and healthy controls. Also in this case, median FDXR expression was higher in HTLV-1^+^ samples and increased with disease severity, showing statistically significant differences from healthy controls in Acute and Lymphoma ATL samples (Fig. 1G). In line with this, FDXR levels in ATL patients from this cohort showed a modest but significant (Spearman *r* = 0.355, *p* = 0.00755) correlation with proviral load (Appendix Fig. S2), which is a known predictor of disease severity and ATL progression (Iwanaga et al, 2010).

**Figure EV2:**
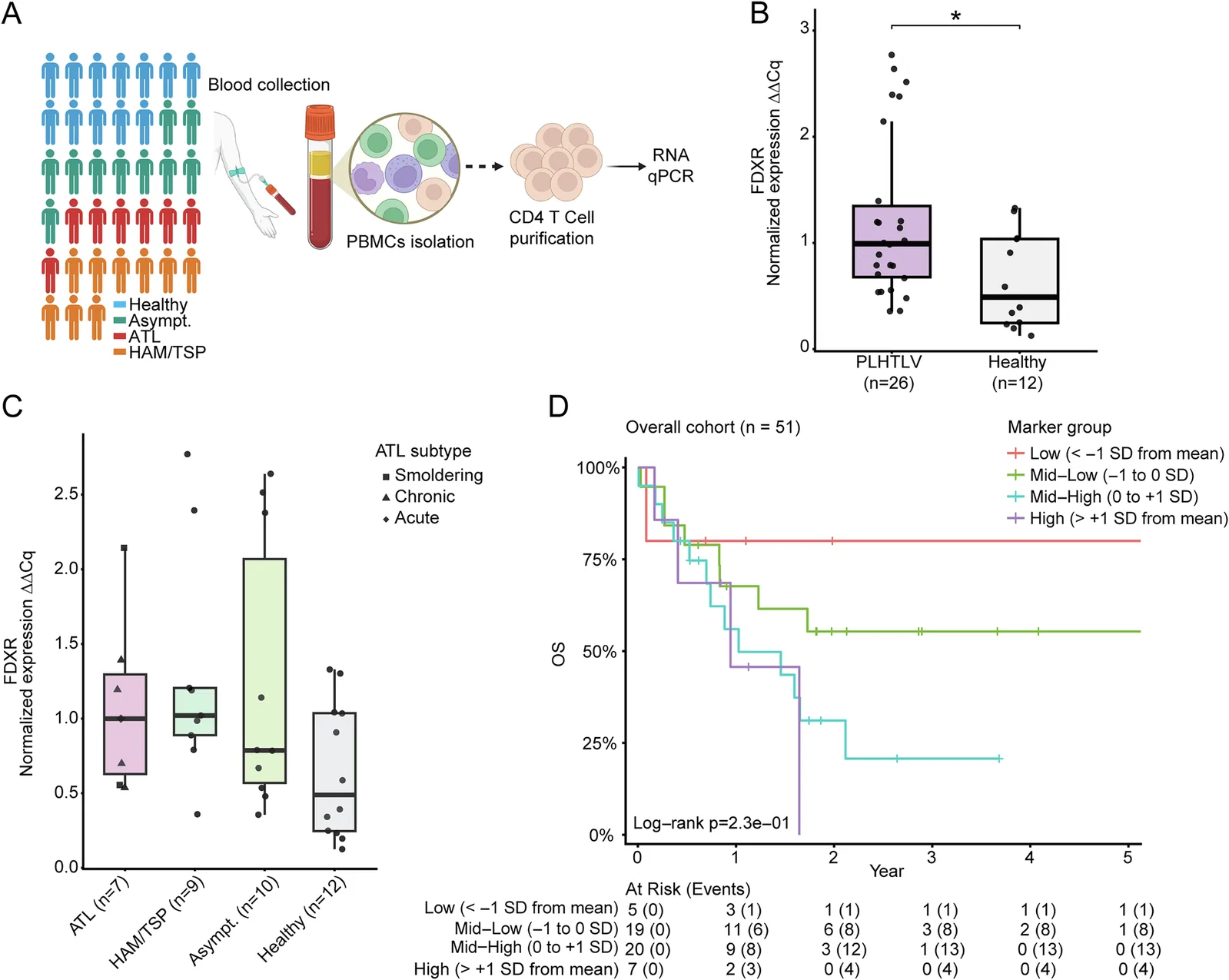
FDXR mRNA levels in PLHTLV and ATL survival curves by quartiles of FDXR expression. (**A**) CD4 T cells were isolated from PBMCs obtained from the cohort described in Table EV1. (**B**, **C**) Relative FDXR mRNA levels were calculated by ΔΔCq using TBP expression as a reference. Each dot represents an individual donor, while box plots show median ± IQR for each subgroup. In panel (**B**), differences in relative FDXR expression between PLHTLV and healthy controls were analyzed by an unpaired Wilcoxon rank-sum test following log_2_ transformation (*p* = 0.032). (**D**) Kaplan–Meier analysis of overall survival in patients grouped into quartiles based on the median Z-score of FDXR expression. FDXR expression values were derived from (Data ref: Kataoka et al, 2015a) and analysed in ATL patients with available survival data [*n* = 51 (Kataoka et al, 2015b; Kogure et al, 2022)]. Overall survival was censored at hematopoietic stem cell transplantation, and survival differences were assessed using the log-rank test. *p < 0.05.

For further orthogonal validation, we assessed FDXR expression by qPCR in CD4 T cells obtained from an independent, newly recruited Brazilian cohort of PLHTLV and healthy donors (Table EV1; Fig. EV2A–C). The results confirmed significantly higher FDXR expression in PLHTLV compared with healthy donors (Fig. EV2B). Although median FDXR levels also increased with disease severity in this cohort (Fig. EV2C), differences between clinical subgroups did not reach statistical significance, likely reflecting the smaller number of individuals with advanced disease (one acute ATL and nine HAM/TSP patients), compared with the other cohorts analyzed.

We next assessed the prognostic significance of FDXR expression levels on ATL patient survival by integrating RNA-Seq data (Data ref: Kataoka et al, 2015a) (Fig. 1G) with clinical outcome data available for 51 patients from the same cohort. A Cox model on continuous, z-score-normalized, FDXR expression revealed a significant link between higher FDXR levels and reduced survival (HR = 1.7, *P* = 0.034; Fig. 1H). Incorporating ATL subtype stratification (aggressive [acute/lymphoma] and indolent [chronic/smoldering] subtypes) further showed that FDXR expression was significantly associated with worse prognosis within indolent subtypes of ATL (HR = 5.5, *P* = 0.014; Fig. 1H). This likely reflects the broader dynamic range of FDXR expression in indolent ATL (Fig. 1F,G), that can enable greater prognostic discrimination than in aggressive disease, where FDXR upregulation and clinical outcomes are more uniform. Finally, Kaplan–Meier survival analyses based on two- and four-group stratifications of FDXR expression also demonstrated poorer outcomes among patients with higher FDXR levels, reaching statistical significance in the two-group comparison (Figs. 1I and EV2D).

Overall, these results show that FDXR expression levels in PLHTLV correlate with disease progression and worse ATL prognosis.

### FDXR upregulation is driven by ectopic expression of TAp63 and TAp73

**Figure 2:**
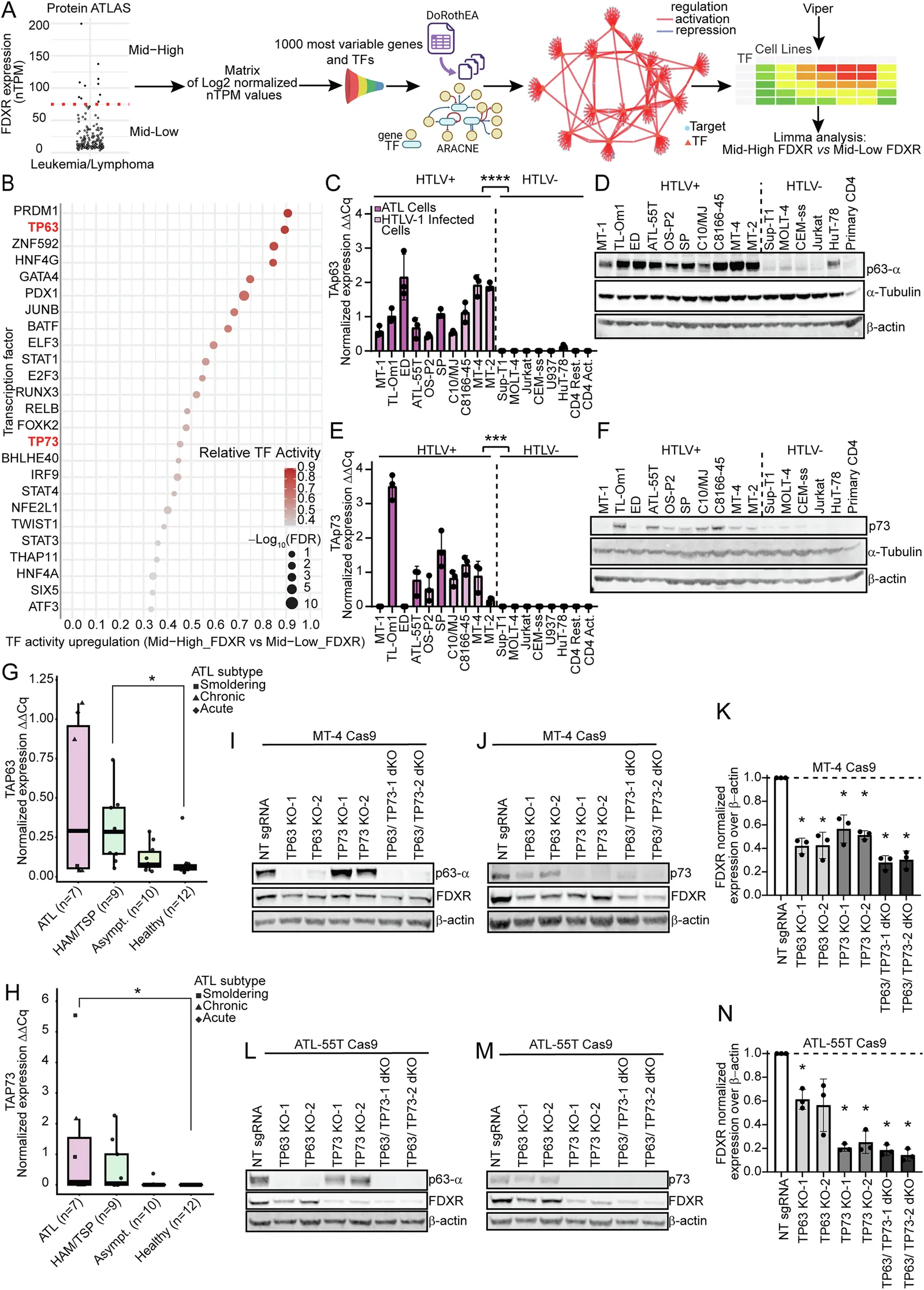
Regulation of FDXR by TAp63 and TAp73 in HTLV-1^+^/ATL cells. (**A**, **B**) RNA-Seq data retrieved from Protein Atlas were stratified by FDXR expression (≥75 nTPM = Mid-high_FDXR, <75 nTPM = Mid-low_FDXR). Differential transcription factor (TF) activity between groups was inferred using VIPER (Alvarez et al, 2016), based on regulatory networks constructed with DoRothEA (Garcia-Alonso et al, 2019) and ARACNe. The bubble plot in (**B**) ranks TFs based on their differential activity in the two groups. The color of the dots reflects relative TF activity, while the size of the dots represents −log10 adjusted *p* values (FDR) calculated with the limma package. (**C**–**F**) Expression of TAp63 mRNA (**C**), p63α protein (**D**), TAp73 mRNA (**E**), and p73 protein (**F**) in HTLV-1^+^, ATL, and uninfected cell lines and primary cells. Relative mRNA expression was quantified by qPCR; protein expression was assessed by western blot. In panels (**C**, **E**), each dot represents an independent qPCR assay (*n* = 3), and data were presented as mean ± SD. Relative mRNA levels were calculated by ΔΔCq using TBP expression as a reference. Data were analyzed by the nonparametric Wilcoxon rank-sum test (*p* = 4.57e-05 in (**C**) and 4.11e-04 in (**E**)). (**G**, **H**) Relative expression of TAp63 (**G**) and TAp73 (**H**) mRNA in primary CD4 T cells from PLHTLV stratified by disease condition and healthy controls. Relative mRNA expression was quantified by qPCR using the ΔΔCq method and TBP expression as reference. Each dot represents a donor, and data are depicted as median ± IQR. Data were analyzed by the nonparametric Kruskal–Wallis test followed by Dunn’s post hoc test with Benjamini–Hochberg FDR correction. In (**G**): ATL *p* = 0.12; HAM/TSP *p* = 0.02; Asymptomatic *p* = 0.38. In (**H**): ATL *p* = 0.03; HAM/TSP *p* = 0.09; Asymptomatic *p* = 0.41. (**I**–**N**) Effect of *TP63* and *TP73* knockout on FDXR expression. The HTLV-1-infected MT-4 cell line (**I**–**K**) and the ATL-derived ATL-55T cell line (**L**–**N**) were stably transfected with Cas9 and subsequently transduced with lentiviruses expressing either a non-targeting (NT) sgRNA or sgRNAs targeting *TP63* and/or *TP73*. Stable knockout cell lines were lysed and used to assess protein expression by Western blot. Expression of FDXR and either p63 or p73 is shown in MT-4 (**I**, **J**) and ATL-55T (**L**, **M**) cells, respectively. Panels (**K**, **N**) show relative FDXR protein levels normalized to actin as a loading control and to the NT sgRNA condition as the reference. Bar plots depict mean ± SD for each condition (*N* = 3). Data were analysed by one-sample *t*-tests, followed by a Benjamini–Hochberg FDR correction. In (**K**): TP63_1 *p* = 0.024; TP63_2 *p* = 0.041; TP73_1 *p* = 0.046; TP73_2 *p* = 0.024; TP63 + TP73_1 *p* = 0.024; TP63 + TP73_2 *p* = 0.024. In N: TP63_1 *p* = 0.029; TP63_2 *p* = 0.139; TP73_1 *p* = 0.016; TP73_2 *p* = 0.029; TP63 + TP73_1 *p* = 0.019; TP63 + TP73_2 *p* = 0.02. ****p < 0.0001; ***p < 0.001; *p < 0.05. Source data are available online for this figure.

HTLV-1 infection is known to dysregulate the activity and expression of multiple host genes, including transcription factors (TFs) (Cook et al, 2017). To identify putative host mediators of FDXR upregulation, we first conducted an in silico analysis of differential TF activity across leukemia and lymphoma cell lines, including HTLV-1-infected models, using RNA-Seq data retrieved from the Human Protein Atlas. Cells were stratified in two groups according to FDXR expression levels (Fig. 2A). TF activity was inferred from downstream target gene expression using the VIPER algorithm (Alvarez et al, 2016) in combination with a regulatory network of TF-target interactions derived from curated databases [DoRothEA (Garcia-Alonso et al, 2019)] and interactions inferred directly from the dataset (Meyer et al, 2008) (Fig. 2A). The results highlighted the TF activity of two members of the p53 family, p63 and p73, as significantly upregulated in cells expressing higher FDXR levels (Fig. 2B). Of note, p63 activity was the second most upregulated, ranking after PRDM1, which encodes for a transcriptional repressor of p53 (Yan et al, 2007). This pattern suggests a potential shift away from p53 dependence toward increased reliance on the p63 and p73 members of the family.

**Figure EV3:**
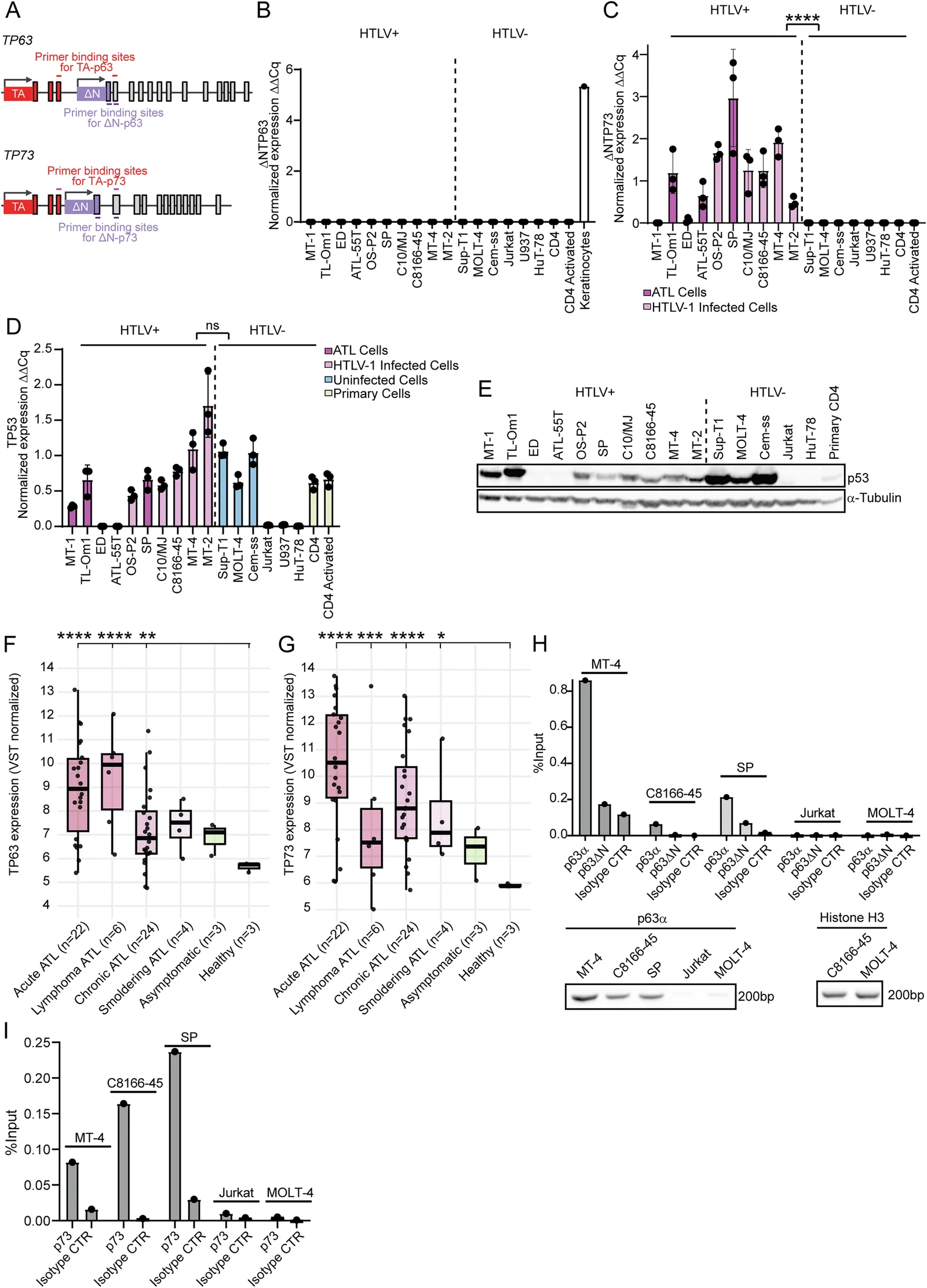
p53 family expression profiles and chromatin binding at the FDXR promoter in HTLV-1^+^/ATL and uninfected cells. (**A**) Isoform-specific primer design for amplification of TA and ΔN isoforms of TP63 and TP73 by qPCR. (**B**, **C**) Relative mRNA expression of ΔN isoforms of TP63 (**B**) and TP73 (**C**) in HTLV-1^+^, ATL, and uninfected cell lines and primary cells, as assessed by qPCR. In (**B**), primary keratinocytes were used as a positive control for ΔNTP63 expression. (**D**, **E**) Relative mRNA (**D**) and protein (**E**) expression of p53 in HTLV-1^+^, ATL, and uninfected cell lines and primary cells, as assessed by qPCR and western blotting, respectively. In panels (**B**–**D**), each dot represents an independent qPCR assay (*n* = 3), and data were presented as mean ± SD. Relative mRNA levels were calculated by ΔΔCq using TBP expression as a reference. Data were analyzed by the nonparametric Wilcoxon rank-sum test (*p* = 4.57e-05 in (**C**) and *p* = 0.94 in (**D**). (**F**, **G**) TP63 and TP73 RNA expression in tumor and CD4 T cells isolated from ATL (Acute *n* = 22; Chronic *n* = 24; Lymphoma *n* = 6; Smoldering *n* = 4), asymptomatic HTLV-1 carriers (*n* = 3), and healthy controls (*n* = 3). RNA-Seq data (Data ref: Kataoka et al, 2015a) are plotted for each donor as variance-stabilized (VST) expression, while box plots show median ± IQR for each subgroup. Differential expression between each subgroup and healthy controls was tested using DESeq2. In (**F**): Acute ATL *p* = 1.23e-05; Lymphoma ATL *p* = 9.59e-05; Chronic ATL *p* = 0.008; Smoldering ATL *p* = 0.173; Asymptomatic *p* = 0.551. In (**G**): Acute ATL *p* = 3.75e-07; Lymphoma ATL *p* = 1.4e-04; Chronic ATL *p* = 9.06e-05; Smoldering ATL *p* = 0.015; Asymptomatic *p* = 0.564. (**H**, **I**) ChIP–qPCR enrichment over input showing p63 (α isoform) (**H**) and p73 (**I**) binding to the p53-responsive element (Liu and Chen, 2002) within the FDXR promoter in HTLV-1^+^ cells and uninfected controls. Chromatin immunoprecipitated with anti-ΔNp63 or IgG antibodies was used as a control. Amplification products were analyzed by agarose gel electrophoresis, as shown in the lower panel (**H**). Primer sequences used to amplify the p53-responsive element are listed in Table EV3. *p < 0.05, **p < 0.01, ***p < 0.001, ****p < 0.0001.

The FDXR promoter harbors a responsive element that can be bound by all members of the p53 family (Liu and Chen, 2002), potentially providing a direct mechanistic link with FDXR upregulation. We thus examined their expression in HTLV-1^+^/ATL cell lines, as well as in primary CD4 T cells isolated from PLHTLV, using uninfected cell lines and primary CD4 T cells as controls. Because only the TA isoforms of p63 and p73, which retain an intact N-terminal transactivation domain, have been reported to bind the FDXR promoter in colorectal cancer cells (Liu and Chen, 2002; Murray-Zmijewski et al, 2006), we quantified isoform-specific expression using qPCR assays designed to distinguish TA from ΔN variants (Fig. EV3A). TAp63 mRNA and p63 protein were consistently expressed in all HTLV-1^+^/ATL cell models, whereas uninfected cells largely lacked TAp63, with only modest expression detected in Hut-78 cells (Fig. 2C,D). Similarly, TAp73 mRNA and p73 protein were upregulated in HTLV-1^+^/ATL cells, consistent with previous reports (Toyoda et al, 2023), with the exception of MT-1 and ED cells (Fig. 2E,F), which are also characterized by low or absent FDXR upregulation (Fig. 1B,C). ΔNp63 isoforms were undetectable across all models (Fig. EV3B), while ΔNp73 expression mirrored that of TAp73 (Fig. EV3C). In contrast, p53 mRNA and protein levels showed no significant differences between infected and uninfected cells (Fig. EV3D,E), in agreement with our TF activity analysis. Consistent with these findings, reanalysis of the same RNA-Seq dataset used to assess FDXR upregulation in ATL subtypes (Data ref: Kataoka et al, 2015a) also revealed significantly increased expression of TP63 and TP73 in Acute, Lymphoma and Chronic ATL patients (Fig. EV3F,G). Moreover, qPCR analysis of primary CD4 T cells of our independent cohort of PLHTLV (Table EV1) showed TAp63 and TAp73 as significantly increased in HAM/TSP and ATL patients, respectively (Fig. 2G,H).

To further elucidate the mechanistic link between TA isoform expression and FDXR transcription, we performed chromatin immunoprecipitation (ChIP) followed by qPCR using primers specific for the p53-responsive element within the FDXR promoter (Liu and Chen, 2002). In line with expression patterns, TAp63 showed specific binding to the FDXR promoter in HTLV-1^+^/ATL cells, whereas ΔNp63 showed minimal or no enrichment above IgG controls (Fig. EV3H). Moreover, chromatin precipitated with an anti-p73 antibody also displayed binding to the FDXR promoter in HTLV-1^+^/ATL cells, but not in uninfected cells (Fig. EV3I).

Finally, to provide a direct mechanistic link between FDXR upregulation and p63/p73 expression, we generated stable CRISPR-Cas9 knockouts in MT-4 (Fig. 2I–K) and ATL-55T (Fig. 2L–N) cells. In both models, loss of either p63 or p73 significantly reduced FDXR protein levels, with double p63/p73 knockout cells showing the greatest reduction (Fig. 2I–N).

Taken together, these data demonstrate that FDXR upregulation in HTLV-1^+^/ATL cells is driven by selective expression of TAp63 and TAp73.

### The IRF4-BATF3 transcriptional complex regulates p63 expression

**Figure 3:**
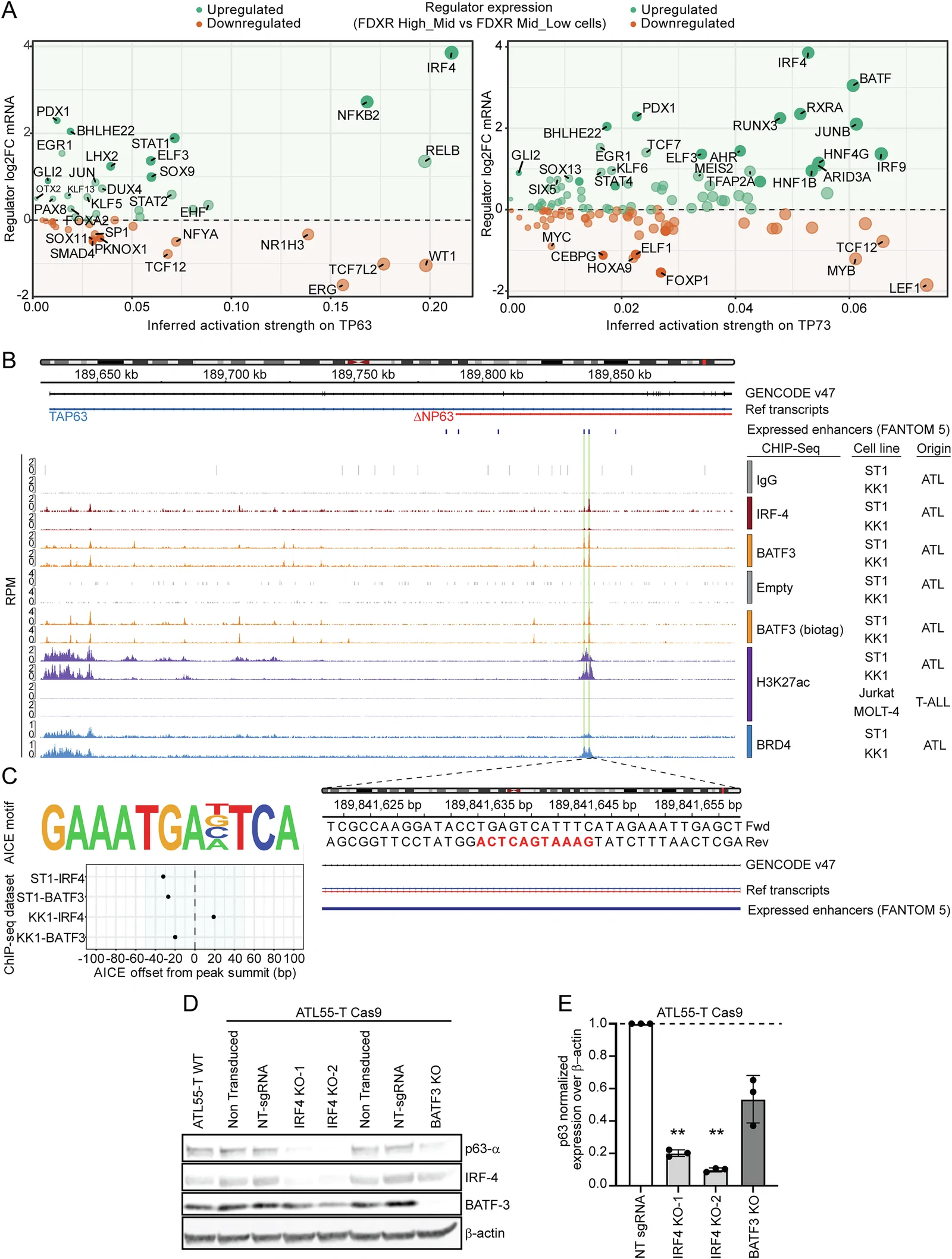
IRF4/BATF3 binding to the *TP63* locus drives p63 expression in ATL cells. (**A**) RNA-seq data from leukemia and lymphoma cell lines were retrieved from the Human Protein Atlas, stratified by FDXR expression (≥75 nTPM, Mid-High_FDXR; <75 nTPM, Mid-Low_FDXR), and used to infer a transcriptional regulatory network (regulon) as depicted in Fig. 2A. Upstream transcriptional activators of TP63 (left) and TP73 (right) were extracted from this regulon. Scatter plots display, for each activator, the inferred regulatory effect on TP63 or TP73 (x-axis) versus its differential mRNA expression between mid-high and mid-low FDXR groups (y-axis), estimated using limma (Ritchie et al, 2015). Colors indicate the direction of differential mRNA expression of each activator (green, upregulated; red, downregulated), and point size indicates regulatory strength. The top 30 transcriptional activators for TP63 and TP73 are labeled. (**B**, **C**) ChIP-seq signal tracks and MACS2 peak calls (*q* = 1e-5) for IRF4, BATF3, biotag-BATF3, H3K27ac, and BRD4 at the *TP63* locus were retrieved from published datasets in ChIP-Atlas (Zou et al, 2024). Panel (**B**) shows the IGV visualization of RPM-normalized ChIP-seq signal tracks for ATL cell lines (Data ref: Nakagawa et al, 2018b) (ST1 and KK1) and uninfected lymphoid cell lines (Jurkat and MOLT-4). MANE (Morales et al, 2022) (v1.0; Ensembl) annotations display reference transcripts for TAp63 and ΔNp63, while FANTOM5 (Arner et al, 2015) annotations indicate experimentally defined expressed enhancer regions. Regions highlighted in light green correspond to active enhancer elements co-occupied by IRF4 and BATF3 and marked by H3K27ac and BRD4 enrichment in ATL cells. The lower zoom-in panel shows the sequence and genomic position of a canonical AICE motif within an active enhancer region. Genomic coordinates are based on the hg38 assembly. Panel (**C**) shows the distance between the AICE motif occurring within the *TP63* locus and the summits of IRF4 and BATF3 ChIP-seq peaks in ST1 and KK1 cells. Distances were calculated using HOMER (Li et al, 2009) by scanning 100-bp windows centered on ChIP-seq peak summits with three previously described (Glasmacher et al, 2012) canonical AICE motif sequences. The sequence logo depicts the AICE motif variant identified within the *TP63* locus. (**D**, **E**) Effect of IRF4 and BATF3 knockout on p63 expression. The ATL-55T-Cas9 cell line was transduced with lentiviruses expressing either a non-targeting (NT) sgRNA or sgRNAs targeting IRF4 or BATF3. The transduced cells were used to assess protein expression by Western blot. Panel (**E**) shows the relative p63 protein levels normalized to actin as a loading control and to the NT sgRNA condition as the reference. Bar plots depict mean ± SD for each condition (*N* = 3). Data were analysed by one-sample *t*-tests, followed by a Benjamini–Hochberg correction. In (**E**): IRF4_1 *p* = 0.002; IRF4_2 *p* = 0.002; BATF3_1 *p* = 0.062. **p < 0.01. Source data are available online for this figure.

Building on previous reports that p73 expression in ATL cells can be induced by the IRF4/BATF3 (Nakagawa et al, 2018c; Toyoda et al, 2023) complex, we investigated whether p63 can also serve as a target for this complex. To this end, we extended our in silico analysis of TF activity (Fig. 2A) to identify upstream regulators of TP63 and TP73. Strikingly, IRF4 emerged as one of the strongest predicted upstream activators of both genes (Fig. 3A). Moreover, analysis of ChIP-seq data generated in ATL cells (Data ref: Nakagawa et al, 2018b) revealed binding peaks for both BATF3 and IRF4 at the *TP63* locus (Fig. 3B). Scanning the sequence of the *TP63* locus revealed a canonical AP1-IRF composite (AICE) motif (Fig. 3C), belonging to a class of regulatory elements known to mediate cooperative binding of IRF4 and BATF3 (Murphy et al, 2013). The AICE motif lay within 50 bp of IRF4 and BATF3 binding peaks (Fig. 3C), overlapped with an active enhancer [FANTOM5 collection (Arner et al, 2015; Murphy et al, 2013)] and was marked by enrichment of H3K27ac and BRD4 binding. Overall, this was consistent with open chromatin and active enhancer activity (Fig. 3B).

In line with this, parallel analysis of CHIP-Seq and transcriptomic data in ATL cells (Data ref: Nakagawa et al, 2018a; Data ref: Nakagawa et al, 2018b) showed reduction of TP63 expression upon IRF4 or BATF3 knockdown (Appendix Fig. S3A), as well as after displacement of BRD4 upon treatment with JQ1 (Appendix Fig. S3B,C). Moreover, when we generated CRISPR-Cas9 knockouts of IRF4 or BATF3 in ATL-55T cells we observed markedly decreased protein levels of p63 (Fig. 3D,E). This reduction was statistically significant following IRF4 sgRNA treatment and approached significance following BATF3 sgRNA treatment (adjusted *p* value = 0.06) (Fig. 3E).

Taken together, these data show that the IRF4-BATF3 complex can directly bind to the *TP63* locus and induce p63 expression in ATL cells.

### FDXR transcription is enriched within the proliferating cell cluster of ATL patients

**Figure 4:**
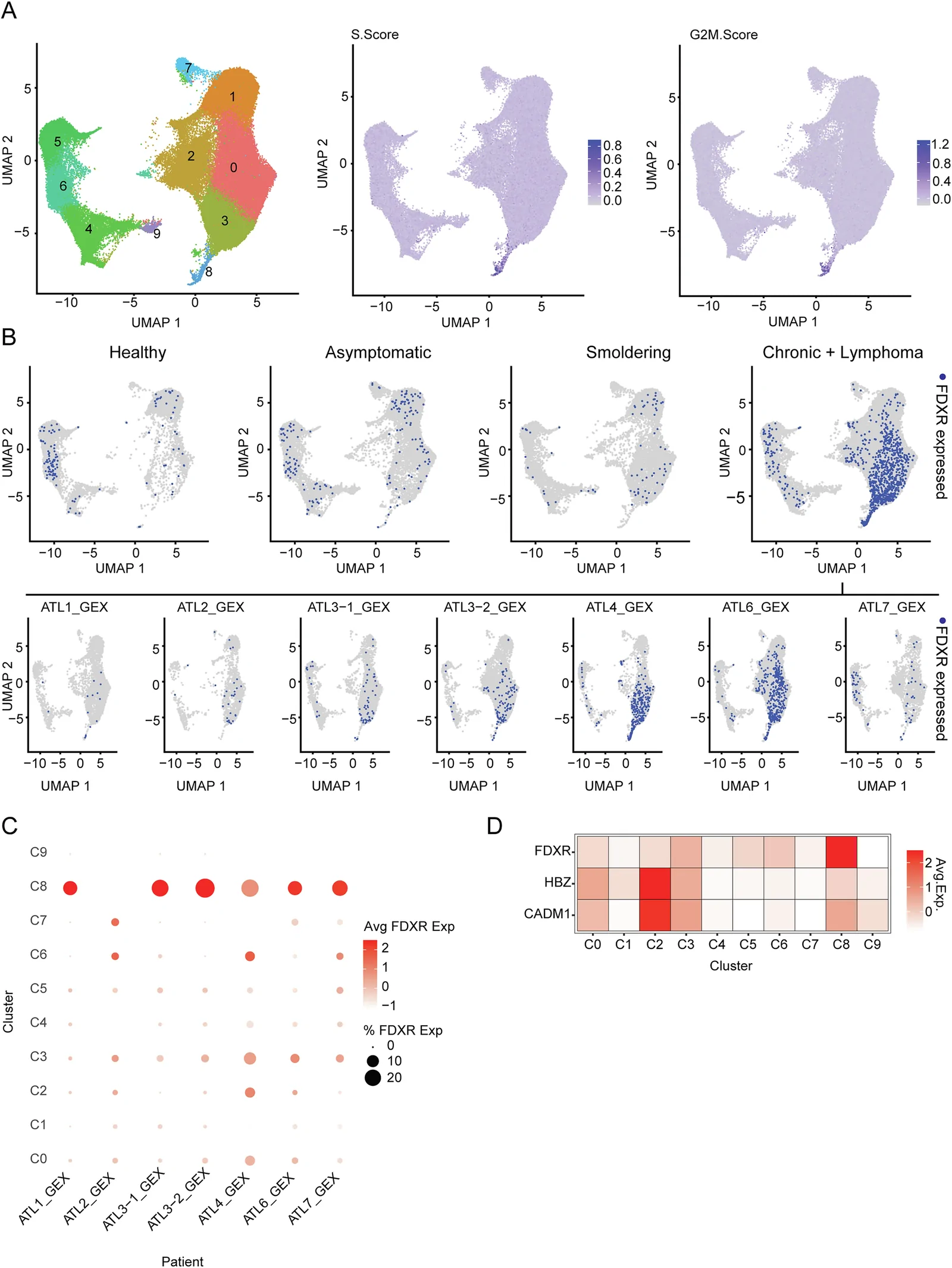
Single-cell FDXR expression pattern in blood cells of PLHTLV. Raw scRNA-seq data were retrieved from the study (Data ref: Tan et al, 2021a), comprising 17 samples from 16 donors (three healthy donors, four asymptomatic HTLV-1 carriers, three smoldering ATL, five chronic ATL, and one lymphoma-type ATL). (**A**) UMAP visualization of 10 T/NK cell clusters (C0–C9) identified across the dataset (91,436 cells total). Proliferating cells are highlighted by S-phase and G2/M-phase cell-cycle scores, computed with Seurat. (**B**) Distribution of FDXR-expressing cells across sample categories: healthy donors (average FDXR^+^ = 1.20%), asymptomatic carriers (average FDXR^+^ = 0.75%), smoldering ATL (average FDXR^+^ = 0.47%), and chronic/lymphoma ATL (average FDXR^+^ = 2.29%). The UMAP visualizations of individual ATL samples (ATL1–ATL7) display the localization of FDXR-expressing cells within the T/NK subset of each sample. (**C**) Dot-plot showing the frequency and relative expression levels of FDXR across clusters (C0–C9) in each ATL sample. (**D**) Heatmap comparing in ATL samples the average expression of FDXR and of the known infection markers (Manivannan et al, 2016; Tan et al, 2021b) HBZ and CADM1. The heatmap shows scaled average expression levels of each gene across clusters (C0–C9). The expression of each gene within each cluster was quantified by aligning reads to a custom reference transcriptome generated by merging the human GRCh38 genome with the HTLV-1 genome (GenBank: AB513134.1). Cluster 8 denotes proliferating cells (Appendix Fig. S5). Source data are available online for this figure.

In addition to its role in maintaining mitochondrial homeostasis, FDXR can act as a negative regulator of excessive cell proliferation (Zhang et al, 2017). We thus hypothesized that, in ATL, increased FDXR expression may occur preferentially in response to tumor cell proliferation. We examined the single-cell expression pattern of FDXR in primary cells, leveraging a previously published scRNA-seq dataset generated using PBMCs of healthy donors and PLHTLV, including ATL patients (Data ref: Tan et al, 2021a). After dimensionality reduction and clustering (Fig. 4A), we focused our analysis on ten clusters of T/NK cells (Appendix Fig. S4) from individuals with chronic ATL (samples ATL-1, -2, -3, -6, and -7) and lymphoma (sample ATL-4). These samples showed the highest average frequency of cells with active *FDXR* gene expression (Fig. 4B), consistent with our observation that FDXR upregulation increases with disease severity (Fig. 1F,G).

Single-cell analysis further revealed enrichment of active *FDXR* gene expression within the proliferating cell cluster (cluster 8) in all but one ATL sample (Fig. 4C). The proliferative identity of this cluster was supported by its overlap with cells in the S and G2/M phases of the cell cycle (Fig. 4A) and by the expression of canonical proliferation-associated marker genes (Appendix Fig. S5). The relative expression of FDXR was more enriched in proliferating cluster 8 as compared to that of CADM1 and Hbz (Fig. 4D), which are established markers of HTLV-1 infection and ATL (Manivannan et al, 2016; Tan et al, 2021b). This is in line with a specific role of FDXR in the antiproliferative response affecting both infected and uninfected tumor cells. However, expression of FDXR and its upstream regulators, TP63 and TP73, also displayed a modest but significant correlation with CADM1 expression (Table EV2). At a single cell level, TP73 additionally correlated with HBZ expression (Table EV2), consistent with previous findings (Toyoda et al, 2023). These analyses support our bulk RNA expression results (Figs. 1F,G and 2G,H) and suggest that, in addition to its enrichment in proliferating cells, FDXR and its upstream transcription factors are preferentially expressed in HTLV-1 infected cells, although not necessarily in cells with active viral expression.

Overall, this analysis shows that FDXR expression is selectively enriched at the single-cell level in proliferating cells from ATL patients.

### FDXR expression levels predict sensitivity to selected chemotherapeutics in vitro and ex vivo

**Figure 5:**
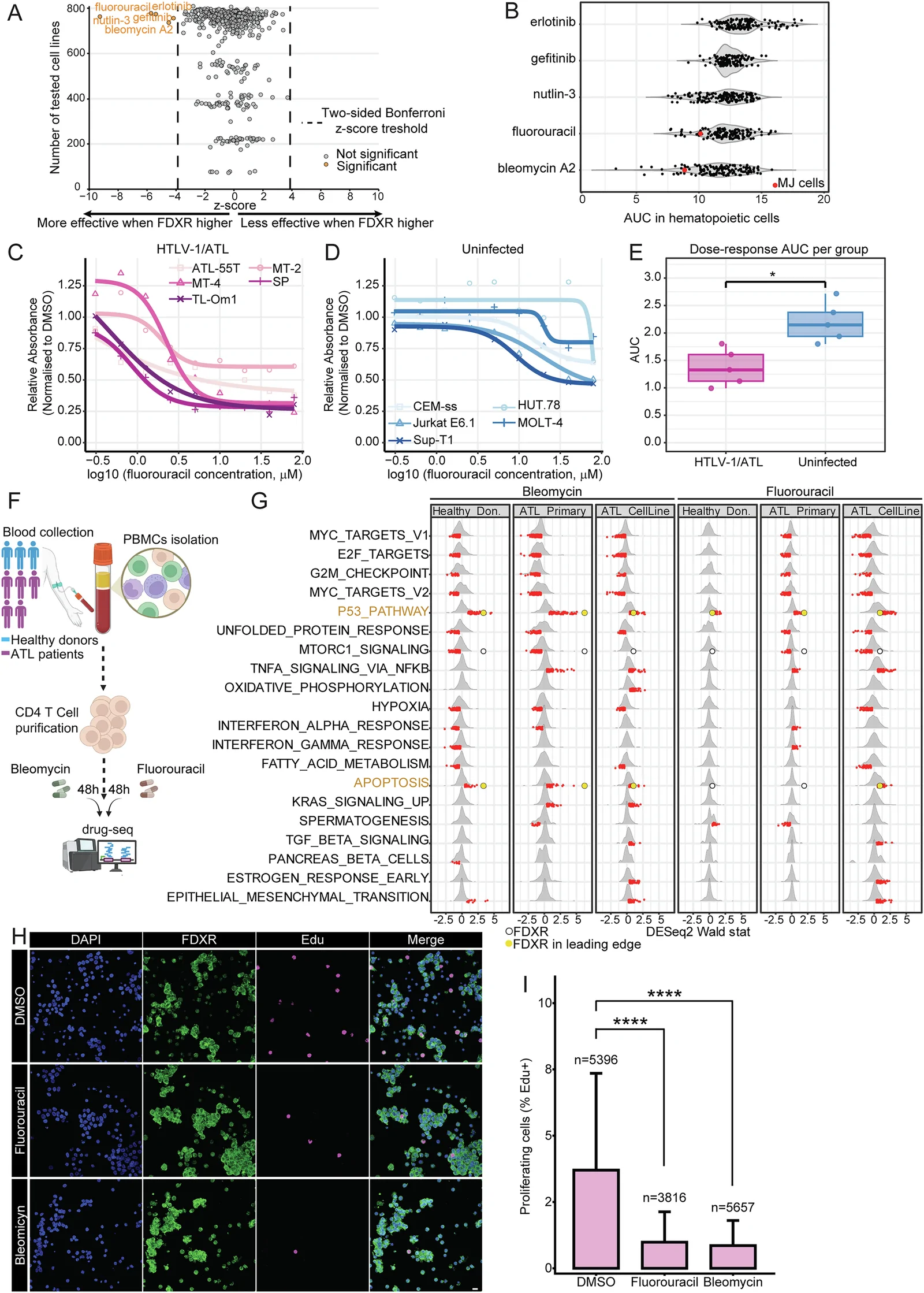
Chemotherapeutics leveraging FDXR expression in HTLV-1^+^/ATL cells. (**A**, **B**) Data from the Cancer Target Discovery and Development (CTD²) Network (Rees et al, 2016; Seashore-Ludlow et al, 2015) were used to assess associations between FDXR mRNA expression and sensitivity to 481 compounds across cancer cell lines (up to 835 cell lines per compound). In panel (**A**), the scatter plot shows the association between FDXR expression and drug sensitivity for each compound, expressed as a Pearson correlation z-score (x-axis) versus the number of cell lines tested (y-axis). Significance thresholds were defined using a Bonferroni-corrected two-tailed z-test. Panel (**B**) shows the distribution of area under the curve (AUC) values in hematological and lymphoid cell lines for compounds significantly correlated with FDXR expression. Lower AUC values indicate increased drug sensitivity. Each dot represents a cell line, with the HTLV-1-infected MJ cell line highlighted in red. (**C**–**E**) HTLV-1^+^/ATL and uninfected cell lines were treated with serial dilutions of fluorouracil, and cell viability/proliferation was assessed by MTS assay 72 h post-treatment. Cell models were tested in two independent experiments, each performed in technical triplicate. Mean absorbance values were normalized to DMSO controls, and dose-response curves were fitted for each cell line using four-parameter logistic nonlinear regression (**C**, **D**). The fitted curves were used to calculate the AUCs depicted in panel (**E**). In panel (**E**), each dot represents a cell line, and data are depicted as median ± IQR. Group-wise AUC comparisons were conducted using an unpaired Wilcoxon rank-sum test (*p* = 0.016). (**F**, **G**) Primary CD4 T cells isolated from five ATL patients (one acute, three chronic, one smoldering) and three healthy donors, as well as two ATL cell lines (SP and TL-Om1), were treated with fluorouracil or bleomycin for 48 h. DMSO-treated samples served as parallel controls. RNA was extracted and subjected to Drug-Seq analysis (**F**). Ridge plots in panel (**G**) show the distribution of DESeq2 Wald statistics for genes within the top 20 Hallmark pathways (top 10 per drug by maximum |NES|) as calculated by GSEA (Fig. EV4D). Red ticks indicate leading-edge genes. FDXR is highlighted in gold when present in the leading edge or indicated by an open circle when present in a pathway but not in the leading edge. (**H**, **I**) Primary CD4 T cells isolated from three chronic ATL patients were treated with fluorouracil or bleomycin (10 μM for each drug) for 72 h. Cells were fixed with 4% PFA and stained with the Click-iT Plus EdU kit to detect proliferation as well as with an anti-FDXR antibody. Panel H displays representative z-projection images showing nuclei (DAPI, blue), FDXR (green), and proliferating cells identified by EdU incorporation (magenta). Scale bar: 10 μm. The barplot in panel I shows the percentage (mean ± SD) of proliferating (EdU-positive) cells. The total number of cells analyzed per condition is indicated above each bar. Data were analyzed using a binomial generalized linear mixed-effects model with donor as a random effect and Dunnett-adjusted comparisons to DMSO (fluorouracil *p* = 1.36e-06; bleomycin *p* = 6.61e-08). *p < 0.05; ****p < 0.0001. Source data are available online for this figure.

Baseline FDXR expression and its upregulation upon treatment has been linked to increased sensitivity to ROS-generating chemotherapeutics in colorectal cancer (Hwang et al, 2001; Liu and Chen, 2002). To broadly assess which chemotherapeutics could leverage increased FDXR expression, we analysed cytotoxicity and gene-expression profiles from the Cancer Therapeutics Response Portal (CTRP) (Rees et al, 2016). The analysis of the entire dataset (481 compounds tested in up to 835 cell lines) revealed that FDXR expression was significantly associated with enhanced sensitivity to five agents (Fig. 5A). Restricting the analysis to hematopoietic cell lines identified fluorouracil and bleomycin A2 as the compounds with the lowest dose-response area under curve (AUC) values, a pattern also observed in the only HTLV-1 infected cell line (MJ cells) in the dataset (Fig. 5B).

**Figure EV4:**
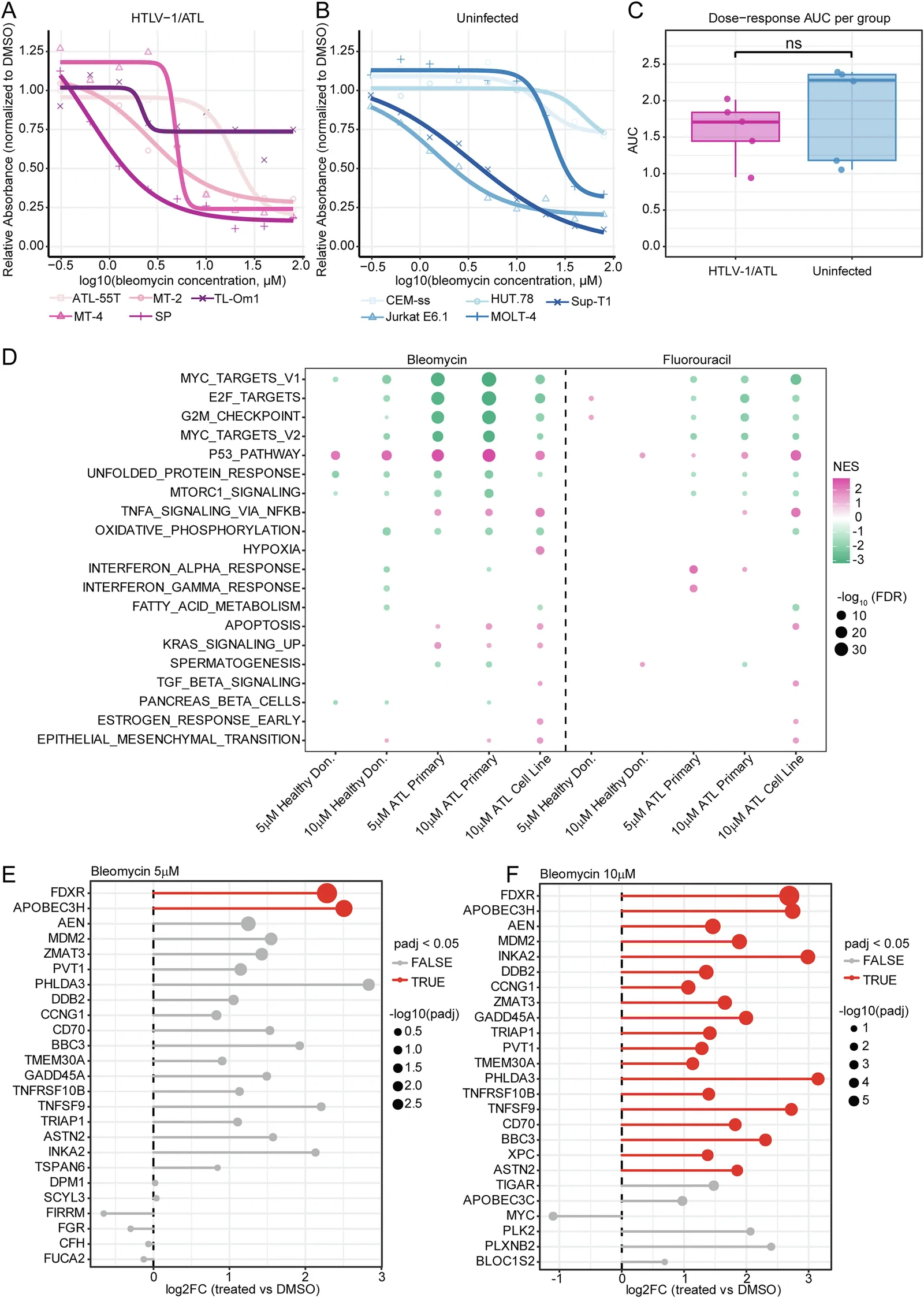
Dose-dependent effects of bleomycin and fluorouracil in vitro and ex vivo. (**A**–**C**) HTLV-1^+^/ATL and uninfected cell lines were treated with serial dilutions of bleomycin, and cell viability/proliferation was assessed by MTS assay 72 h post-treatment. Cell models were tested in two independent experiments, each performed in technical triplicate. Mean absorbance values were normalized to DMSO controls, and dose-response curves were fitted for each cell line using four-parameter logistic nonlinear regression (**A**, **B**). The fitted curves were used to calculate AUCs (**C**). In panel (**C**), each dot represents a cell line, and data were depicted as median ± IQR. Group-wise AUC comparisons were conducted using an unpaired Wilcoxon rank-sum test. (**D**–**F**) Primary CD4 T cells from five ATL patients (one acute, three chronic, one smoldering) and three healthy donors, along with two ATL cell lines (SP and TL-Om1), were treated with fluorouracil or bleomycin for 48 h. RNA was extracted and analyzed by Drug-Seq. Panel (**D**) shows treatment-induced pathway changes evaluated by gene set enrichment analysis (GSEA) (Subramanian et al, 2005) using Hallmark gene sets, based on DESeq2 Wald statistics comparing drug-treated samples with DMSO controls. Bubble size indicates enrichment significance (−log_10_ FDR), and color indicates normalized enrichment score (NES). The top 20 pathways (top 10 per drug by maximum |NES|) are shown for each condition. Panels (**E**, **F**) show lollipop plots of the top 25 genes altered by bleomycin treatment at 5 μM (**E**) and 10 μM (**F**) in primary ATL cells, based on DESeq2 analysis. Genes are ranked by statistical significance (adjusted *p* value). For each gene, the stem and point position indicate the log₂ fold change relative to DMSO. Point size reflects −log_10_ (adjusted *p* value), and point color denotes significance (adjusted *p* < 0.05).

Both fluorouracil and bleomycin are approved chemotherapeutic agents; however, neither has been evaluated in ATL to date. We therefore assessed their effects on cell viability and proliferation by MTS assay in HTLV-1^+^/ATL cell models, using uninfected lymphoid cells as controls. Fluorouracil demonstrated increased efficacy in HTLV-1^+^/ATL cell lines, as indicated by significantly lower dose-response AUC values (Fig. 5C–E). Treatment with bleomycin was also associated with lower median dose-response AUC values in HTLV-1^+^/ATL cell lines, although this difference was not statistically significant (Fig. EV4A–C).

We next evaluated the effects of fluorouracil and bleomycin in primary ATL cells, by performing Drug-Seq on CD4 T cells isolated from five ATL donors (one acute, three chronic, and one smoldering) and three healthy donors, all from our newly recruited cohort in Brazil (Table EV1; Fig. 5F). Two ATL cell lines (SP and TL-Om1) were also assayed as controls. Each drug was tested at two concentrations (5 and 10 μM). Gene set enrichment analysis using Hallmark pathways revealed that bleomycin significantly perturbed a greater number of pathways as compared to fluorouracil (Figs. 5G and EV4D). However, fluorouracil treatment in primary ATL cells and ATL cell lines affected a larger number of pathways as compared with cells of healthy donors (Figs. 5G and EV4D), in line with its higher selectivity in dose-response MTS experiments (Fig. 5C–E). Both bleomycin and fluorouracil significantly affected pathways associated with cell proliferation and cell death, particularly the p53 pathway (Figs. 5G and EV4D). Of note, FDXR expression was induced by both treatments and was identified as a leading-edge gene in the p53 and apoptosis pathways, consistent with its involvement in treatment-induced antiproliferative and cell-death responses (Fig. 5G). Although in primary cells the pathway-level effects of bleomycin were less selective than those of fluorouracil (Figs. 5G and EV4D), at the single gene level, FDXR was the most significant differentially expressed gene (DEG) in primary ATL cells treated with each bleomycin concentration (Fig. EV4E,F).

Finally, to validate the antiproliferative effects associated with FDXR upregulation following exposure to fluorouracil and bleomycin, we combined immunofluorescence staining for FDXR with Click-EdU incorporation assays in primary CD4 T cells from ATL donors from the same cohort (Table EV1). The results showed a significant reduction in the frequency of proliferating cells upon treatment with either fluorouracil or bleomycin as compared to DMSO controls (Fig. 5H,I).

Overall, these data indicate that repurposed chemotherapeutics leveraging FDXR expression can inhibit the proliferation of ATL cells in vitro and ex vivo.

## Discussion

Our findings identify FDXR upregulation as a novel marker of HTLV-1 infection, as well as disease severity and prognosis, thus extending its role beyond solid cancers. In colorectal cancer, FDXR has been linked to p53-dependent pro-apoptotic activity (Hwang et al, 2001) and proposed as a biomarker for predicting treatment outcome (Ichikawa et al, 2006). A similar approach may be applicable to ATL, provided the development of a standardized assay to define FDXR upregulation thresholds. In terms of disease outcome, the prognostic value of FDXR upregulation in a retrospective ATL cohort was significant only in indolent cases (chronic and smoldering). This pattern likely reflects the distribution of FDXR expression across ATL subtypes. While most acute and lymphoma cases exhibit elevated FDXR levels, indolent cases show greater heterogeneity, ranging from marked upregulation to little or no increase relative to healthy donor controls. Since FDXR transcription is enriched in proliferating ATL cells, likely in response to excessive replication, its upregulation in indolent patients may signal atypically high proliferative activity, thereby predicting poorer outcomes. Conversely, in aggressive ATL, where high proliferation is common, further increases in FDXR would offer limited prognostic insight. Our data further show that FDXR levels are similarly elevated in HAM/TSP and aggressive ATL. Although this supports a broader association between FDXR upregulation and HTLV-1 disease severity, the absence of matched FDXR expression and clinical outcome data currently precludes assessment of the prognostic potential of FDXR in HAM/TSP.

The broad availability of fluorouracil and bleomycin, both part of the WHO Essential Medicines List, supports their potential for repurposing. However, although in our assays ATL cells displayed increased sensitivity to fluorouracil relative to other hematologic models, their responses remain within the intermediate response range previously reported for colorectal cancer cells (Bracht et al, 2010), suggesting the need for combination treatments for optimal efficacy. In this regard, targeting anti-apoptotic BCL-2 family proteins is a promising strategy against ATL (Cooney et al, 2025; Ishitsuka et al, 2012) and may enhance the pro-apoptotic effects associated with FDXR upregulation. More broadly, FDXR upregulation can serve as a basis for targeted drug selection to overcome the well-known chemoresistance of ATL. Notably, nutlin-3, one of the highest-ranking compounds in our predictions, has been previously shown to exert selective toxicity toward wt-p53 ATL cells (Hasegawa et al, 2009).

The upregulation of FDXR in HTLV-1-infected cells is driven by p63 and p73, consistent with their ability to bind the FDXR promoter. Interestingly, our knockout experiments, along with RNA and protein expression profiles of p63 and p73, suggest that different patients and cell models predominantly rely on one of these two proteins, with the other providing only a partial contribution. Whereas enhanced fluorouracil sensitivity in colorectal cancer cells with high FDXR expression was initially reported only in the presence of wt-p53 (Hwang et al, 2001), our data indicate that in ATL cells, this can occur even when p53 is absent (e.g., in ATL-55T cells). We further show that TAp63 and TAp73 are the isoforms upregulated in infected cells, in agreement with previous reports linking HBZ to TAp73 induction (Toyoda et al, 2023). Of note, TAp63 upregulation by HTLV-1 infection had not been described previously. We find that HTLV-1-infected cells express only the TAp63 isoform, with ΔNp63 completely absent. Given the established role of p63 isoform balance in stem-cell regulation and epidermal biology (Li et al, 2023), it is intriguing to speculate that this dysregulation may be linked to the skin manifestations commonly seen in ATL.

By identifying a novel AICE motif (Glasmacher et al, 2012) bound by IRF4/BATF3 in ATL cells and overlapping an enhancer within the *TP63* locus, we show a potential joint regulation of p63 and p73 by this signaling axis, which had previously been linked only to the latter (Nakagawa et al, 2018c). IRF4 induction is a recognized hallmark of HTLV-1 infection (Kataoka et al, 2015b; Nakagawa et al, 2018c), can be driven by both Tax and HBZ (Chiang, 2020), and has been proposed as a unifying therapeutic target (Nakagawa et al, 2018c; Rauch et al, 2020). The potential link between IRF4 activation and FDXR upregulation through p63 and p73 would explain our observation that FDXR is upregulated in both ATL and HAM/TSP patients, which typically exhibit high expression of HBZ and Tax, respectively (Bangham, 2023; Bangham et al, 2015). IRF4 is also a known mutational hotspot in ATL (Kataoka et al, 2015b), which can lead to persistent activation of IRF4 independently of ongoing viral gene expression. This is in line with our observation that, at a single-cell level in ATL patients, FDXR expression significantly correlated with CADM1, but not HBZ expression. Overall, the pathway here proposed linking IRF4/p63/p73/FDXR upregulation, cellular proliferation, and elevated mitochondrial metabolism could help contextualize several independent findings in the field, such as increased ROS levels in infected cells (Takahashi et al, 2013), the high proliferative capacity of ATL cells (Bangham, 2023; Tan et al, 2021b), and metabolic dysregulation induced by HTLV-1 (Toyoda et al, 2023).

One limitation of our work is that the analyses were mostly confined to CD4 T cells. Although this population harbors the majority of the proviral load, other cell types can also be infected by HTLV-1 (Bangham, 2023), and their FDXR expression profiles would warrant investigation in future studies. Moreover, despite the long-standing clinical use of fluorouracil and bleomycin, their potential repurposing for a hematological malignancy such as ATL would necessitate dedicated in vivo studies or early-phase exploratory clinical trials. Although these compounds could represent a novel addition to ATL chemotherapy, resistance mechanisms to both fluorouracil and bleomycin have been described in various cancers (de Haas et al, 2008; Wang et al, 2004), and their likelihood in ATL remains currently unknown. Finally, although FDXR upregulation and its underlying mechanism were supported by data from multiple independent cohorts, the donors from which drug-seq samples were obtained included predominantly indolent ATL cases. Given that acute and lymphoma ATL subtypes exhibit the highest median FDXR expression, it is plausible that the efficacy of fluorouracil and bleomycin may be higher in aggressive subtypes characterized by elevated FDXR levels.

Taken together, our study reconstructs a molecular pathway converging on FDXR upregulation in HTLV-1-infected cells, revealing its potential as a prognostic marker and as a candidate determinant for chemotherapy selection in ATL.

## Methods

**Table:** Reagents and tools table

Reagent/resource	Reference or source	Identifier or catalog number
**Experimental models**		
MT-2	BEI Resources Repository	ARP-237
MT-4	BEI Resources Repository	ARP-120
OS-P2	BEI Resources Repository	ARP-1393
C10/MJ	BEI Resources Repository	ARP-4407
C8166-45	BEI Resources Repository	ARP-404
Jurkat E6.1	ECACC	88042803
CEM-ss	BEI Resources Repository	ARP-776
HuT-78	BEI Resources Repository	ARP-89
MOLT-4	BEI Resources Repository	ARP-175
Sup-T1	BEI Resources Repository	ARP-100
SP	BEI Resources Repository	ARP-3059
U937	Italian Institute of Health	CVCL_0007
MT-1	Kumamoto University	CVCL_M518
TL-Om1	Kumamoto University	CVCL_B473
ATL-55T(+)	Kumamoto University	CVCL_VQ17
ED	Kumamoto University	CVCL_JF70
HEK293-T	ECACC	12022001
ATL-55T(+) TP63-KO	This work	N.A.
ATL-55T(+) TP73-KO	This work	N.A.
ATL-55T(+) TP63-KO TP73-KO	This work	N.A.
ATL-55T(+) IRF4-KO	This work	N.A.
ATL-55T(+) BATF3-KO	This work	N.A.
MT-4 TP63-KO	This work	N.A.
MT-4 TP73-KO	This work	N.A.
MT-4 TP63-KO TP73-KO	This work	N.A.
Primary PBMCs or CD4 T cells	This work	N.A.
**Recombinant DNA**		
lentiCas9-Blast lentivirus	Addgene	#52962-LV
lentiGuide-Puro	Addgene	#52963
LentiGuide-Neo	Addgene	#139449
psPAX2	Addgene	#12260
pVSVg	Addgene	#8454
Non-targeting sgRNA lentiGuide-Puro control plasmid	Addgene	#80262
LentiTP63_1-Puro lentivirus	This work	N.A.
LentiTP63_2-Puro lentivirus	This work	N.A.
LentiTP73_1-Puro lentivirus	This work	N.A.
LentiTP73_2-Puro lentivirus	This work	N.A.
LentiTP73_1-Neo lentivirus	This work	N.A.
LentiTP73_2-Neo lentivirus	This work	N.A.
LentiIRF4_1-Puro lentivirus	This work	N.A.
LentiIRF4_2-Puro lentivirus	This work	N.A.
LentiBATF3-Puro lentivirus	This work	N.A.
**Antibodies**		
anti-p63α	Cell Signaling	#13109
anti-ΔNp63	Cell Signaling	#67825S
anti-Histone H3	Cell Signaling	#4620
IgG control	Cell Signaling	#8726
anti-FDXR	Proteintech	#15584-1-AP
anti-p53	Cell Signaling	#2527
anti-p73	Cell Signalling	#14620
anti-S. pyogenes-Cas9	Cell Signalling	#14697
anti-VDAC	Invitrogen	#MA5-33205
anti-Tax	Merck	#MABF3063
anti-HTLV-1-p24	Santa-Cruz	#sc-53891
anti-HTLV-1-gp46	Santa-Cruz	#sc-53890
anti-alpha Tubulin	GeneTex	#GTX112141
anti-beta Actin	Cell Signaling	#3700
Goat anti-rabbit IgG Alexa Fluor 488	Thermo Fisher Scientific	#A-11008
Goat anti-rabbit IgG Alexa Fluor 647	Thermo Fisher Scientific	#A-21244
IRDye® 680 Rabbit	LICORbio	#925-68021
IRDye® 680 Mouse	LICORbio	#925-68070
IRDye® 800 Rabbit	LICORbio	#925-32211
IRDye® 800 Mouse	LICORbio	#925-32210
**Oligonucleotides and other sequence-based reagents**		
qPCR primers and probes	This work	Table EV3
ddPCR primers and probes	This work	Table EV3
sgRNA sequences	This work	Table EV3
**Chemicals, enzymes and other reagents**		
IL-2	PeproTech	#200-02
PHA	Gibco	#10576015
Fluorouracil	ApexBio	#A4071
Bleomycin sulfate	Merck	#BP971
FastDigest BsmBI	Thermo Fisher Scientific	#FD0454
FastAP	Thermo Fisher Scientific	#EF0654
ibidi Mounting Medium	ibidi	#50001
µ-Slide 18 Well	ibidi	#81816
CellTiter 96® AQueous MTS Reagent Powder	Promega	#G1112
**Software**		
R	R Project/CRAN	v4.4.0
RStudio	Posit Software, PBC	v2026.01.2
Empiria Studio	LICORbio	v3.2
Fiji	Fiji, ImageJ.net	v2.16
Imaris	Oxford Instruments	v11
MetaboAnalyst	Xia Lab/MetaboAnalyst.ca	v6.0
Proteome Discoverer	Thermo Fisher Scientific	v2.4
Xcalibur	Thermo Fisher Scientific	v3.0
Compound Discoverer	Thermo Fisher Scientific	v3.3 SP3
CFX Maestro	Bio-Rad	v2.3
GraphPad Prism	GraphPad Software	v10
**Other**		
cOmplete Protease Inhibitor Cocktail	Roche	#05892970001
PhosSTOP	Roche	#04906845001
MitoTracker Red CMXRos	Invitrogen	#M46752
Click-iT™ EdU Cell Proliferation Kit	Thermo Fisher Scientific	#C10639
DAPI	Invitrogen	#D3571
SimpleChIP® Enzymatic Chromatin IP Kit	Cell Signaling Technology	#9003
AllPrep DNA/RNA Isolation Kit	Qiagen	#80204
QIAshredder	Qiagen	#79656
Mitochondria Isolation Kit	Miltenyi Biotec	#130-094-833
cell homogenizer	isobiotec	DEKAI24BRU
Ficoll-Paque PLUS	Cytiva	#17144003
mPAGE loading buffer	Merck	MPSB-10ML
EasySep™ Human CD4+ T-Cell Isolation Kit	Stemcell	#17952
RosetteSep™ Human CD4+ T-Cell Enrichment Cocktail	Stemcell	#15062
MERCURIUS™ BRB-seq Library Preparation	Alithea Genomics	#10813
iScript™ Reverse Transcription Supermix for RT-qPCR	Bio-Rad	#1708840
iQ Supermix	Bio-Rad	#1708860
ddPCR Supermix for Probes (No dUTP)	Bio-Rad	#1863023
Droplet Generation Oil for Probes	Bio-Rad	#1863005

### Ethics and clinical stratification

All primary blood samples were collected after written informed consent was obtained and in accordance with the principles set out in the WMA Declaration of Helsinki and the Department of Health and Human Services Belmont Report.

Total blood of healthy donors in the United Kingdom was obtained following approval by the Southwest—Cornwall & Plymouth Research Ethics Committee (REC 23/SW/0029).

Total blood from PLHTLV and healthy donors in Brazil was obtained following approval by the Research Ethics Committees of each reference center and by CONEP (National Commission for Research Ethics), under approval number 7.742.287. The diagnosis of HTLV-1 infection was based on the availability of previous serological screening (chemiluminescence ELISA) and confirmatory PCR or Western blot. The stratification of ATL cases followed the Shimoyama classification (Shimoyama, 1991). The diagnosis of HAM/TSP was based on clinical evaluation and laboratory testing, with spinal MRI and neurophysiological studies used to support the diagnosis and help differentiate it from other neurological conditions.

### Cell lines

HTLV-1 infected (MT-2, MT-4, OS-P2, C10/MJ, and C8166-45), the ATL cell line SP, and uninfected (Jurkat E6.1, CEM-ss, HuT-78, MOLT-4, Sup-T1, U937) cell lines were obtained through the Biodefense and Emerging Infections Research (BEI) Resources Repository (Catalog Numbers: ARP-237, ARP-120, ARP-1393, ARP-4407, ARP-404, ARP-3059, ARP-776, ARP-89, ARP-175, and ARP-100) except for U937 cells, which were a kind gift from Dr. Andrea Savarino (Italian Institute of Health) and Jurkat E6.1 cells, which were obtained from the European Collection of Authenticated Cell Cultures (ECACC Catalog No. 88042803). ATL cell lines (MT-1, TL-Om1, ATL-55T(+), ED) were a kind gift of Dr. Jun-ichirou Yasunaga (Kumamoto University). Cell lines were not independently re-authenticated in our laboratory, as they were obtained either directly from recognized cell repositories or from collaborators who had either sourced them from recognized repositories or from the laboratories in which the cell lines were originally established. Cells were periodically tested to exclude mycoplasma contamination.

Cells were cultured in RPMI medium (Merck) complemented with 10% FCS and 1% penicillin/streptomycin. The medium of SP and ATL-55T(+) cells was also complemented with 100 U/mL IL-2.

HEK293-T cells (ECACC Catalog No. 12022001) were cultured in DMEM medium complemented with 10% FCS and 1% penicillin/streptomycin. All cells were maintained at 37 °C in a 5% CO_2_ incubator.

### Primary cells

Peripheral blood mononuclear cells (PBMCs) were isolated within 4 h of blood collection using Ficoll-Paque PLUS (Cytiva). Briefly, the blood was diluted 1:1 in PBS and layered over Ficoll at a ratio of 2:1. After centrifugation at 400×*g* for 30 min, the PBMC layer was transferred to a clean tube and washed twice with PBS. Cell viability was assessed using trypan blue exclusion and quantified with an automated Countess II cell counter (Invitrogen). Immediately after isolation, PBMCs were cryopreserved and stored in liquid nitrogen until further use.

CD4 T cells were isolated either directly from total blood using the RosetteSep™ Human CD4 + T-Cell Enrichment Cocktail (Stemcell) or from frozen PBMCs using the EasySep™ Human CD4 + T-Cell Isolation Kit (Stemcell) according to the manufacturer’s instructions. Cells were cultured in RPMI medium (Merck) complemented with 10% FCS, 1% penicillin/streptomycin and 20–100 U/mL IL-2. To compare resting and activated uninfected cells, CD4 T cells were stimulated by adding phytohemagglutinin (PHA; Gibco) at a 1:100 dilution to the complete culture medium.

### TMT proteomics

HTLV-1^+^/ATL (MT-2, MT-4, and SP) and uninfected (Jurkat, CEM-ss, and MOLT-4) cell lines were lysed in RIPA buffer complemented with Protease and Phosphatase Inhibitor Cocktail (cOmplete Protease Inhibitor Cocktail and PhosSTOP, and Roche). Lysates were prepared in quadruplicate for each cell line. Lysate concentration was quantified by Pierce BCA assay (Thermo Fisher Scientific) and confirmed as >2 mg/mL for all samples.

Protein samples (100 µg each) were digested with trypsin (2.5 µg per 100 µg protein; 37 °C, overnight), labeled with TMTpro reagents according to the manufacturer’s protocol (Thermo Fisher Scientific), and pooled. An aliquot of 100 µg of the pooled sample was desalted using a SepPak cartridge according to the manufacturer’s instructions (Waters, Milford, Massachusetts, USA). Eluate from the SepPak cartridge was evaporated to dryness and resuspended in buffer A (20 mM ammonium hydroxide, pH 10) prior to fractionation by high pH reversed-phase chromatography using an Ultimate 3000 liquid chromatography system (Thermo Fisher Scientific). In brief, the sample was loaded onto an XBridge BEH C18 Column (130 Å, 3.5 µm, 2.1 mm × 150 mm, Waters, UK) in buffer A and peptides eluted with an increasing gradient of buffer B (20 mM ammonium hydroxide in acetonitrile, pH 10) from 0–95% over 60 min. The resulting fractions (concatenated into 20 in total) were evaporated to dryness and resuspended in 1% formic acid prior to analysis by nano-LC MS/MS using an Orbitrap Fusion Lumos mass spectrometer (Thermo Scientific). High pH RP fractions were further analyzed using an Ultimate 3000 nano-LC system in line with an Orbitrap Fusion Lumos mass spectrometer (Thermo Scientific). Briefly, peptides in 1% (vol/vol) formic acid were injected onto an Acclaim PepMap C18 nano-trap column (Thermo Scientific). After washing with 0.5% (vol/vol) acetonitrile 0.1% (vol/vol) formic acid peptides were resolved on a 500 mm × 75 μm Acclaim PepMap C18 reverse phase analytical column (Thermo Scientific) over a 150 min organic gradient, using seven gradient segments (1–6% solvent B over 1 min, 6–15% B over 58 min, 15–32% B over 58 min, 32–40% B over 5 min, 40–90% B over 1 min, held at 90% B for 6 min and then reduced to 1%B over 1 min) with a flow rate of 300 nl min^−1^. Solvent A was 0.1% formic acid and Solvent B was aqueous 80% acetonitrile in 0.1% formic acid. Peptides were ionized by nano-electrospray ionization at 2.0 kV using a stainless-steel emitter with an internal diameter of 30 μm (Thermo Scientific) and a capillary temperature of 300 °C. All spectra were acquired using an Orbitrap Fusion Lumos mass spectrometer controlled by Xcalibur 3.0 software (Thermo Scientific) and operated in data-dependent acquisition mode using an SPS-MS3 workflow. FTMS1 spectra were collected at a resolution of 120,000, with an automatic gain control (AGC) target of 200,000 and a max injection time of 50 ms. Precursors were filtered with an intensity threshold of 5000, according to charge state (to include charge states 2–7) and with monoisotopic peak determination set to Peptide. Previously interrogated precursors were excluded using a dynamic window (60 s ±10ppm). The MS2 precursors were isolated with a quadrupole isolation window of 0.7 m/z. ITMS2 spectra were collected with an AGC target of 10 000, max injection time of 70 ms and CID collision energy of 35%. For FTMS3 analysis, the Orbitrap was operated at 50000 resolution with an AGC target of 50,000 and a max injection time of 105 ms. Precursors were fragmented by high-energy collision dissociation (HCD) at a normalized collision energy of 60% to ensure maximal TMT reporter ion yield. Synchronous precursor selection (SPS) was enabled to include up to 10 MS2 fragment ions in the FTMS3 scan.

Raw data files were processed and quantified using Proteome Discoverer software v2.4 (Thermo Scientific) and searched against the UniProt Human database (downloaded January 2024: 82415 entries), the Uniprot HTLV-1 database (downloaded June 2024: nine entries) and the P13 sequence using the SEQUEST HT algorithm. Peptide precursor mass tolerance was set at 10 ppm, and MS/MS tolerance was set at 0.6 Da. Search criteria included oxidation of methionine (+15.995 Da), acetylation of the protein N-terminus (+42.011 Da) and methionine loss plus acetylation of the protein N-terminus (−89.03 Da) as variable modifications and carbamidomethylation of cysteine (+57.0214) and the addition of the TMTpro mass tag (+304.207) to peptide N-termini and lysine as fixed modifications. Searches were performed with full tryptic digestion, and a maximum of two missed cleavages were allowed. The reverse database search option was enabled, and all data was filtered to satisfy a false discovery rate (FDR) of 5%.

Protein grouping and total peptide-amount normalization were performed in Proteome Discoverer 2.4, while master-protein refinement and all subsequent statistical analyses were conducted in R (v4.4.0). The master-protein improvement script used queried UniProt to obtain the current annotation status for all protein accessions (updated 2024-07-17), updating redirected or obsolete entries accordingly, and then selecting the best-annotated UniProt candidate, prioritizing reviewed records. Abundance values were log2-transformed and used as input for pathway analysis with the Camera gene set algorithm in ReactomeGSA (Reactome Version 93) (Griss et al, 2020). Differential expression analysis was performed using the limma package (v3.64.3) (Ritchie et al, 2015) in RStudio. Differential expression results were filtered using the Mitocarta database (v 3.0) (Rath et al, 2021).

### Metabolomic analysis

The analysis of intracellular metabolic extracts from HTLV-1^+^/ATL (MT-2, MT-4, SP) and uninfected (Jurkat E6.1, MOLT-4, and CEM-ss) cell lines was conducted as previously described (Shytaj et al, 2021). Briefly, a cold extraction solution containing 80% methanol and 20% water was used to extract polar metabolites. Samples were analyzed by LC-MS/MS on a Thermo Scientific Q Exactive Plus mass spectrometer coupled to a Vanquish UHPLC System. Liquid chromatography separation was performed under HILIC pH 9 conditions using a ZIC-pHILIC column. Peak areas, representing metabolite levels, were extracted using Thermo Compound Discoverer 3.3 SP3. Only metabolites identified either by matching their retention time and fragmentation spectra to those of authentic pure standards or by spectral matching to compounds in the mzCloud database were retained for downstream analysis. One quality control (QC) sample pool was made by pooling a small aliquot from each sample. This pool was analyzed at the beginning, middle, and end of the sample sequence. Samples were analyzed in a pseudorandomized order, with 4 µL injected per run. Metabolite abundances were normalized against both QC samples and the total metabolite signal to account for differences in sample amount. The resulting normalized values were Log_10_ transformed and used as input for downstream analysis in MetaboAnalyst (Pang et al, 2024) (v6.0). Enrichment analysis was conducted using the dedicated module in MetaboAnalyst with the small molecule pathway database (SMPDB) metabolite set serving as reference library and including only metabolite sets containing at least two metabolites. Hierarchical clustering analysis was performed in the one-factor statistical analysis module of MetaboAnalyst. The data were row-scaled, and clustering was performed using Euclidean distance as the similarity metric and the Ward linkage method for cluster aggregation. The top twenty metabolites, ranked by *t*-test results after Benjamini–Hochberg false discovery rate (FDR) correction, were selected for visualization in the heatmap.

### Mitochondrial isolation

Mitochondria were isolated using the Mitochondria Isolation Kit, human (Miltenyi Biotec), following the manufacturer’s instructions. Briefly, 20 × 10^6^ cells were washed twice with PBS and then lysed with lysis buffer complemented with cOmplete Protease Inhibitor Cocktail (Roche). Cells were homogenized using a cell homogenizer (isobiotec) with a 10 µm clearance ball and 8–10 strokes. The homogenized lysate was then incubated with anti-TOM22 MicroBeads for 30 min at 4 °C. Mitochondria were purified by magnetic isolation using LS Columns (Miltenyi Biotec).

### Western blot

Whole cells or isolated mitochondria were lysed in RIPA buffer complemented with cOmplete Protease Inhibitor Cocktail (Roche) for 5 min at 4 °C. Protein lysates were cleared by centrifugation at 8000×*g* for 10 min and either used immediately or snap-frozen in liquid nitrogen and stored at −80 °C for subsequent use. For electrophoresis, protein lysates were mixed with mPAGE loading buffer (Merck) and β-mercaptoethanol, then incubated at 70 °C for 10 min. Samples were run on a 4–12% gradient gel at 100–150 V, and proteins were transferred onto a nitrocellulose membrane at 100 V for 1 h at RT. Membranes were blocked with PBS Blocking Buffer (LICORbio) for 1 h at RT, followed by overnight primary antibody incubation at 4 °C. The primary antibodies used included anti-FDXR (1:1000, Rabbit, Proteintech #15584-1-AP), anti-p53 (Rabbit, Cell Signaling #2527), anti-p63α (1:1000, Rabbit, Cell Signaling #13109), anti-p73 (1:1000, Rabbit, Cell Signalling #14620), anti-*S. pyogenes*-Cas9 (1:1000, Mouse, Cell Signalling #14697) anti-VDAC (1:500, Rabbit, Invitrogen #MA5-33205), anti-Tax (1:250, Mouse, Merck #MABF3063), anti-HTLV-1-p24 (1:250, Mouse, Santa-Cruz #sc-53891), anti-HTLV-1-gp46 (1:250, Mouse #sc-53890), anti-alpha Tubulin (1:15000, Rabbit, GeneTex #GTX112141), anti-beta Actin (1:15,000, Mouse, Cell Signaling #3700). After three washes in PBS 0.1% tween, membranes were incubated with secondary antibodies coupled with IRDye® 680 or 800 for 1 h RT in the dark. Membranes were imaged using an Odyssey XF imaging system (LICORbio). Quantifications were performed with the Empiria Studio software (v3.2, LICORbio).

### Immunofluorescence

Cells were seeded on poly-D-lysine–coated 18-well chamber slides (Ibidi). To visualize mitochondria, cells were stained with Mitotracker Red CMXRos Dye (1:500, Invitrogen #M46752) for 30 min at 37 °C. The Click-iT™ EdU Cell Proliferation Kit (Thermo Fisher) was used to stain proliferating primary CD4 T cells, according to the manufacturer’s instructions. To this purpose, cells were first incubated with 10 µM Edu for 2 h at 37 °C. Cells were then fixed with 4% paraformaldehyde (PFA) for 1 h at RT, followed by permeabilization with 0.1% Triton X-100 in PBS for 10 min at RT. EdU incorporation was detected using the Click-iT reaction cocktail as provided in the kit. For subsequent immunofluorescent staining, cells were blocked with 1% BSA in PBS for 30 min at RT and primary antibody incubation (anti-FDXR, 1:100, Rabbit, Proteintech #15584-1-AP) was performed in a humidified chamber at 37 °C for 1 h. Secondary antibody (Goat anti-Rabbit IgG, Alexa Fluor^TM^ 488 or 647, 1:3000 in blocking buffer) was incubated together with DAPI (1:3000) in a humidified chamber at 37 °C for 30 min. Wells were filled with Ibidi mounting medium, and samples were imaged using a Leica SP8 confocal microscope. Images were analysed with Fiji (v 2.16) and Imaris (v11, Oxford Instruments).

### Transcriptomic data reanalysis

RNA-Seq data of cell lines were retrieved from Protein Atlas. Normalized transcripts per million (nTPM) data for FDXR expression were extracted and filtered to retain only data from blood cancer (*n* = 3 HTLV^+^, 93 leukemia, 73 lymphoma, 34 myeloma) and non-cancerous (*n* = 63) cell lines.

Patient-derived microarray data were retrieved from the Gene Expression Omnibus (GEO) repository. Studies were identified using the keyword “HTLV-1” and included based on the following criteria: (1) availability of primary CD4 T cells from both PLHTLV and healthy donors; (2) use of whole-genome microarray platforms; (3) stratification of samples by disease status; and (4) inclusion of at least two donors per condition. Filtering with these criteria yielded four datasets that were used for downstream analysis: (Araya et al, 2025; Kodama et al, 2020; Pinto et al, 2014; Yamagishi et al, 2012a). Raw data were background-corrected and normalized using the normexp and quantile functions of limma (Ritchie et al, 2015). Probes were annotated to gene symbols using platform-specific annotation databases, and expression values for multiple probes mapping to the same gene were averaged. Samples corresponding to PBMCs or CD4-negative populations, as well as samples with unknown ATL subtype status, were excluded prior to integration. Batch effects between studies were corrected using the ComBat function of the sva package (Leek et al, 2012) with each GEO study being defined as a separate batch. Two-color microarray data from ATL cells subjected to IRF4 or BATF3 knockdown or treated with JQ1 (Nakagawa et al, 2018a) were processed similarly using limma, with the addition of within-array normalization (loess) followed by between-array normalization (aquantile). Normalized *M*-values were used for downstream visualization.

Bulk RNA-Seq data obtained from samples of ATL (*n* = 57), asymptomatic HTLV-1 (*n* = 3) and healthy controls (*n* = 3) were retrieved from the European Genome-phenome Archive (Kataoka et al, 2015a). One ATL sample lacked subtype annotation and was excluded from downstream analyses. Samtools (Li et al, 2009) (v1.22) was used to sort bam files and convert them to paired-end fastq files. Adapter sequences were removed and low-quality bases trimmed with fastp (Chen et al, 2018) (v0.23.4), and the resulting reads were aligned to the human reference genome (GRCh38) using STAR (v2.7.11b) (Dobin et al, 2013). Gene-level read counts were obtained with the featureCounts function (Liao et al, 2014) (v2.0.8) of the Subread suite. Differential gene-expression analysis was performed with DESeq2 (Love et al, 2014) (v1.48.2). Normalized FDXR expression values for plotting were derived by applying the variance-stabilizing transformation implemented in DESeq2.

For single-cell RNA-seq, raw sequencing data were downloaded from the Short Read Archive under study accession PRJEB47382 (Tan et al, 2021a). A custom reference transcriptome was obtained by merging human GRCh38 annotation (10x Cell Ranger pre-built reference version 2024-A) with the human T-cell leukemia virus type I genome available at GenBank (accession and version AB513134.1). For all samples, raw reads were aligned to the custom reference transcriptome, and UMI counts were calculated using the 10x Cell Ranger Count pipeline (version 9.0.0). The expression data were imported into R (version 4.1.1) and analyzed with the Seurat (Stuart et al, 2019) (version 4.3.0) and popsicleR (Grandi et al, 2022) (version 0.2.1) R packages. For each sample, low-quality cells were identified as outliers based on the number of genes, UMI counts, and the percentage of reads mapping to mitochondrial genes per cell, and these were subsequently discarded. Doublets were detected and removed using the scDblFinder (Germain et al, 2021) (version 1.8.0) R package. Cell-cycle scores were assigned to each cell using Seurat, with automated identification of cells in S or in G2/M phases of the cell cycle. Principal component analysis (PCA) was used to reduce dataset dimensionality; Louvain clustering and UMAP visualization were performed on PCA-reduced embedding after selecting an appropriate number of principal components for each sample. Upon the evaluation of an optimal clustering resolution, BlueprintEncode reference dataset from SingleR (Aran et al, 2019) (version 1.8.1) and known marker genes were used to annotate clusters as T/NK cell, B cell, myeloid, and contaminants (e.g., platelets and erythrocytes). Cells annotated as T/NK lymphocytes (94,834 cells in 17 samples) were integrated using the Seurat integration strategy (FindIntegrationAnchors and IntegrateData functions) and re-clustered with the same procedure described above. The T and NK cell clusters were classified by monitoring the expression of known markers. Low-quality and contaminated clusters were filtered out after inspecting the number of genes, UMI counts, and doublet score, resulting in ten clusters comprising 91,436 good-quality cells, of which five clusters (65,319 cells) are annotated as CD4 T cells. To assess co-expression relationships between genes at a single cell level, while accounting for high sparsity and technical artifacts, we performed an empirical, depth-stratified permutation test. For each pairwise comparison, the baseline correlation was calculated using Spearman’s rank correlation coefficient on log-normalized expression values. To construct a depth-corrected null distribution, cells were stratified into deciles based on their total UMI counts, and the expression vectors of one of the genes under investigation were permuted among cells within matching depth deciles (1000 permutations). An empirical two-tailed *P* value was calculated as the proportion of permutations yielding an absolute correlation coefficient greater than or equal to the observed baseline correlation.

### Survival and prognostic analysis

To evaluate the prognostic relevance of FDXR expression, we used the RNA-seq data from 51 patients of the dataset (Kataoka et al, 2015a) for which a clinical follow-up was available (Kataoka et al, 2015b; Kogure et al, 2022). Overall survival was censored at hematopoietic stem cell transplantation. Cox proportional-hazards models using continuous Z-score values of normalized FDXR expression were applied to the entire cohort as well as in separate analysis for the aggressive (acute and lymphoma) and indolent (chronic and smoldering) ATL subtypes. The proportional-hazards assumption was verified using Schoenfeld residuals both in the overall cohort and in each subtype. For Kaplan–Meier analyses, FDXR Z-scores were used to stratify patients into either two or four groups based on SD thresholds, and survival differences were evaluated using the log-rank test.

### In silico inference of transcription factor activity

The RNA-Seq data of all leukemia/lymphoma cell lines retrieved from Protein Atlas were stratified based on nTPM FDXR expression (mid-high FDXR group: ≥75 nTPM; mid-low FDXR group: <75 nTPM).

The discriminant regulon expression analysis (DoRothEA) package (Garcia-Alonso et al, 2019) was used to obtain high- to medium-confidence (A–C) human transcription factor (TF)–target interactions. Gene expression values (nTPM) were log2 normalized, and a context-specific regulatory network was built using the 1000 most variable genes across all RNA-Seq samples, together with all TFs. Expression values for each gene were discretized into three equal-frequency bins using the infotheo (v 1.2.0.1) package. The minet (v 3.66.0) package (Meyer et al, 2008) was used to infer regulatory interactions by first computing a mutual information matrix from discretized expression data (build.mim, estimator = “mi.empirical”) and then applying the Algorithm for the Reconstruction of Accurate Cellular Networks (ARACNe) for network pruning (ε = 0.05). To obtain the final regulon, the resulting unsigned network was overlapped with DoRothEA to assign regulatory direction (activation vs repression) where available. The signed network was formatted into a regulon object where each TF is linked to its targets with a mode of regulation (tfmode) and an associated likelihood score. The virtual inference of protein-activity by enriched regulon analysis (VIPER) algorithm (Alvarez et al, 2016), was then applied (scaling method: median absolute deviation) to the log2 normalized expression matrix to infer sample-specific TF activity scores. The statistical significance of differences in TF activity between the mid-high and mid-low FDXR groups was evaluated using the limma (v3.64.3) package (Ritchie et al, 2015), as outlined in the Statistical analysis section.

Upstream TF regulators of TP63 and TP73 were extracted from the final, signed, regulon. For each upstream TF, the regulatory weight (mor) was plotted against its differential RNA expression between the mid-high and mid-low FDXR groups, computed using the limma package. Plots were generated using the ggplot2 (v4.0.0) package in RStudio.

### Chromatin immunoprecipitation (ChIP)-qPCR

ChIP was performed using the SimpleChIP® Enzymatic Chromatin IP Kit (Cell Signaling Technology), according to the manufacturer’s instructions. Briefly, 10 × 10⁶ HTLV-1–infected (C8166-45, MT-4), ATL (SP), or uninfected (Jurkat, MOLT-4) cells were resuspended at 0.5 × 10⁶ cells/mL and cross-linked with 1% formaldehyde. After 20 min, cross-linking was quenched by the addition of 10X glycine, and cells were washed twice with ice-cold PBS. To isolate nuclei, cells were lysed sequentially in Buffer A and Buffer B supplemented with protease inhibitor cocktail and dithiothreitol (DTT). Isolated nuclei were digested using micrococcal nuclease for 20 min at 37 °C. Nuclei were pelleted and resuspended in 1X ChIP buffer containing protease inhibitor cocktail, followed by sonication (60% amplitude, three cycles of 20 s, with 30 s on ice between cycles) using a QSonica sonicator. Lysates were clarified by centrifugation, and an aliquot was used to confirm chromatin fragment size and quality by agarose gel electrophoresis. For immunoprecipitation, 5–10 µg of chromatin was incubated with anti-p63α Rabbit mAb (Cell Signaling #13109), anti-ΔNp63 (Cell Signaling #67825S), anti-p73 Rabbit mAb (Cell Signalling #14620), anti-Histone H3 Rabbit mAb (Cell Signaling #4620), or rabbit IgG control (Cell Signaling #8726) overnight at 4 °C with rotation. Immune complexes were incubated with protein G magnetic beads for 2 h at 4 °C, followed by three low-salt washes and one high-salt wash (5 min each at 4 °C). Chromatin was eluted using SDS-containing ChIP elution buffer for 30 min at 65 °C, and cross-links were reversed by incubation with NaCl and proteinase K for 2 h at 65 °C. DNA was purified using spin columns provided in the kit. qPCR was performed using primers specific for the p53 binding element in the FDXR promoter, as previously described (Liu and Chen, 2002). PCR conditions were as follows: an initial denaturation at 95 °C for 5 min, followed by 40 cycles of 95 °C for 30 s, 62 °C for 30 s, and 72 °C for 30 s, with a final extension at 72 °C for 5 min. Primer sequences are listed in Table EV3. Following amplification, 10 µL of PCR product was analyzed by 2% agarose gel electrophoresis.

### CHIP-seq data analysis

Previously published ChIP-seq datasets for IRF4, BATF3, H3K27ac, and BRD4 from ATL (Nakagawa et al, 2018b) and control cells were retrieved from ChIP-Atlas (v3.0) (Zou et al, 2024) using the following SRX identifiers: SRX2548277, SRX2548278, SRX2548279, SRX2548280, SRX2548281, SRX2548282, SRX2548284, SRX2548286, SRX2548291, SRX2548292, SRX2548294, SRX2548295, SRX2548296, SRX2548298, SRX2548299, SRX2548300, SRX1528530, and SRX398094. For each dataset, the standardized ChIP-Atlas processed files were used, which include RPM-normalized BigWig coverage tracks and MACS2 peak calls (BED4 format; hg38; *q* = 1e-05). ChIP-Atlas 3.0 processing follows a uniform pipeline using Bowtie2 (v2.2.2) for alignment, Bedtools (v2.17.0) for normalized coverage calculation, and MACS2 (v2.1.0) for peak calling. All tracks were visualized using the Integrative Genomics Viewer (Robinson et al, 2011) (IGV), together with annotation files obtained from ChIP-Atlas and IGV.

AICE (Glasmacher et al, 2012; Nakagawa et al, 2018c) motif occurrences within the TP63 genomic region and overlapping with IRF4 and BATF3 peaks in ATL cells, were detected by performing motif scanning with the Hypergeometric Optimization of Motif EnRichment [HOMER (Heinz et al, 2010), v3.12]. ChIP-seq peaks (threshold *q* = 1e-05) located within the genomic interval chr3:189,627,904–189,898,305 (hg38) were extracted in IGV and exported as a BED file. Motif analysis was conducted with the annotatePeaks.pl and findMotifsGenome.pl utilities in HOMER, using a custom motif file containing three previously described (Glasmacher et al, 2012) canonical AICE motifs (GAAATGAnTCA, TGAnTCAGAAA, and TTTCnnnnTGAnTCA). For each BATF3 or IRF4 peak, a 100 bp window centered on the peak summit was extracted and used for motif scanning after masking repetitive sequences. The motif-peak distances were plotted using the ggplot2 (v4.0.0) package in RStudio.

Changes in global BRD4 binding upon JQ1 treatment were assessed by annotating genomic coordinates in the previously described MACS2 peak files to genes using the annotatePeak function from the ChIPseeker R package (v1.46.1). Attribution of BRD4 peaks to a gene was based on a ±3 kb window from the transcription start (TSS) site using the hg38 reference genome. For genes associated with multiple BRD4 peaks, the maximum MACS2 peak score was retained. Gene-level binding scores were log2-transformed, and the change in BRD4 occupancy was quantified as the difference in log2 binding scores between JQ1-treated and DMSO-treated samples.

### RNA and DNA isolation

RNA and genomic DNA were extracted from cell lines and primary cells using the AllPrep DNA/RNA Isolation Kit (Qiagen) according to the manufacturer’s instructions. Prior to nucleic acid isolation, cell lysates were homogenized using QIAshredder columns and centrifuged for 2 min at 17,000×*g*. RNA was eluted in 30 µL of nuclease-free water, and genomic DNA was eluted in 50 µL of EB buffer.

### Gene expression quantification by qPCR

Isolated RNA was reverse transcribed using the iScript Reverse Transcription Supermix (Bio-Rad) according to the manufacturer’s instructions. Relative viral and host gene expression was analysed by duplex qPCR using the primers and probes listed in Table EV3. The TATA-box binding protein (TBP) gene was used as the housekeeping control to normalize expression levels. PCR reactions were carried out in a CFX Opus 96 machine (Bio-Rad) under the following cycling conditions: an initial denaturation at 95 °C for 30 s, followed by 49 cycles of denaturation at 95 °C for 5 s, annealing-extension at 56 °C for 30 s. Data were analysed using Bio-Rad CFX Maestro Software v2.3. For each gene of interest (GOI) and sample, the relative quantity (RQ) was calculated as: E_GOI_ ^ (Cq(min) − Cq(sample)), where E is the efficiency of the primer and probe set calculated with the formula: (% Efficiency × 0.01) + 1, Cq(min) is the average Cq for the sample with the lowest average Cq, and Cq(sample) is the average Cq value for the sample of interest. The RQ values were then used to obtain the normalized expression (ΔΔCq) levels for each GOI, calculated as: RQ(sample, GOI) ÷ RQ(sample, TBP).

### Proviral load quantification by digital droplet PCR (ddPCR)

HTLV-1 proviral load was measured by ddPCR as previously described (Katsuya et al, 2019). Primer and probe sequences are listed in Table EV3. Genomic DNA was extracted from primary CD4 T cells, and proviral load was calculated as: PVL(%) = (HTLV-1 pX copy nr)/(albumin copy nr/2) × 100.

### Gene expression knockout with CRISPR/Cas9

HTLV-1 infected (MT-4) and ATL (ATL-55T) cell lines were transduced to stably express Cas9 using the lentiCas9-Blast lentivirus (Addgene #52962-LV) (Sanjana et al, 2014) at MOI 50. Transduced cells were kept in selection with blasticidin (5 µg/mL), and the expression of Cas9 was confirmed by western blot in each experiment. The sgRNA sequences (listed in Table EV3) targeting TP63, TP73, IRF4, and BATF3 were retrieved from the Brunello library (Doench et al, 2016), and oligos were synthesized (Eurofins Genomics) with the overhangs required for cloning in the lentiGuide-Puro (Addgene #52963) (Sanjana et al, 2014) or LentiGuide-Neo (Addgene #139449) (Doyle et al, 2019) vector. Cloning was performed following the standardized protocol of the Zhang laboratory (Sanjana et al, 2014). A plasmid encoding for non-targeting sgRNA cloned in lentiGuide-Puro was used as a control (Addgene #80262). Plasmids containing cloned sgRNAs were co-transfected with the packaging plasmid psPAX2 (AddGene #12260) and the envelope plasmid pVSVg (AddGene #8454) at a ratio of 4:3:1 in HEK293-T cells. The lentiviruses obtained were used to transduce stably Cas9-expressing cells. Transduced cells were selected with puromycin (2 µg/mL) and/or geneticin (500 µg/mL), and both Cas9 expression and knockout efficiency were confirmed by Western blot.

### In silico drug efficacy prediction

The in silico screening of candidate compounds for cytotoxicity assessment was performed on data generated by the Cancer Target Discovery and Development (CTD²) Network (https://www.cancer.gov/ccg/research/functional-genomics/ctd2) established by the National Cancer Institute’s Center for Cancer Genomics. Specifically, the AUC values for individual compounds were obtained from the dataset originally described in (Seashore-Ludlow et al, 2015). The correlation between FDXR mRNA expression levels and drug efficacy was analyzed using the methodology outlined in (Rees et al, 2016) with significance thresholds determined using a Bonferroni-adjusted two-tailed z-test.

### Drug treatments

In vitro and ex vivo treatments were performed for 48–72 h, as specified in the corresponding figure legends, using fluorouracil (APExBIO) or bleomycin sulfate (Merck). All compounds were dissolved in DMSO. Corresponding serial dilutions of DMSO were used as matched controls in drug sensitivity assays.

### MTS assay

Cell viability/proliferation was assessed with the MTS assay (Promega), according to the manufacturer’s instructions. Briefly, 0.5 × 10^4^ cells were plated in triplicate into a flat-bottom 96-well plate, and 20 µL of MTS + phenazine methosulfate (PMS) solution was added to each well at the end of the drug incubation period. Cells were kept 1–3 h at 37 °C and 5% CO_2_, and the 490 nm absorbance was measured using a SPECTROstar Nano (BMG Labtech) microplate reader.

### Drug-seq

CD4 T cells of five ATL patients, three healthy donors and two ATL cell lines (SP and TL-Om1) were treated for 48 h with fluorouracil, bleomycin sulfate (5 µM or 10 µM of each drug), or DMSO control. Total RNA was isolated and used for library preparation and sequencing using a highly multiplexed 3′-end bulk RNA barcoding and sequencing approach (MERCURIUS™ BRB-seq service, Alithea Genomics SA) (Alpern et al, 2019). RNA quantity and quality were assessed using a NanoDrop spectrophotometer (Thermo Fisher Scientific, USA), and RNA concentrations were normalized to 100 ng/µL. Bulk RNA barcoding and sequencing (BRB-seq) libraries were generated using the MERCURIUS™ BRB-seq Library Preparation Kit for Illumina (Alithea Genomics, #10813) according to the manufacturer’s instructions. Libraries were sequenced on an AVITI platform (Element Biosciences). Raw sequencing reads were processed using STAR (v2.7.9a) in STARsolo mode, enabling simultaneous alignment, barcode demultiplexing, and gene quantification. Barcode demultiplexing allowed one mismatch (--soloCBmatchWLtype 1MM), and UMI deduplication was performed using the 1MM_Directional method (--soloUMIdedup 1MM_Directional). Reads were aligned to the human reference genome GRCh38, and gene-level quantification was performed using --quantMode GeneCounts, generating raw and UMI-deduplicated count matrices. UMI-deduplicated counts were used for downstream analyses to correct for PCR amplification bias. Differential expression was performed in DESeq2 (v1.50.2) using interaction designs (Condition×DrugDose or Group×DrugDose, where Condition encodes disease status and Group encodes disease status and cell type). Wald statistics were used to rank genes. Preranked GSEA was run with clusterProfiler (v4.18.4) against MSigDB Hallmark gene sets (obtained via the msigdbr package, v25.1.1), using the conventional (Subramanian et al, 2005) GSEA FDR < 0.25 threshold. Enrichment curves and ridge plots were generated with the enrichplot (v1.30.4) and ggridges (v0.5.7) packages, respectively.

### Statistical analysis

The significance of HTLV-1^+^ cell line ranking by FDXR expression among the Human Protein Atlas cell lines was assessed using an exact permutation-based test. In each possible permutation, FDXR expression values were reassigned across cell lines, and the number of HTLV-1^+^ cell lines ranked among the top five expressers was recorded. The exact *p* value was defined as the proportion of permutations yielding an enrichment equal to or greater than that observed in the original dataset.

The significance of differences in log₂ FDXR expression across disease conditions in the meta-analysis of microarray datasets was assessed using a linear mixed-effects model fitted with the lme4 (v1.1.38) package, with disease status included as a fixed effect and the study from which each dataset was derived included as a random intercept. Post hoc contrasts comparing each disease group with uninfected controls were estimated from study-adjusted marginal means using the emmeans (v2.0.1) package, with *p* values adjusted for multiple testing using the Holm method.

The statistical significance of the correlation between FDXR expression and HTLV-1 proviral load was determined using Spearman’s rank test.

Differences in relative qPCR gene expression were assessed using log2-transformed values and analyzed by unpaired Wilcoxon rank-sum test for two-group comparisons, or by Kruskal–Wallis test followed by Dunn’s post hoc test for multiple-group comparisons. Differences in relative protein expression levels were assessed following log2 transformation of the data using one-sample *t*-tests with a Benjamini–Hochberg correction, with the non-targeting sgRNA serving as the baseline for normalization. Differential TF activity between mid-high and mid-low FDXR groups was assessed using the limma package, applying empirical Bayes-moderated linear models with contrasts to obtain FDR-adjusted *p* values.

Drug inhibition curves were fitted for each cell line using a four-parameter nonlinear regression (4PL), and the fitted curves were used to compute AUCs using the MESS package (v0.6.0). Differences in AUC values between HTLV-1^+^/ATL and uninfected cell lines were assessed using an unpaired Wilcoxon rank-sum test.

The effects of fluorouracil and bleomycin on cell proliferation (EdU incorporation status) were analysed at the single-cell level using a binomial mixed-effects model fitted with the lme4 package, with treatment as a fixed effect and donor as a random intercept. Estimated marginal means were computed using the emmeans package, and Dunnett-adjusted post hoc comparisons were performed for each treatment versus the DMSO control.

Sample size was determined according to previous experience, published dataset availability and primary sample availability. No blinding and randomization were used. Inclusion criteria for selecting microarray datasets for data integration are reported in the dedicated methods section; no other inclusion/exclusion criteria were used.

## Supplementary information

Table EV1

Table EV2

Table EV3

Appendix

Peer Review File

Data Set EV1

Data Set EV2

Source data Fig. 1

Source data Fig. 2

Source data Fig. 3

Source data Fig. 4

Source data Fig. 5

Expanded View Figures

## Data Availability

Proteomics data generated in this study have been deposited in the ProteomeXchange Consortium via PRIDE (accession number PXD073455). Drug-seq data have been deposited in GEO (accession number GSE319793). The source data of this paper are collected in the following database record: biostudies:S-SCDT-10_1038-S44321-026-00535-0.
