# Supplementary material for: FDXR upregulation by p63/p73 is a prognostic and therapeutic marker of adult T-cell leukemia/lymphoma
Source: EMBO Mol Med. 2026 Oct 12;18(11):4529–57. doi: 10.1038/s44321-026-00535-0 (PMC13748658; doi:10.1038/s44321-026-00535-0)
Supplement: Supplementary file 1 [file 44321_2026_535_MOESM13_ESM.pdf]

Expanded View Figures

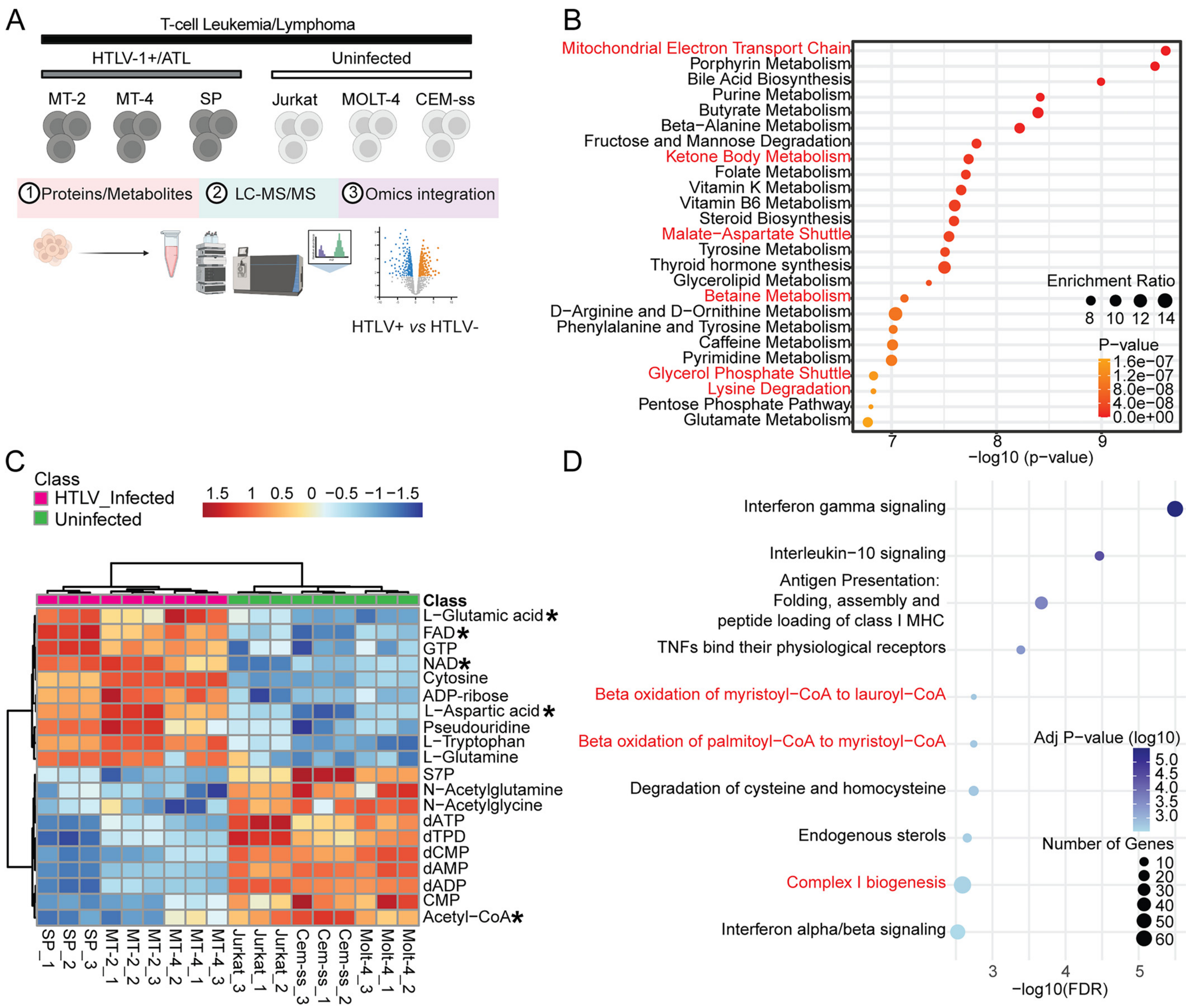

Figure EV1. Mitochondrial metabolic alterations in HTLV-1⁺/ATL cell lines.

(**A**) Experimental design. The metabolome and proteome of three HTLV-1⁺/ATL cell lines (MT-2, MT-4, SP) were compared with three uninfected lymphoid cell lines (Jurkat, MOLT-4, and CEM-ss). Cell lysates were analysed in triplicate for metabolomics and in quadruplicate for proteomics. (**B**) Pathway enrichment analysis of metabolomic profiles comparing HTLV-1⁺ and uninfected cells. The analysis was performed on SMPDB metabolite sets using the GlobalTest score test implemented in MetaboAnalyst. (**C**) Heatmap of the twenty most significantly altered metabolites in HTLV-1⁺ versus uninfected cells, ranked by *t*-test followed by Benjamini–Hochberg FDR correction. (**D**) Proteomic pathway analysis showing the ten most upregulated pathways in HTLV-1–infected cells. The analysis was performed on Reactome gene sets using a two-sided CAMERA gene set test followed by Benjamini–Hochberg FDR correction. Pathways that are exclusively or predominantly mitochondrial in panels (**B**, **D**) are highlighted in red, and metabolites from these pathways that appear in the heatmap (**C**) are indicated by asterisks.

 © The Author(s)

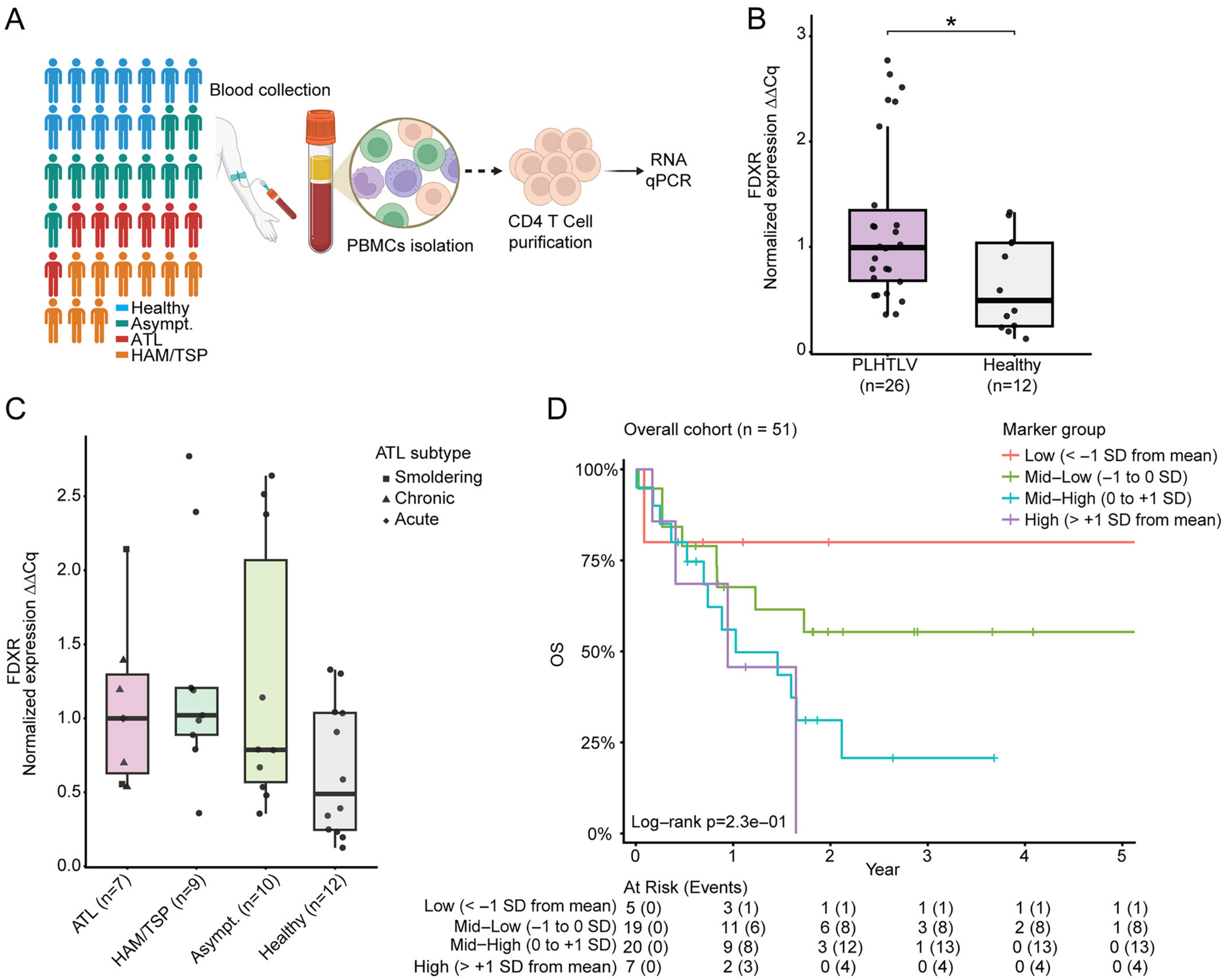

Figure EV2. FDXR mRNA levels in PLHTLV and ATL survival curves by quartiles of FDXR expression.

(**A**) CD4 T cells were isolated from PBMCs obtained from the cohort described in Table EV1. (**B**, **C**) Relative FDXR mRNA levels were calculated by ΔΔCq using TBP expression as a reference. Each dot represents an individual donor, while box plots show median ± IQR for each subgroup. In panel (**B**), differences in relative FDXR expression between PLHTLV and healthy controls were analyzed by an unpaired Wilcoxon rank-sum test following $\log_2$ transformation ($p = 0.032$). (**D**) Kaplan-Meier analysis of overall survival in patients grouped into quartiles based on the median Z-score of FDXR expression. FDXR expression values were derived from (Data ref: Kataoka et al, 2015a) and analysed in ATL patients with available survival data [$n = 51$ (Kataoka et al, 2015b; Kogure et al, 2022)]. Overall survival was censored at hematopoietic stem cell transplantation, and survival differences were assessed using the log-rank test. *$p < 0.05$.

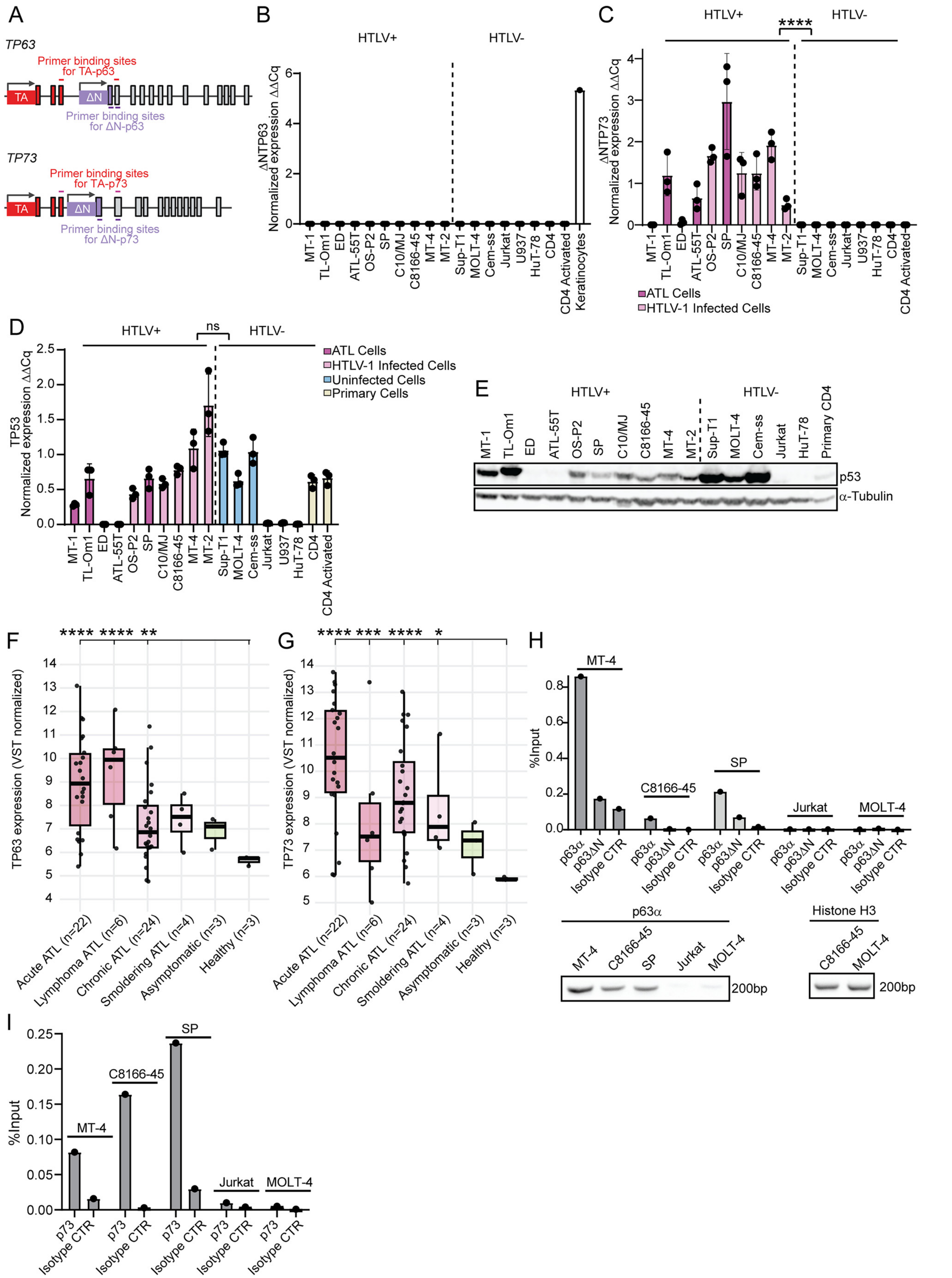
A
TP63
Primer binding sites for TA-p63
TA
ΔN
Primer binding sites for ΔN-p63
TP73
Primer binding sites for TA-p73
Primer binding sites for ΔN-p73
B
HTLV+
HTLV-
ΔNTP63 Normalized expression ΔΔCq
MT-1
TL-Om1
ED
ATL-55T
OS-P2
SP
C10/MJ
C8166-45
MT-4
MT-2
Sup-T1
MOLT-4
Cem-ss
Jurkat
U937
HuT-78
CD4
CD4 Activated
Keratinocytes
C
ΔNTP73 Normalized expression ΔΔCq
****
ATL Cells
HTLV-1 Infected Cells
D
TP53 Normalized expression ΔΔCq
ns
ATL Cells
HTLV-1 Infected Cells
Uninfected Cells
Primary Cells
E
Primary CD4
p53
α-Tubulin
F
TP63 expression (VST normalized)
**** **** **
Acute ATL (n=22)
Lymphoma ATL (n=6)
Chronic ATL (n=24)
Smoldering ATL (n=4)
Asymptomatic (n=3)
Healthy (n=3)
G
TP73 expression (VST normalized)
**** *** **** *
H
%Input
p63α
p63ΔN
Isotype CTR
Histone H3
200bp
I
p73

Figure EV3. p53 family expression profiles and chromatin binding at the FDXR promoter in HTLV-1$^+$/ATL and uninfected cells.

(**A**) Isoform-specific primer design for amplification of TA and ΔN isoforms of TP63 and TP73 by qPCR. (**B**, **C**) Relative mRNA expression of ΔN isoforms of TP63 (**B**) and TP73 (**C**) in HTLV-1$^+$, ATL, and uninfected cell lines and primary cells, as assessed by qPCR. In (**B**), primary keratinocytes were used as a positive control for ΔNTP63 expression. (**D**, **E**) Relative mRNA (**D**) and protein (**E**) expression of p53 in HTLV-1$^+$, ATL, and uninfected cell lines and primary cells, as assessed by qPCR and western blotting, respectively. In panels (**B–D**), each dot represents an independent qPCR assay ($n = 3$), and data were presented as mean ± SD. Relative mRNA levels were calculated by ΔΔCq using TBP expression as a reference. Data were analyzed by the nonparametric Wilcoxon rank-sum test ($p = 4.57e\text{-}05$ in (**C**) and $p = 0.94$ in (**D**). (**F**, **G**) TP63 and TP73 RNA expression in tumor and CD4 T cells isolated from ATL (Acute $n = 22$; Chronic $n = 24$; Lymphoma $n = 6$; Smoldering $n = 4$), asymptomatic HTLV-1 carriers ($n = 3$), and healthy controls ($n = 3$). RNA-Seq data (Data ref: Kataoka et al, 2015a) are plotted for each donor as variance-stabilized (VST) expression, while box plots show median ± IQR for each subgroup. Differential expression between each subgroup and healthy controls was tested using DESeq2. In (**F**): Acute ATL $p = 1.23e\text{-}05$; Lymphoma ATL $p = 9.59e\text{-}05$; Chronic ATL $p = 0.008$; Smoldering ATL $p = 0.173$; Asymptomatic $p = 0.551$. In (**G**): Acute ATL $p = 3.75e\text{-}07$; Lymphoma ATL $p = 1.4e\text{-}04$; Chronic ATL $p = 9.06e\text{-}05$; Smoldering ATL $p = 0.015$; Asymptomatic $p = 0.564$. (**H**, **I**) ChIP–qPCR enrichment over input showing p63 (α isoform) (**H**) and p73 (**I**) binding to the p53-responsive element (Liu and Chen, 2002) within the FDXR promoter in HTLV-1$^+$ cells and uninfected controls. Chromatin immunoprecipitated with anti-ΔNp63 or IgG antibodies was used as a control. Amplification products were analyzed by agarose gel electrophoresis, as shown in the lower panel (**H**). Primer sequences used to amplify the p53-responsive element are listed in Table EV3. *$p < 0.05$, **$p < 0.01$, ***$p < 0.001$, ****$p < 0.0001$.

© The Author(s)

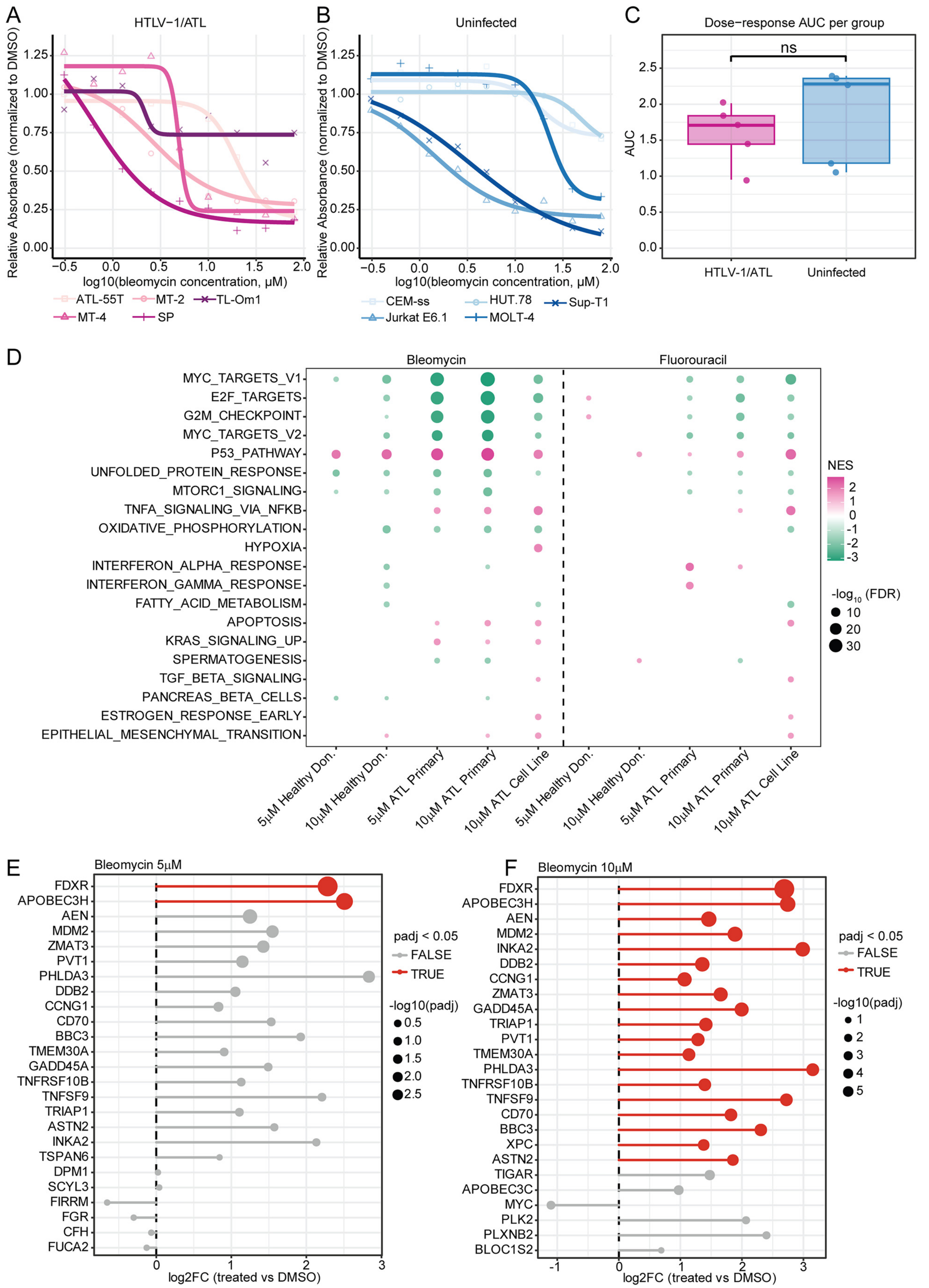
A
HTLV−1/ATL
Relative Absorbance (normalized to DMSO)
log10(bleomycin concentration, μM)
ATL-55T
MT-2
TL-Om1
MT-4
SP
B
Uninfected
CEM-ss
HUT.78
Sup-T1
Jurkat E6.1
MOLT-4
C
Dose−response AUC per group
ns
AUC
HTLV-1/ATL
Uninfected
D
Bleomycin
Fluorouracil
MYC_TARGETS_V1
E2F_TARGETS
G2M_CHECKPOINT
MYC_TARGETS_V2
P53_PATHWAY
UNFOLDED_PROTEIN_RESPONSE
MTORC1_SIGNALING
TNFA_SIGNALING_VIA_NFKB
OXIDATIVE_PHOSPHORYLATION
HYPOXIA
INTERFERON_ALPHA_RESPONSE
INTERFERON_GAMMA_RESPONSE
FATTY_ACID_METABOLISM
APOPTOSIS
KRAS_SIGNALING_UP
SPERMATOGENESIS
TGF_BETA_SIGNALING
PANCREAS_BETA_CELLS
ESTROGEN_RESPONSE_EARLY
EPITHELIAL_MESENCHYMAL_TRANSITION
NES
-log10 (FDR)
5μM Healthy Don.
10μM Healthy Don.
5μM ATL Primary
10μM ATL Primary
10μM ATL Cell Line
E
Bleomycin 5μM
FDXR
APOBEC3H
AEN
MDM2
ZMAT3
PVT1
PHLDA3
DDB2
CCNG1
CD70
BBC3
TMEM30A
GADD45A
TNFRSF10B
TNFSF9
TRIAP1
ASTN2
INKA2
TSPAN6
DPM1
SCYL3
FIRRM
FGR
CFH
FUCA2
padj < 0.05
FALSE
TRUE
-log10(padj)
log2FC (treated vs DMSO)
F
Bleomycin 10μM
FDXR
APOBEC3H
AEN
MDM2
INKA2
DDB2
CCNG1
ZMAT3
GADD45A
TRIAP1
PVT1
TMEM30A
PHLDA3
TNFRSF10B
TNFSF9
CD70
BBC3
XPC
ASTN2
TIGAR
APOBEC3C
MYC
PLK2
PLXNB2
BLOC1S2
padj < 0.05
FALSE
TRUE
-log10(padj)
log2FC (treated vs DMSO)

© The Author(s)

Figure EV4. Dose-dependent effects of bleomycin and fluorouracil in vitro and ex vivo.

(**A–C**) HTLV-1$^+$/ATL and uninfected cell lines were treated with serial dilutions of bleomycin, and cell viability/proliferation was assessed by MTS assay 72 h post-treatment. Cell models were tested in two independent experiments, each performed in technical triplicate. Mean absorbance values were normalized to DMSO controls, and dose-response curves were fitted for each cell line using four-parameter logistic nonlinear regression (**A**, **B**). The fitted curves were used to calculate AUCs (**C**). In panel (**C**), each dot represents a cell line, and data were depicted as median ± IQR. Group-wise AUC comparisons were conducted using an unpaired Wilcoxon rank-sum test. (**D–F**) Primary CD4 T cells from five ATL patients (one acute, three chronic, one smoldering) and three healthy donors, along with two ATL cell lines (SP and TL-Om1), were treated with fluorouracil or bleomycin for 48 h. RNA was extracted and analyzed by Drug-Seq. Panel (**D**) shows treatment-induced pathway changes evaluated by gene set enrichment analysis (GSEA) (Subramanian et al, 2005) using Hallmark gene sets, based on DESeq2 Wald statistics comparing drug-treated samples with DMSO controls. Bubble size indicates enrichment significance ($-\log_{10}$ FDR), and color indicates normalized enrichment score (NES). The top 20 pathways (top 10 per drug by maximum |NES|) are shown for each condition. Panels (**E**, **F**) show lollipop plots of the top 25 genes altered by bleomycin treatment at 5 µM (**E**) and 10 µM (**F**) in primary ATL cells, based on DESeq2 analysis. Genes are ranked by statistical significance (adjusted *p* value). For each gene, the stem and point position indicate the $\log_2$ fold change relative to DMSO. Point size reflects $-\log_{10}$ (adjusted *p* value), and point color denotes significance (adjusted $p < 0.05$).

© The Author(s)
