# Supplementary material for: FDXR upregulation by p63/p73 is a prognostic and therapeutic marker of adult T-cell leukemia/lymphoma
Source: EMBO Mol Med. 2026 Oct 12;18(11):4529–57. doi: 10.1038/s44321-026-00535-0 (PMC13748658; doi:10.1038/s44321-026-00535-0)
Supplement: Supplementary file 2 [file 44321_2026_535_MOESM4_ESM.pdf]

Appendix to: FDXR Upregulation by p63/p73 is a Prognostic and Therapeutic Marker of Adult T-cell Leukemia/Lymphoma

Appendix Figure S1…………………………………………………………………**2**
Appendix Figure S2…………………………………………………………………**3**
Appendix Figure S3……………………………………………………………… **4-5**
Appendix Figure S4…………………………………………………………………**6**
Appendix Figure S5…………………………………………………………………**7**

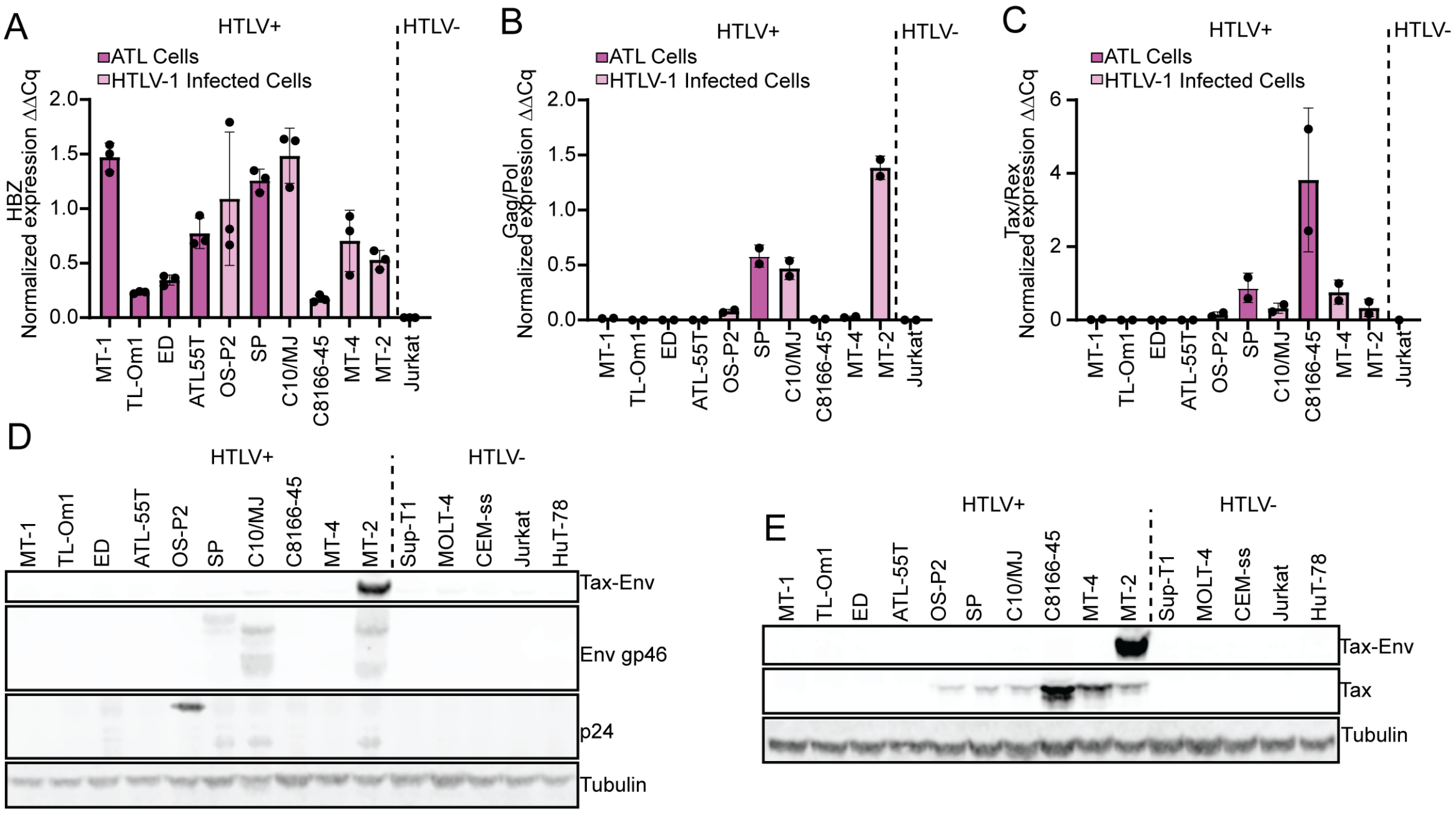

Appendix Figure S1. Related to Figure 1. Relative HTLV-1 RNA and protein levels in the cell models used in the study.

(A-C) Relative expression of the HTLV-1 transcripts Hbz (A), Gag/Pol (B) and Tax/Rex (C) were measured by qPCR using the primers listed in Table EV5. Each dot represents an independent qPCR assay, and data are presented as mean ± SD. Relative RNA levels were calculated by ΔΔCq using TBP expression as reference.

(D-E) Protein levels of HTLV-1 Env gp46 and p24 (D) and Tax (E) were assessed by western blot. The fusion protein Tax-Env, typical of the MT-2 cell line, is visible in both (D), as detected with an anti-gp46 antibody, and in (E) as detected with an anti-Tax antibody.

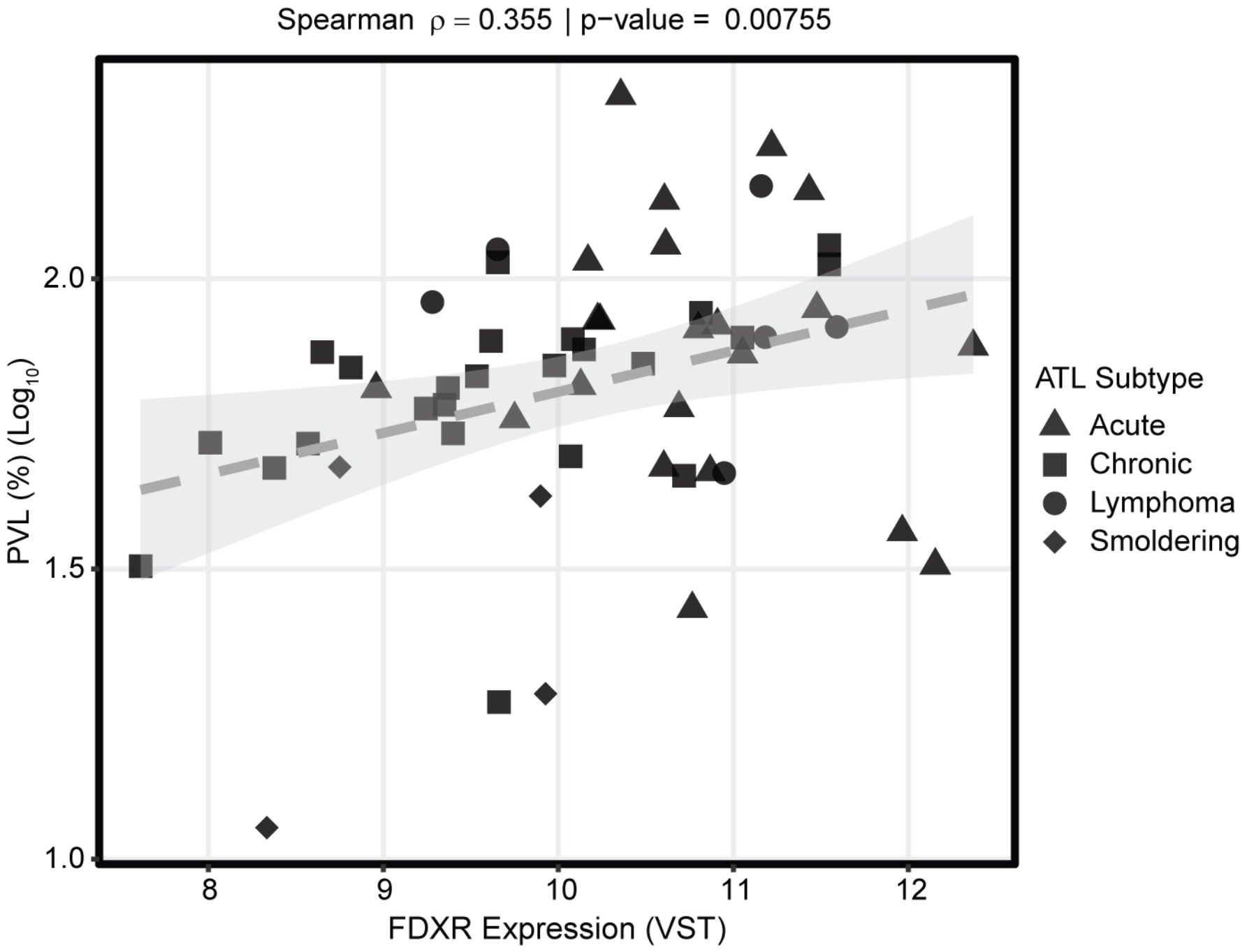

Appendix Figure S2. Related to Figure 1. Correlation between FDXR expression and proviral load in ATL patients. RNA-Seq data and HTLV-1 proviral load measurements in ATL patients were derived from (Data ref: Kataoka et al., 2015a, Kataoka *et al.*, 2015b). Each point represents an individual patient, plotting variance-stabilized FDXR expression (x-axis) against Log10 proviral load (y-axis). Point shapes indicate ATL subtypes while the dashed line shows the linear regression fit and 95% confidence interval (grey shading). The correlation was calculated using the Spearman method.

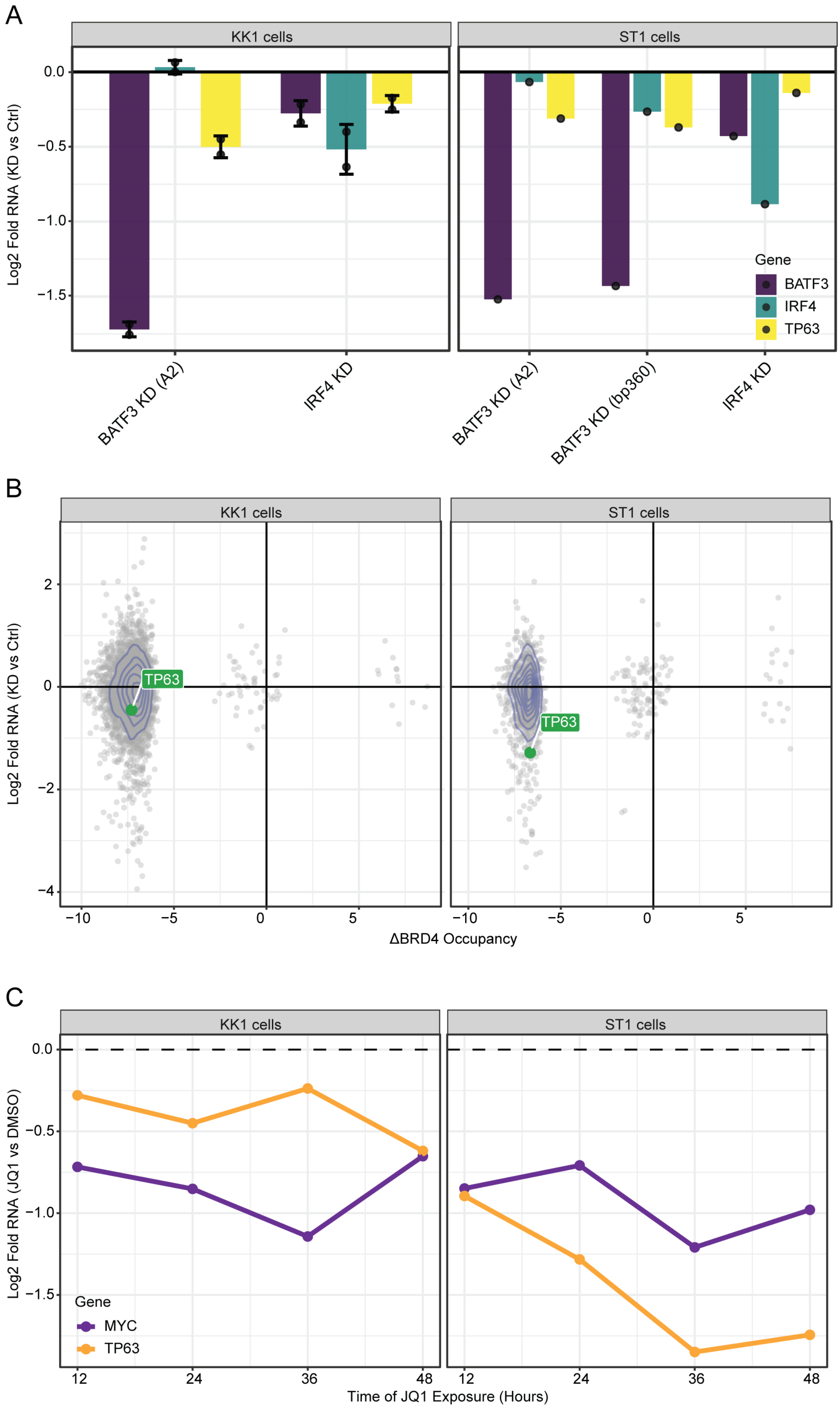
A
KK1 cells
ST1 cells
Log2 Fold RNA (KD vs Ctrl)
0.0
−0.5
−1.0
−1.5
BATF3 KD (A2)
IRF4 KD
BATF3 KD (A2)
BATF3 KD (bp360)
IRF4 KD
Gene
BATF3
IRF4
TP63
B
KK1 cells
ST1 cells
Log2 Fold RNA (KD vs Ctrl)
TP63
TP63
2
0
−2
−4
−10
−5
0
5
ΔBRD4 Occupancy
C
KK1 cells
ST1 cells
Log2 Fold RNA (JQ1 vs DMSO)
0.0
−0.5
−1.0
−1.5
Gene
MYC
TP63
12
24
36
48
Time of JQ1 Exposure (Hours)

Appendix Figure S3. Related to Figure 3. Impact of IRF4/BATF3 depletion and pharmacological BRD4 displacement on TP63 expression.

Transcriptomic and CHIP-Seq data from ATL cell lines (KK1 and ST1) following IRF4 and BATF3 shRNA knockdown or JQ1 treatment were derived from (Data ref: Nakagawa *et al.*, 2018a) and (Data ref: Nakagawa *et al.*, 2018b), respectively.

(A) Effect of IRF4 and BATF3 knockdown on TP63 RNA expression levels.

(B) Scatter plot of BRD4 displacement (±3 kb of each TSS) and corresponding changes in gene expression (Log2 Fold Change; y-axis) across all BRD4-bound genes. BRD4 displacement (x-axis) was calculated as the difference in Log2 MACS2 peak scores (JQ1 versus DMSO). The *TP63* gene is highlighted in green.

(C) Transcriptional kinetics of TP63 over a 48-hour time course of JQ1 exposure. The known BRD4-responsive control gene, MYC, is displayed for comparison.

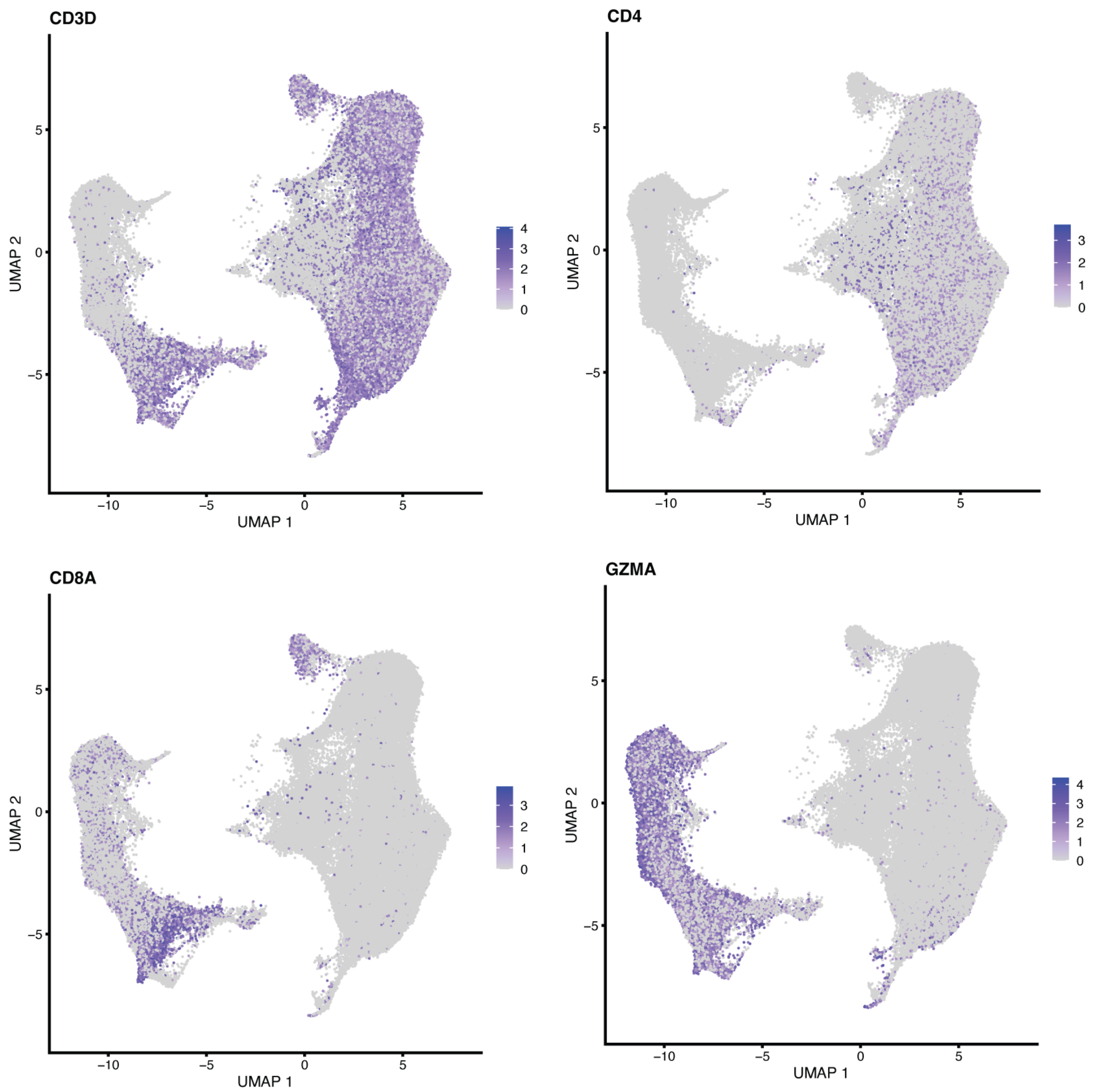

Appendix Figure S4. Related to Figure 4. Canonical T and NK lineage marker expression in integrated T/NK scRNA-seq clusters.

Raw sequencing data from PBMCs of healthy donors and PLHTLV were retrieved from (Data ref: Tan *et al.*, 2021a) After initial clustering and lineage annotation, cells identified as T/NK lymphocytes were integrated across samples and re-clustered using PCA-based dimensionality reduction followed by Louvain clustering. The UMAP feature plots show the expression of canonical T cell (CD3D, CD4, CD8A) and NK cell (GZMA) marker genes across clusters.

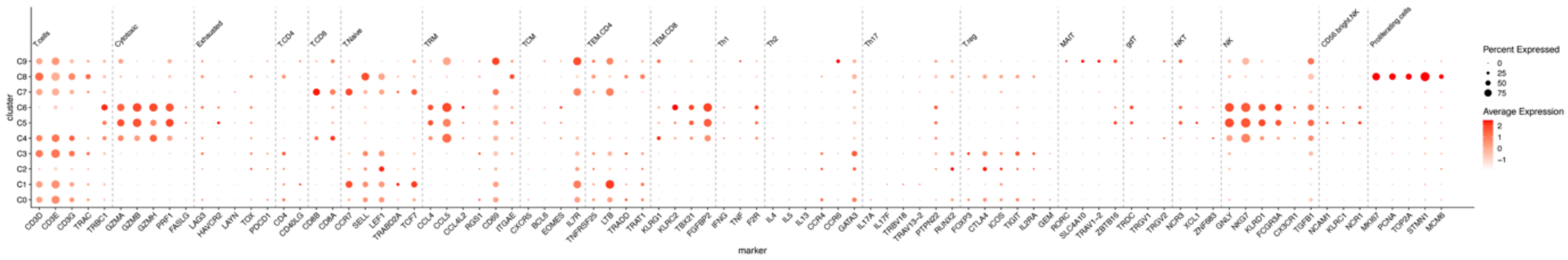

Appendix Figure S5. Related to Figure 4. Expression of marker genes used to define T/NK subset identities in scRNA-seq clusters.

Raw sequencing data from PBMCs of healthy donors and PLHTLV were retrieved from (Data ref: Tan *et al.*, 2021a). Integrated clustering of T/NK cells was performed as shown in Appendix Fig. S4. Cluster identities were defined based on the expression of canonical lineage and functional marker genes. The dot plot displays the average expression and percentage of expressing cells for markers characterizing naïve, memory, cytotoxic, exhausted, regulatory, NK, and proliferating cell subpopulations across clusters.
