# Supplementary material for: FDXR upregulation by p63/p73 is a prognostic and therapeutic marker of adult T-cell leukemia/lymphoma
Source: EMBO Mol Med. 2026 Oct 12;18(11):4529–57. doi: 10.1038/s44321-026-00535-0 (PMC13748658; doi:10.1038/s44321-026-00535-0)
Supplement: Supplementary file 3 [file 44321_2026_535_MOESM5_ESM.pdf]

EMBO Molecular Medicine

FDXR Upregulation by p63/p73 is a Prognostic and Therapeutic Marker of Adult Tcell Leukemia/Lymphoma

Lara Gallucci, Maria Josephine, Yasunori Kogure, Alex Rosini Da Silva, Vincent Cantaloube-Ferrieu, Marcos Eduardo Souza Abreu, Trusha Ramteke, Phoebe Kibble, Guilherme Lucchesi Angelo Braz, Rubens de Assis Santos Sebastião, Luiza Amoroso Lambaz, Aijun Lin, Beth Hoare, Felipe Custódio Carvalho dos Santos, Sophie Shackley, Alex Quin, Sofia Luari Moelas Giuzio, Philip Lewis, Wujuan Zhang, Youko Nukui, Juliana Pereira, Hebert Fabricio Culler, Takuro Kameda, Kazuya Shimoda, Kate Heesom, Aaron Goldman, Ester Cerdeira Sabino, Seishi Ogawa, Fabio Eudes Leal, Mattia Forcato, Keisuke Kataoka, Sheila de Oliveira Garcia Mateos, and Iart Luca Shytaj

Corresponding author(s): Iart Luca Shytaj (luca.shytaj@bristol.ac.uk)

Review Timeline:

Submission Date:	15th May 26
Editorial Decision:	19th Jun 26
Revision Received:	28th Jul 26
Editorial Decision:	7th Sep 26
Revision Received:	16th Sep 26
Accepted:	22nd Sep 26

Editor: Lise Roth

Transaction Report:

(Note: With the exception of the correction of typographical or spelling errors that could be a source of ambiguity, letters and reports are not edited. Depending on transfer agreements, referee reports obtained elsewhere may or may not be included in this compilation. Referee reports are anonymous unless the Referee chooses to sign their reports.)

19th Jun 2026

Dear Dr. Shytaj,

Thank you for submitting your manuscript to EMBO Molecular Medicine. We apologize for the time taken to reach a decision while awaiting the final referee report. Unfortunately, this report has not been received. To avoid further delays, and because the reports received provide a sufficiently comprehensive assessment of the manuscript, we have reached a decision based on the reviews currently available.

As you will see from the comments below, the reviewers recognize the novelty and potential interest of the study, while also raising several significant concerns. Referee #1 notes substantial shortcomings in the presentation of the data and the clarity of the manuscript, whereas Referee #2 identifies a number of additional analyses that would be required to strengthen the conclusions.

If you feel you can address these concerns, we would be pleased to consider a revised version of the manuscript. Please attach a cover letter detailing how you addressed each point raised by the reviewers. The revised manuscript will be subject to review again, and we cannot guarantee that the outcome will be favorable at this stage. EMBO Molecular Medicine encourages a single round of revisions, so acceptance or rejection of the manuscript depends on the completeness of your responses in the final version. For this reason, and to save you from frustration, I strongly advise against submitting an incomplete revision.

If you would like to discuss further the points raised by the referees, I am available to do so via email or video. Let me know if you are interested in this option.

When preparing your revised manuscript, please consult our revised submission guidelines:
https://link.springer.com/journal/44321/submission-guidelines#cms-Revised-submissions

Please note that all revised manuscripts undergo an initial quality-control assessment prior to being sent for re-review. Failure to provide the required documentation and information may delay the evaluation of your revision.

We require:

1) A .docx formatted version of the manuscript text (including legends for main figures, EV figures and tables). Please make sure that the changes are highlighted to be clearly visible.

2) Individual production quality figure files as .eps, .tif, .jpg (one file per figure). For guidance, download the 'Figure Guide PDF':
https://media.springernature.com/original/springer-cms/rest/v1/content/27825798/data/v1.

3) A .docx formatted letter INCLUDING the reviewers' reports and your detailed point-by-point responses to their comments. As part of the EMBO Press transparent editorial process, the point-by-point response is part of the Review Process File (RPF), which will be published alongside your paper.

4) A complete author checklist, which you can download from our author guidelines. Please insert information in the checklist that is also reflected in the manuscript. The completed author checklist will also be part of the RPF.

5) Please note that all corresponding authors are required to supply an ORCID ID for their name upon submission of a revised manuscript.

6) It is mandatory to include a 'Data Availability' section after the Materials and Methods. Before submitting your revision, primary datasets produced in this study need to be deposited in an appropriate public database, and the accession numbers and database listed under 'Data Availability'. Please remember to provide a reviewer password if the datasets are not yet public.

In case you have no data that requires deposition in a public database, please state so in this section. Note that the Data Availability Section is restricted to new primary data that are part of this study.

7) For data quantification: please specify the name of the statistical test used to generate error bars and P values, the number (n) of independent experiments (specify technical or biological replicates) underlying each data point and the test used to calculate p-values in each figure legend. The figure legends should contain a basic description of n, P and the test applied. Graphs must include a description of the bars and the error bars (s.d., s.e.m.).

8) At EMBO Press we ask authors to provide source data for the main manuscript figures. You will receive a separate email with instructions for providing source data with your revised manuscript, including how to upload and organize the files.

9) Our journal encourages inclusion of *data citations in the reference list* to directly cite datasets that were re-used and obtained from public databases. Data citations in the article text are distinct from normal bibliographical citations and should directly link to the database records from which the data can be accessed. In the main text, data citations are formatted as follows: "Data ref: Smith et al, 2001" or "Data ref: NCBI Sequence Read Archive PRJNA342805, 2017". In the Reference list, data citations must be labeled with "[DATASET]". A data reference must provide the database name, accession number/identifiers and a resolvable link to the landing page from which the data can be accessed at the end of the reference.

10) We replaced Supplementary Information with Expanded View (EV) Figures and Tables that are collapsible/expandable online. A maximum of 5 EV Figures can be typeset. EV Figures should be cited as 'Figure EV1, Figure EV2" etc... in the text and their respective legends should be included in the main text after the legends of regular figures.

- For the figures that you do NOT wish to display as Expanded View figures, they should be bundled together with their legends in a single PDF file called *Appendix*, which should start with a short Table of Content. Appendix figures should be referred to in the main text as: "Appendix Figure S1, Appendix Figure S2" etc.

- Additional Tables/Datasets should be labeled and referred to as Table EV1, Dataset EV1, etc. Legends have to be provided in a separate tab in case of .xls files. Alternatively, the legend can be supplied as a separate text file (README) and zipped together with the Table/Dataset file.

11) The paper explained: EMBO Molecular Medicine articles are accompanied by a summary of the articles to emphasize the major findings in the paper and their medical implications for the non-specialist reader. Please provide a draft summary of your article highlighting
- the medical issue you are addressing,
- the results obtained and
- their clinical impact.

This may be edited to ensure that readers understand the significance and context of the research. Please refer to any of our published articles for an example.

12) Author contributions: You will be asked to provide CRediT (Contributor Role Taxonomy) terms in the submission system. These replace a narrative author contribution section in the manuscript.

13) A "Disclosure and Competing Interests Statement" should be provided in the main text.

14) Every published paper includes a 'Synopsis' to further enhance discoverability. Synopses are displayed on the journal webpage and are freely accessible to all readers. They include a short stand first (maximum of 300 characters, including space) as well as 2-5 one-sentences bullet points that summarizes the paper. Please write the bullet points to summarize the key NEW findings. They should be designed to be complementary to the abstract - i.e. not repeat the same text. We encourage inclusion of key acronyms and quantitative information (maximum of 30 words / bullet point). Please use the passive voice. Please attach these in a separate file or send them by email, we will incorporate them accordingly.

Please also suggest a striking image or visual abstract to illustrate your article as a PNG file 550 px wide x 300 px high.

15) Include a Reagents and Tools Table as part of the Methods section, which can be downloaded from our author guidelines.

16) As part of the EMBO Publications transparent editorial process initiative (see our Editorial at http://embomolmed.embopress.org/content/2/9/329), EMBO Molecular Medicine will publish online a Review Process File (RPF) to accompany accepted manuscripts.
In the event of acceptance, this file will be published in conjunction with your paper and will include the anonymous referee reports, your point-by-point response and all pertinent correspondence relating to the manuscript. Let us know whether you agree with the publication of the RPF and as here, if you want to remove or not any figures from it prior to publication.
Please note that the Authors checklist will be published at the end of the RPF.

EMBO Molecular Medicine has a "scooping protection" policy, whereby similar findings that are published by others during review or revision are not a criterion for rejection. Should you decide to submit a revised version, I do ask that you get in touch after three months if you have not completed it, to update us on the status.

I look forward to receiving your revised manuscript.

Yours sincerely,

Lise Roth

Lise Roth, PhD
Senior Editor
EMBO Molecular Medicine

***** Reviewer's comments *****

Referee #1 (Comments on Novelty/Model System for Author):

The article presents novely aspects but ii si not well organized therefore this explains my personal choices of Medium technical quality and Medium medical impact

Referee #1 (Remarks for Author):

Gallucci and coll have undertaking a study to demonstrate that FDXR upregulation could be a prognostic and therapeutic marker in HTLV-1 positive ATL.

Major points:
Title: In the title the authors underline that ATL is HTLV-1 associated, but since the abbrevation ATL refers only to adult T cell leukemia caused by HTLV-1 I suggest to use simply ATL.
Results:
The experimental section shown in the paper demonstrates with different techniques that FDXR gene is expressed at higher level in HTLV-1 infected cells either chronically infected and/or established from HTLV-1 positive patient in comparison with uninfected cell lines. In addition a mechanism involving RF-4/p63/p73/FDXR pathway has been hypothesized.This is the most complete, organized and understandable part of the paper.
Conversely the section illustrating the data ex vivo or meta-data regarding prognostic expression of FDXR are rather confusing. What is the relationship between the meta-analysis and the what the authors define as "our cohort of blood donors in Brazil" The analyzed cohort from Brazil (51 patients) is unclear in the description and conclusions. The patient samples refer to either ATL and TSP patients only from Brazil?. While it is clear that FDXR expression is higher in PLHTLV positive individual in comparison with healthy donors, the difference in FDXR expression in ATL versus TSP in the Brazilian blood donors cohort is not described/ commented although the TSP CD4 cells express FDXR. The authors sustain that the FDXR marker is prognostic and therapeutic in ATL but in the Brazilian cohort of 51 individuals the difference between FDXR expression in ATL versus TSP is not significant. What the authors want to demonstrate by analyzing the cohort. From the RNA-seq analysis the authors conclude that FDXR expression levels in PLHTLV correlate with disease progression and worse ATL prognosis, but the number of ATL is only 7 out of 43 samples. Therefore the authors should be more punctual in describing and motivating their ex vivo and meta-analysis studies.

FDXR transcription is enriched within the proliferating cell cluster of ATL. The authors do not precisely describe the origin of ATL cohort /NK clusters that they have analyzed.Are the same cohort of Brazilian donors or the data derive from meta-analysis?
FDXR expression levels predict sensitivity to selected chemotherapeutics in vitro and ex vivo
In this case the prediction is based on profiles from the Cancer Therapeutics Response Portal (CTRP) Similarly the authors include in parenthesis without further description of the ATL cells that have been treated with bleomycin and fluorouracil in vitro. Is this the same Brazilian cohort ?
In general there is a remarkable confusion in combining the description of the results obtained in vitro, ex vivo, and through meta-analysis.

Discussion

The authors should comment why apparently the expression of FDXR is unrelated to the expression especially of regulatory genes in HTLV-1 infected cells. If the FDXR expression could be prognostic and therapeutic it should be influenced by the expression of regulatory genes as matter of fact that expression of TAX/HBZ change in vivo during the progression of the diseases. In addition although apparently bleomycin and fluorouracil have not been included in the therapy of ATL, the authors deserve to consider that the use of these drugs can give rise to resistant clones. Why do they choose to assay these chemotherapeutic drugs as matter of fact that the chemotherapy in general yields poor long-term results in ATL.

Overall the paper is interesting but the data are presented in a confused style not suitable for a rapid and complete comprehension of the importance of the topic. There is an excess of data for a full paper, with too many figures and supplementary data for the sake of clarity. The in vitro, ex vivo and computational data are described without a logic sequences. It seems that each co- authors have organized their own part and afterward put together the data losing the common thread. Therefore I invite the authors to revise the manuscript looking more precisely to the object of the paper and to the take home message for clarity versus the scientific community.

Referee #2 (Remarks for Author):

This is a very convincing and comprehensive study. By combining metabolomics and proteomics, the authors identify FDXR upregulation as a striking and HTLV-1-associated feature, and they further dissect the underlying mechanism through a wide range of complementary approaches, including ChIP-seq, RNA-seq, and CRISPR-based knockdown experiments. The study goes beyond descriptive observation by linking this phenomenon to disease severity, prognosis, and potential therapeutic vulnerability. Overall, this work provides important new insights not only into ATL biology but also into its possible diagnosis and treatment.

The data are generally strong and supportive of the main conclusions. I have only a few comments that may help further strengthen the manuscript.

Major comment

1. It would be interesting to clarify whether the p63/p73-FDXR axis is occurring mainly within infected cells themselves, or whether part of the observed effect may reflect intercellular/network-level regulation.
The finding of FDXR upregulation by p63/p73 is convincing. However, one point that I found particularly interesting is whether this phenomenon is occurring primarily within HTLV-1-infected cells themselves, or whether it might also be influenced by the cellular environment or cell-to-cell network effects.

The expression data shown in the cell lines are informative (for example, Fig. 1B for FDXR, Fig. 2C for TAp63, Fig. 2E for TAp73, and Fig. S2A for HBZ), and primary-cell data are also presented in Fig. 4D. If this is mainly a cell-intrinsic phenomenon in infected cells, one might expect some degree of correlation between markers of infected cells, such as CADM1 or HBZ, and the expression levels of TAp63, TAp73, and FDXR. At present, however, some of the presented data do not appear fully consistent with a simple linear relationship, at least visually.

For this reason, it may be helpful if the authors could consider adding correlation analyses between CADM1/HBZ and TAp63/TAp73/FDXR expression, if such analyses are feasible with the existing data. A more explicit discussion of this point would also be valuable, particularly if the results suggest that FDXR upregulation is not solely explained by the infected-cell fraction itself. This would further refine the biological interpretation of the proposed model.

Minor comments
1. Figure 1F
Since the data suggest that FDXR upregulation may be observed not only in ATL but more broadly in HTLV-1-associated conditions such as HAM, it may be of interest to comment on whether FDXR expression correlates with proviral load. Even if such analysis is not possible in the present study, a brief discussion of this possibility may be helpful.

2. Figures S4F and S4G
In the lymphoma type, p63 expression appears relatively high, whereas p73 expression appears lower than in indolent ATL. This is an interesting pattern. It would be helpful if the authors could comment on the possible biological interpretation of this difference.

Overall, this is an excellent and highly informative study. The manuscript is already strong, and the additional analyses or discussion suggested above would mainly help to further refine the interpretation of the findings.

Please find below our point-by-point responses in red. Please also note that while preparing the source data, we spotted one data point that was swapped between the IRF4 KO_1 and IRF4 KO_2 bars in Figure 3E. This visualization error has now been corrected. The statistical analysis had already been performed on the correctly assigned points and is therefore unchanged.

***** Reviewer's comments *****

Referee #1 (Comments on Novelty/Model System for Author):

The article presents novely aspects but ii si not well organized therefore this explains my personal choices of Medium technical quality and Medium medical impact

We thank the Reviewer for acknowledging the novelty of the work. In the revised manuscript we have addressed the points raised to improve the readability and clarity of the manuscript as detailed in the replies below.

Referee #1 (Remarks for Author):

Gallucci and coll have undertaking a study to demonstrate that FDXR upregulation could be a prognostic and therapeutic marker in HTLV-1 positive ATL.

Major points:
Title: In the title the authors underline that ATL is HTLV-1 associated, but since the abbrevation ATL refers only to adult T cell leukemia caused by HTLV-1 I suggest to use simply ATL.

In line with this comment, we have updated the title to "*FDXR Upregulation by p63/p73 is a Prognostic and Therapeutic Marker of Adult T Cell Leukemia/Lymphoma*"

Results:
The experimental section shown in the paper demonstrates with different techniques that FDXR gene is expressed at higher level in HTLV-1 infected cells either chronically infected and/or established from HTLV-1 positive patient in comparison with uninfected cell lines. In addition a mechanism involving RF-4/p63/p73/FDXR pathway has been hypothesized.This is the most complete, organized and understandable part of the paper.

We appreciate the supportive comment on these experimental sections.

Conversely the section illustrating the data ex vivo or meta-data regarding prognostic expression of FDXR are rather confusing. What is the relationship between the meta-analysis and the what the authors define as "our cohort of blood donors in Brazil" The analyzed cohort from Brazil (51 patients) is unclear in the description and conclusions. The patient samples refer to either ATL and TSP patients only from Brazil?. While it is clear that FDXR expression is higher in PLHTLV positive individual in comparison with healthy donors, the difference in FDXR expression in ATL versus TSP in the Brazilian blood donors cohort is not described/commented although the TSP CD4 cells express FDXR. The authors sustain that the FDXR marker is prognostic and therapeutic in ATL but in the Brazilian cohort of 51 individuals the

difference between FDXR expression in ATL versus TSP is not significant. What the authors want to demonstrate by analyzing the cohort. From the RNA-seq analysis the authors conclude that FDXR expression levels in PLHTLV correlate with disease progression and worse ATL prognosis, but the number of ATL is only 7 out of 43 samples. Therefore the authors should be more punctual in describing and motivating their ex vivo and meta-analysis studies.

To improve the clarity of the metanalysis and ex vivo experiments we have:

1) Updated Supplementary Table 3 (now called Table EV3 in line with Journal guidelines) to include the assays that were performed using samples from each donor of our newly established Brazilian cohort.

2) Included direct data citations in the reference list for each of the datasets that was re-used from public databases (these citations appear in the results as "Data ref" in line with Journal guidelines).

3) Included a direct comment on the ATL vs HAM/TSP comparison in the first paragraph of the Discussion. Specifically, we now write: *Our data further show that FDXR levels are similarly elevated in HAM/TSP and aggressive ATL. Although this supports a broader association between FDXR upregulation and HTLV-1 disease severity, the absence of matched FDXR expression and clinical outcome data currently precludes assessment of the prognostic potential of FDXR in HAM/TSP.*

In addition, we have clarified throughout the Results section the purpose of the different cohorts. In particular:

1) The metanalysis cohorts (Figure 1F) provided access to sequencing data from larger numbers of healthy controls and to HAM/TSP patients that were lacking the in the Kataoka et al. cohort.

2) The Kataoka et al. cohort (*e.g.*, Figure 1G-I) provided matched RNA-Seq and ATL-associated mortality data, allowing to formally analyse the prognostic value of FDXR in ATL. This prognostic analysis was conducted on 51 ATL patients. This is mentioned in the "overall cohort" number in Figure 1I and in the revised text in the results where we now write "*We next assessed the prognostic significance of FDXR expression levels on ATL patient survival by integrating RNA-Seq data (Data ref: Kataoka et al., 2015a) (Fig. 1G) with clinical outcome data available for 51 patients from the same cohort*".

3) Our newly established Brazilian cohort provided live cell samples that could be used for orthogonal validation of expression data (through qPCR) and direct assessment of drug efficacy data in primary cells, through Drug-Seq and immunofluorescence.

In particular we now write in the results: "*For further orthogonal validation, we assessed FDXR expression by qPCR in CD4 T cells obtained from an independent, newly recruited Brazilian cohort of PLHTLV and healthy donors (Table EV3, Figure EV2A-C)*"

FDXR transcription is enriched within the proliferating cell cluster of ATL. The authors do not precisely describe the origin of ATL cohort /NK clusters that they have analyzed.Are the same cohort of Brazilian donors or the data derive from meta-analysis?

These are entirely different donors. Specifically, they are derived from the cohort initially described in Tan et al. JCI 2021 doi: 10.1172/JCI150472. This dataset was selected in addition to the others outlined in the previous reply, because it offers the possibility to analyse single cell-expression of FDXR in ATL patients.

To improve clarity, we have now included a direct citation of this dataset in the reference list which is also referenced in the results paragraph and in the caption of the figure describing this analysis.

FDXR expression levels predict sensitivity to selected chemotherapeutics in vitro and ex vivo
In this case the prediction is based on profiles from the Cancer Therapeutics Response Portal (CTRP) Similarly the authors include in parenthesis without further description of the ATL cells that have been treated with bleomycin and fluorouracil in vitro. Is this the same Brazilian cohort ?
In general there is a remarkable confusion in combining the description of the results obtained in vitro, ex vivo, and through meta-analysis.

The ex vivo experiments were conducted on cells isolated from donors in our newly recruited Brazilian cohort. We have amended the text in this section of the results to further clarify this.

Specifically, the results now read: "*We next evaluated the effects of fluorouracil and bleomycin in primary ATL cells, by performing Drug-Seq on CD4 T cells isolated from five ATL donors (one acute, three chronic, and one smoldering) and three healthy donors all from our newly recruited cohort in Brazil (**Table EV3, Figure 5F**)*"

As mentioned above, we have also updated Table EV3 to specifically indicate which donors were used for each assay described in this section (Drug-Seq and immunofluorescence).

Discussion

The authors should comment why apparently the expression of FDXR is unrelated to the expression especially of regulatory genes in HTLV-1 infected cells. If the FDXR expression could be prognostic and therapeutic it should be influenced by the expression of regulatory genes as matter of fact that expression of TAX/HBZ change in vivo during the progression of the diseases. In addition although apparently bleomycin and fluorouracil have not been included in the therapy of ATL, the authors deserve to consider that the use of these drugs can give rise to resistant clones. Why do they choose to assay these chemotherapeutic drugs as matter of fact that the chemotherapy in general yields poor long-term results in ATL.

IRF4 is known to be upregulated through both HBZ and Tax signalling, and virtually all cell models and patient-derived cells express at least one of these two viral genes. Therefore, the observation that IRF4 is upregulated in a substantial subset of individuals with HTLV-1-associated disease, as well as in most HTLV-1-infected cell lines, may explain why FDXR levels tend to reflect overall disease severity rather than the expression of individual viral genes.

To clarify this in accordance with this comment, we have expanded the section in the Discussion about the upstream role of IRF4/BATF3 in regulating FDXR.

The updated discussion section now reads: *The potential link between IRF4 activation and FDXR upregulation through p63 and p73 would explain our observation that FDXR is upregulated in both ATL and HAM/TSP patients, which typically exhibit high expression of HBZ and Tax, respectively (Bangham, 2023; Bangham et al., 2015). IRF4 is also a known mutational hotspot in ATL (Kataoka et al., 2015b), which can lead to persistent activation of IRF4 independently of ongoing viral gene expression. This is in line with our observation that, at a single-cell level in ATL patients, FDXR expression significantly correlated with CADM1, but not HBZ expression.*

Regarding the choice of chemotherapeutic drugs, these agents were selected for two main reasons. First, the availability of FDXR as a marker of susceptibility to fluorouracil or bleomycin could enable pre-screening of individuals who are more likely to respond to these treatments. Second, their broad availability and ease of access, as off-patent drugs included in the WHO Essential Medicines List, could support their use in resource-limited settings, where HTLV-1 prevalence is often higher.

The Reviewer is correct that the risk of chemoresistance would remain even if bleomycin and fluorouracil should prove effective in ATL patients. We have added the sentence reported below to the Discussion acknowledging this limitation:

"*Although these compounds could represent a novel addition to ATL chemotherapy, resistance mechanisms to both fluorouracil and bleomycin have been described in various cancers (de Haas et al, 2008; Wang et al, 2004), and their likelihood in ATL remains currently unknown."*

Overall the paper is interesting but the data are presented in a confused style not suitable for a rapid and complete comprehension of the importance of the topic. There is an excess of data for a full paper, with too many figures and supplementary data for the sake of clarity. The in vitro, ex vivo and computational data are described without a logic sequences. It seems that each co- authors have organized their own part and afterward put together the data losing the common thread. Therefore I invite the authors to revise the manuscript looking more precisely to the object of the paper and to the take home message for clarity versus the scientific community.

In addition to the textual revisions and the updated Table EV3, which we hope have improved the clarity of the manuscript, we have sought to balance this comment with the new analyses recommended below by Reviewer n2. To this purpose we have moved five figures to the Appendix.

The other figures were preserved as Main or Extended View Figures based on editorial policies of the Journal (which encourage maximum data visibility) and after consultation with the Editor for this specific manuscript.
Conversely, the information now moved to the Appendix will be made available to readers as a separate supplementary file rather than being incorporated into the main manuscript. This should help address the concern about potential information overload while preserving technical validation details that may be helpful to specific readers interested in reproducing our findings.

Referee #2 (Remarks for Author):

This is a very convincing and comprehensive study. By combining metabolomics and proteomics, the authors identify FDXR upregulation as a striking and HTLV-1-associated feature, and they further dissect the underlying mechanism through a wide range of complementary approaches, including ChIP-seq, RNA-seq, and CRISPR-based knockdown experiments. The study goes beyond descriptive observation by linking this phenomenon to disease severity, prognosis, and potential therapeutic vulnerability. Overall, this work provides important new insights not only into ATL biology but also into its possible diagnosis and treatment.

The data are generally strong and supportive of the main conclusions. I have only a few comments that may help further strengthen the manuscript.

We thank the Reviewer for their very supportive comments and for highlighting the importance of the work.

Major comment

1. It would be interesting to clarify whether the p63/p73-FDXR axis is occurring mainly within infected cells themselves, or whether part of the observed effect may reflect intercellular/network-level regulation.
The finding of FDXR upregulation by p63/p73 is convincing. However, one point that I found particularly interesting is whether this phenomenon is occurring primarily within HTLV-1-infected cells themselves, or whether it might also be influenced by the cellular environment or cell-to-cell network effects.

The expression data shown in the cell lines are informative (for example, Fig. 1B for FDXR, Fig. 2C for TAp63, Fig. 2E for TAp73, and Fig. S2A for HBZ), and primary-cell data are also presented in Fig. 4D. If this is mainly a cell-intrinsic phenomenon in infected cells, one might expect some degree of correlation between markers of infected cells, such as CADM1 or HBZ, and the expression levels of TAp63, TAp73, and FDXR. At present, however, some of the presented data do not appear fully consistent with a simple linear relationship, at least visually.

For this reason, it may be helpful if the authors could consider adding correlation analyses between CADM1/HBZ and TAp63/TAp73/FDXR expression, if such analyses are feasible with the existing data. A more explicit discussion of this point would also be valuable, particularly if the results suggest that FDXR upregulation is

not solely explained by the infected-cell fraction itself. This would further refine the biological interpretation of the proposed model.

To address this question, we used the scRNA-seq dataset (Tan et al. JCI 2021) from ATL patients (showed in Figure 4). We found that, at a single-cell level, the expression of p63, p73 and FDXR displayed a modest but significant correlation with CADM1 expression. TP73 expression also correlated with the expression of HBZ. The data are reported in a new table (Table EV4, copied here for convenience) and accompanied by the following paragraph in the results: *"…expression of FDXR and its upstream regulators, TP63 and TP73, also displayed a modest but significant correlation with CADM1 expression (Table EV4). At a single cell level, TP73 additionally correlated with HBZ expression (Table EV4) consistent with previous findings (Toyoda et al., 2023). These analyses support our bulk RNA expression results (Fig. 1F,G, Fig. 2G,H) and suggest that, in addition to its enrichment in proliferating cells, FDXR and its upstream transcription factors are preferentially expressed in HTLV-1 infected cells, although not necessarily in cells with active viral expression."*

Table EV4. Single-cell correlations of HTLV-1/ATL markers with FDXR and its upstream regulators

gene 1	gene 2	Spearman correlation	P-value
FDXR	CADM1	0.029	< 0.001
TP73	CADM1	0.035	< 0.001
TP63	CADM1	0.054	< 0.001
FDXR	HBZ-HTLV	0.008	ns (0.493)
TP73	HBZ-HTLV	0.05	< 0.001
TP63	HBZ-HTLV	-0.01	ns (0.064)

Table EV4. Single-cell correlations of HTLV-1/ATL markers with FDXR and its upstream regulators. sc-RNA-Seq data from ATL patients were retrieved from (Tan et al., 2021a). A total of ten T/NK cell clusters (C0-C9) were identified across the dataset (91,436 cells total). The spearman's rank correlation coefficient was calculated between gene pairs in ATL samples across clusters (C0–C9). Statistical significance is calculated using an empirical depth-stratified permutation test.

Minor comments

1. Figure 1F

Since the data suggest that FDXR upregulation may be observed not only in ATL but more broadly in HTLV-1-associated conditions such as HAM, it may be of interest to comment on whether FDXR expression correlates with proviral load. Even if such analysis is not possible in the present study, a brief discussion of this possibility may be helpful.

We performed the correlation analysis suggested by the Reviewer using patient-matched RNA-seq and proviral load data from the same ATL cohort used for the prognostic analysis (Appendix Figure S2, reported below for convenience). The analysis showed a moderate but statistically significant positive correlation between FDXR expression and PVL in ATL patients (Spearman's $\rho = 0.355$, $p = 0.00755$). The number of matched FDXR-PVL data for HAM/TSP patients is unfortunately not sufficient to perform the same analysis in this group.

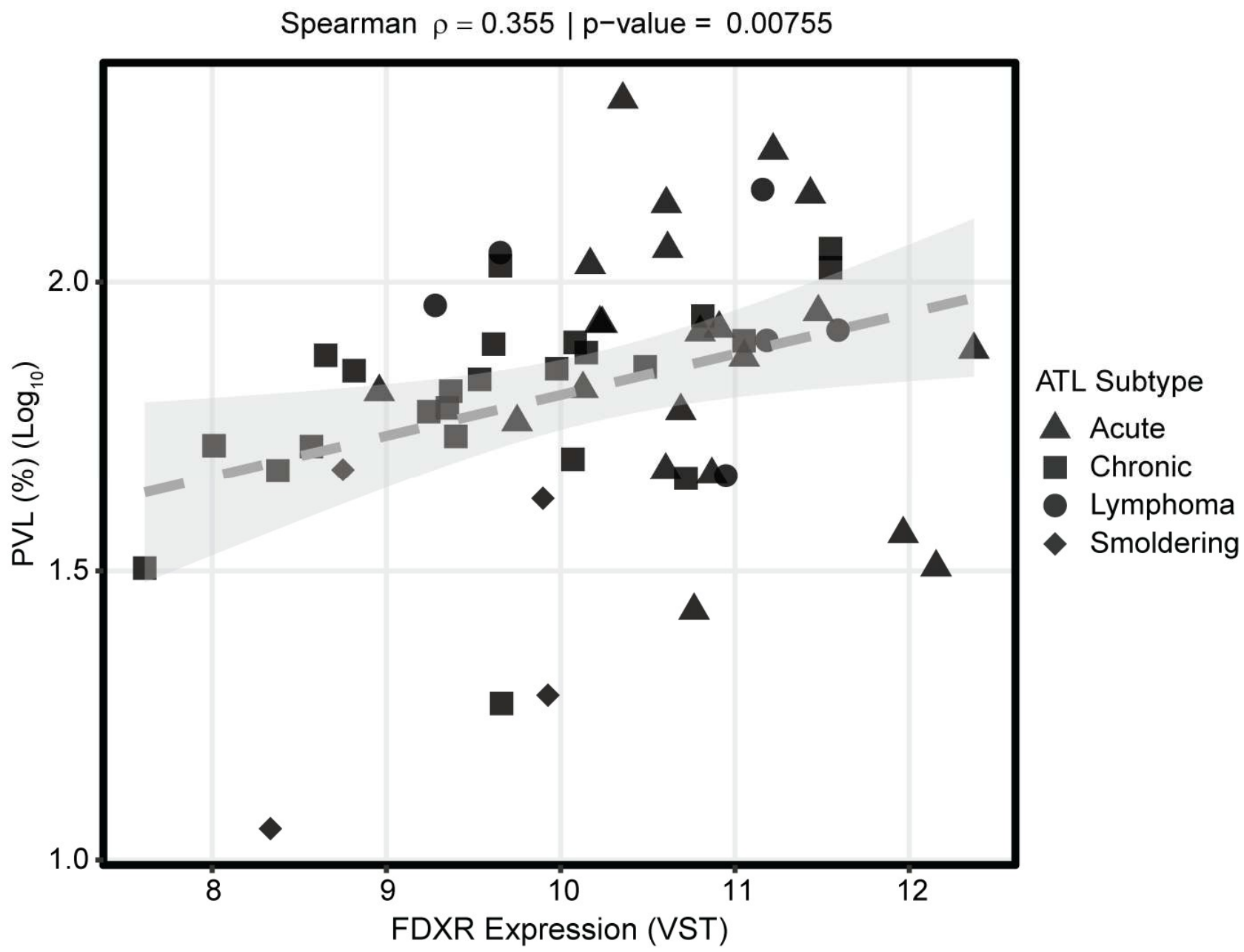

Appendix Figure S2. Related to Figure 1. Correlation between FDXR expression and proviral load in ATL patients. RNA-Seq data and HTLV-1 proviral load measurements in ATL patients were derived from (Kataoka et al., 2015a, Kataoka *et al.*, 2015b). Each point represents an individual patient, plotting variance-stabilized FDXR expression (x-axis) against Log10 proviral load (y-axis). Point shapes indicate ATL subtypes while the dashed line shows the linear regression fit and 95% confidence interval (grey shading). The correlation was calculated using the Spearman method.

We agree with this Reviewer (and with a similar comment of Reviewer n1) that the prognostic potential of FDXR upregulation could hold true for HAM/TSP as well. We have therefore added the following sentence to the Discussion acknowledging this possibility and noting that it could be tested in future once a HAM/TSP dataset with matched prognostic data should become available.

"Our data further show that FDXR levels are similarly elevated in HAM/TSP and aggressive ATL. Although this supports a broader association between FDXR upregulation and HTLV-1 disease severity, the absence of matched FDXR expression and clinical outcome data currently precludes assessment of the prognostic potential of FDXR in HAM/TSP."

2. Figures S4F and S4G
In the lymphoma type, p63 expression appears relatively high, whereas p73 expression appears lower than in indolent ATL. This is an interesting pattern. It would be helpful if the authors could comment on the possible biological interpretation of this difference.

We agree with the Reviewer that the pattern of p63 and p73 expression in lymphoma patients is intriguing and may suggest subtype-specific reliance on one or the other of these two p53 family members. Interestingly, this possibility is also suggested by our CRISPR-Cas9 experiments in ATL/HTLV-1 cell lines (Figure 2 K,N), where FDXR expression seems to rely more on either p63 or p73 depending on the cell model.

However, because lymphoma ATL is very rare and was not present in our validation cohort (in which we performed qPCR and Drug-seq experiments) this hypothesis remains speculative at present.

Overall, this is an excellent and highly informative study. The manuscript is already strong, and the additional analyses or discussion suggested above would mainly help to further refine the interpretation of the findings.

Many thanks!

7th Sep 2026

Dear Dr. Shytaj,

Thank you for submitting your revised manuscript, and please accept my apologies for the delay in getting back to you, due to the annual leave of both referees and members of the editorial team. I have now received the reports from the initial referees, and as you will see below, they are satisfied with the revisions. I will therefore be able to accept your manuscript once the following concerns are addressed:

1/ Manuscript text:
- Please remove the red font text and only indicate in track changes mode any new modification to the text.
- Please resolve the following name discrepancy: Alex Ap. Rosini Silva (manuscript) vs Alex Rosini Da Silva (submission system). We also received a bounced email alert for Rubens de Assis Santos Sebastião (rubens.sebastiao@online.uscs.edu.br), please provide the author's current email address.
- Remove the legends for the EV tables and for the appendx figures from the manuscript text.
- Methods:
o Clinical samples: Please provide a full statement confirming that the studies involving both human cohorts conformed to the principles set out in the WMA Declaration of Helsinki and the Department of Health and Human Services Belmont Report. Please also ensure that informed consent is stated for both cohorts.
o Cell lines: Please provide a statement on cell-line authentication.
o Experimental design and statistical reporting: Please provide information on sample-size determination, inclusion and exclusion criteria, randomization and blinding, as applicable to the different experiments.
- Data Availability Statement: Please remove the statement "All other data supporting this study are available in the article and in the Appendix or from the corresponding author upon reasonable request." Please also note that the deposited datasets must be made publicly accessible before acceptance of the manuscript, and provide URLs for both.
- Author contributions: Remove from the manuscript, and provide CRediT (Contributor Role Taxonomy) terms in the submission system. These replace a narrative author contribution section in the manuscript.

2/ Figures:
- You are welcome to make (some of) your Appendix figures into EV figures, that are collapsible/expandable online.
- Please provide less cropped images of the western blots.
- Please rename Tables EV1 and EV2 Dataset EV1 and Dataset EV2 and adjust the numbering of Tables EV3 - 5 accordingly (also in the reagents and tools table). Please add the legends of Tables EV1 - EV3 to the first page, no need to have it in a separate worksheet/tab.
- Please address the queries from our data editors in the figure legends:
1. Please note that figures 1-EV4 are not provided in the sequential manner. This needs to be rectified.
2. Please note that the exact p values are not provided in the legends of figures 1B, E, F, G; 2C, E, G, H, K, N; 3E, 5E, I; EV3 C, F, G
3. Please indicate the statistical test used for data analysis in the legends of figures 1A, EV1 B, D
4. Please note that information related to n is missing in the legend of figure 1H
5. Please note that the error bars are not defined in the legend of figure 1H.

3/ Author checklist:
- please fill in the section "Experimental study design and statistics", all subsections.
- please fill in the section "Data Availability", subsection "Human clinical and genomic datasets"

4/ Thank you for providing the Paper Explained. Please include it in the main manuscript text, after the abstract.

5/ Synopsis:
I introduced minor edits to your synopsis to match our style and format, please let me know if you agree or amend as you see fit:

Human T-cell lymphotropic virus 1 can cause adult T-cell leukemia/lymphoma (ATL), which is prone to chemoresistance and relapse. FDXR is identified as a biomarker of ATL progression and chemosensitivity.
- FDXR upregulation in patient-derived cells mirrors the severity of HTLV-1-associated disease.
- Elevated FDXR levels are linked to worse ATL prognosis.
- FDXR upregulation in HTLV-1+/ATL cells is driven by a pathway converging on two p53 family members, p63 and p73.
- Higher basal or drug-induced FDXR expression is associated with increased sensitivity of ATL cells to fluorouracil and bleomycin.

Thank you for providing a nice visual abstract. Please resize it to 550 px wide x 300 px high and increase the font to make sure the text remains legible.

6/ As part of the EMBO Publications transparent editorial process initiative (see our Editorial at http://embomolmed.embopress.org/content/2/9/329), EMBO Molecular Medicine will publish online a Review Process File (RPF) to accompany accepted manuscripts.
This file will be published in conjunction with your paper and will include the anonymous referee reports, your point-by-point response and all pertinent correspondence relating to the manuscript. Let us know whether you agree with the publication of the RPF and as here, if you want to remove or not any figures from it prior to publication.
Please note that the Authors checklist will be published at the end of the RPF.

I look forward to receiving your revised manuscript.

Yours sincerely,

Lise Roth

Lise Roth, PhD
Senior Editor
EMBO Molecular Medicine

***** Reviewer's comments *****

Referee #1 (Remarks for Author):

The manuscript is attractive and deserves future implementation and development to clarify prognostic factors in HTLV-1 infection.

Referee #2 (Remarks for Author):

I appreciate the authors' careful responses to my comments and the revisions made to the manuscript. My previous concerns have been adequately addressed, and the manuscript has been improved accordingly. I am satisfied with the revised version and am pleased to recommend its acceptance for publication.

Dear Dr Roth,

We are very glad that the Reviewers found our revised manuscript suitable for publication. We have now updated the text and associated files to address all Editorial comments, as detailed in the replies below.

1/Manuscript text:

- Please remove the red font text and only indicate in track changes mode any new modification to the text. Done

- Please resolve the following name discrepancy: Alex Ap. Rosini Silva (manuscript) vs Alex Rosini Da Silva (submission system). We also received a bounced email alert for Rubens de Assis Santos Sebastião (rubens.sebastiao@online.uscs.edu.br), please provide the author's current email address. The correct spelling and up to date e-mail have been provided in the submission system.

- Remove the legends for the EV tables and for the appendix figures from the manuscript text. Done

Methods:

o Clinical samples: Please provide a full statement confirming that the studies involving both human cohorts conformed to the principles set out in the WMA Declaration of Helsinki and the Department of Health and Human Services Belmont Report. Please also ensure that informed consent is stated for both cohorts. Done

o Cell lines: Please provide a statement on cell-line authentication. Done

o Experimental design and statistical reporting: Please provide information on sample-size determination, inclusion and exclusion criteria, randomization and blinding, as applicable to the different experiments. Done

- Data Availability Statement: Please remove the statement "All other data supporting this study are available in the article and in the Appendix or from the corresponding author upon reasonable request." Please also note that the deposited datasets must be made publicly accessible before acceptance of the manuscript, and provide URLs for both. Done

- Author contributions: Remove from the manuscript, and provide CRediT (Contributor Role Taxonomy) terms in the submission system. These replace a narrative author contribution section in the manuscript. Done

2/Figures:

- You are welcome to make (some of) your Appendix figures into EV figures, that are collapsible/expandable online. Thank you for the opportunity, we rechecked all figures and the current balance between the main, EV, and Appendix figures seems to work well.

- Please provide less cropped images of the western blots. Less cropped western blots

were provided for Panels 1C, 2D, 2F, i.e., the western blots where the previous figure borders were narrower.

- Please rename Tables EV1 and EV2 Dataset EV1 and Dataset EV2 and adjust the numbering of Tables EV3 - 5 accordingly (also in the reagents and tools table). Done

- Please add the legends of Tables EV1 - EV3 to the first page, no need to have it in a separate worksheet/tab. Done

- Please address the queries from our data editors in the figure legends:
1. Please note that figures 1-EV4 are not provided in the sequential manner. This needs to be rectified. We went through the manuscript and could not find mismatches in the orders in which the figures are called. Therefore, we did not make changes on this.

2. Please note that the exact p values are not provided in the legends of figures 1B, E, F, G; 2C, E, G, H, K, N; 3E, 5E, I; EV3 C, F, G. Done. Please also note that the original statistical tests in Figures 1E, 2C, 2E and EV3C provided approximate p values. To provide exact p values as requested we repeated the test performing the whole permutation set in Figure 1E and updating the setting to "exact" for 2C, E, EV3C. Therefore, the mention of "100,000 permutations" has been removed for Figure 1E and the asterisks have been updated for Figures 2E (for this panel the exact p value calculation led to a slightly higher significance level corresponding to 3 asterisks instead of 2 asterisks).

3. Please indicate the statistical test used for data analysis in the legends of figures 1A, EV1 B, D. Done.

4. Please note that information related to n is missing in the legend of figure 1H Done

5. Please note that the error bars are not defined in the legend of figure 1H. Done

3/ Author checklist:
- please fill in the section "Experimental study design and statistics", all subsections. Done
- please fill in the section "Data Availability", subsection "Human clinical and genomic datasets" Done

4/ Thank you for providing the Paper Explained. Please include it in the main manuscript text, after the abstract. Done

5/ Synopsis:
I introduced minor edits to your synopsis to match our style and format, please let me know if you agree or amend as you see fit: Thank you, we agree with this version of the synopsis

Thank you for providing a nice visual abstract. Please resize it to 550 px wide x 300 px high and increase the font to make sure the text remains legible. Done

6/ As part of the EMBO Publications transparent editorial process initiative (see our Editorial at http://embomolmed.embopress.org/content/2/9/329), EMBO Molecular Medicine will publish online a Review Process File (RPF) to accompany accepted manuscripts.
This file will be published in conjunction with your paper and will include the anonymous referee reports, your point-by-point response and all pertinent correspondence relating to the manuscript. Let us know whether you agree with the publication of the RPF and as here, if you want to remove or not any figures from it prior to publication.
Please note that the Authors checklist will be published at the end of the RPF. We agree with publication of the RPF.

22nd Sep 2026

Dear Dr. Shytaj,

Thank you for submitting your revised files. I am pleased to inform you that your manuscript has now been accepted for publication.

Please note that the visual abstract provided does not meet the journal's size requirements. Could you please send us a new version measuring exactly 550 px wide × 300 px high at your earliest convenience?

Once we receive the revised visual abstract, your article will be sent to our publisher for inclusion in the next available issue of EMBO Molecular Medicine.

Your manuscript will be processed for publication by EMBO Press. It will be copy edited and you will receive page proofs prior to publication. Please note that you will be contacted by Springer Nature Author Services to complete licensing and payment information.

You may qualify for financial assistance for your publication charges - either via a Springer Nature fully open access agreement or an EMBO initiative. Check your eligibility: *Link Unavailable*

Should you be planning a Press Release on your article, please notify the production team via this form in order to coordinate publication and release dates: *Link Unavailable*

If you have any questions, please do not hesitate to contact the Editorial Office. Thank you for your contribution to EMBO Molecular Medicine.

Yours sincerely,

Lise Roth

Lise Roth, Ph.D
Senior Editor
EMBO Molecular Medicine

>>> Please note that it is EMBO Molecular Medicine policy for the transcript of the editorial process (containing referee reports and your response letter) to be published as an online supplement to each paper. If you do NOT want this, you will need to inform the Editorial Office via email immediately. More information is available here: https://link.springer.com/partners/embo-press/editorial-policies#Peer%20review

EMBO Press Author Checklist

Corresponding Author Name: Iart Luca Shytaj	
Journal Submitted to: EMBO Molecular Medicine	
Manuscript Number: EMM-2026-23692	

USEFUL LINKS FOR COMPLETING THIS FORM
The EMBO Journal - Author Guidelines
EMBO Reports - Author Guidelines
Molecular Systems Biology - Author Guidelines
EMBO Molecular Medicine - Author Guidelines

Reporting Checklist for Life Science Articles (updated January
This checklist is adapted from Materials Design Analysis Reporting (MDAR) Checklist for Authors. MDAR establishes a minimum set of requirements in transparent reporting in the life sciences (see Statement of Task: 10.31222/osf.io/9sm4x). Please follow the journal's guidelines in preparing your
Please note that a copy of this checklist will be published alongside your article.

Abridged guidelines for figures
1. Data
The data shown in figures should satisfy the following conditions:
➞ the data were obtained and processed according to the field's best practice and are presented to reflect the results of the experiments in an accurate and unbiased manner.
➞ ideally, figure panels should include only measurements that are directly comparable to each other and obtained with the same assay.
➞ plots include clearly labeled error bars for independent experiments and sample sizes. Unless justified, error bars should not be shown for technical
➞ if n<5, the individual data points from each experiment should be plotted. Any statistical test employed should be justified.
➞ Source Data should be included to report the data underlying figures according to the guidelines set out in the authorship guidelines on Data

2. Captions
Each figure caption should contain the following information, for each panel where they are relevant:
➞ a specification of the experimental system investigated (eg cell line, species name).
➞ the assay(s) and method(s) used to carry out the reported observations and measurements.
➞ an explicit mention of the biological and chemical entity(ies) that are being measured.
➞ an explicit mention of the biological and chemical entity(ies) that are altered/varied/perturbed in a controlled manner.
➞ the exact sample size (n) for each experimental group/condition, given as a number, not a range;
➞ a description of the sample collection allowing the reader to understand whether the samples represent technical or biological replicates (including how many animals, litters, cultures, etc.).
➞ a statement of how many times the experiment shown was independently replicated in the laboratory.
➞ definitions of statistical methods and measures:
- common tests, such as t-test (please specify whether paired vs. unpaired), simple χ2 tests, Wilcoxon and Mann-Whitney tests, can be unambiguously identified by name only, but more complex techniques should be described in the methods section;
- are tests one-sided or two-sided?
- are there adjustments for multiple comparisons?
- exact statistical test results, e.g., P values = x but not P values < x;
- definition of 'center values' as median or average;
- definition of error bars as s.d. or s.e.m.

Please complete ALL of the questions below.
Select "Not Applicable" only when the requested information is not relevant for your study.

Materials

Newly Created Materials	Information included in the manuscript?	In which section is the information available? (Reagents and Tools Table, Materials and Methods, Figures, Data Availability Section)
New materials and reagents need to be available; do any restrictions apply?	Yes	Materials and Methods and Reagents and Tools Table

Antibodies	Information included in the manuscript?	In which section is the information available? (Reagents and Tools Table, Materials and Methods, Figures, Data Availability Section)
For **antibodies** provide the following information:<br>- Commercial antibodies: RRID (if possible) or supplier name, catalogue number and or/clone number<br>- Non-commercial: RRID or citation	Yes	Materials and Methods and Reagents and Tools Table

DNA and RNA sequences	Information included in the manuscript?	In which section is the information available? (Reagents and Tools Table, Materials and Methods, Figures, Data Availability Section)
**Short novel DNA or RNA including primers, probes**: provide the sequences.	Yes	Table EV5

Cell materials	Information included in the manuscript?	In which section is the information available? (Reagents and Tools Table, Materials and Methods, Figures, Data Availability Section)
**Cell lines:** Provide species information, strain. Provide accession number in repository **OR** supplier name, catalog number, clone number, and/**OR** RRID.	Yes	Materials and Methods and Reagents and Tools Table
**Primary cultures:** Provide species, strain, sex of origin, genetic modification status.	Yes	Materials and Methods and Reagents and Tools Table
Report if the cell lines were recently **authenticated** (e.g., by STR profiling) and tested for mycoplasma contamination.	Yes	Materials and Methods

Experimental animals	Information included in the manuscript?	In which section is the information available? (Reagents and Tools Table, Materials and Methods, Figures, Data Availability Section)
**Laboratory animals or Model organisms:** Provide species, strain, sex, age, genetic modification status. Provide accession number in repository **OR** supplier name, catalog number, clone number, **OR** RRID.	Not Applicable	
**Animal observed in or captured from the field:** Provide species, sex, and age where possible.	Not Applicable	
Please detail **housing and husbandry conditions**.	Not Applicable	

Plants and microbes	Information included in the manuscript?	In which section is the information available? (Reagents and Tools Table, Materials and Methods, Figures, Data Availability Section)
**Plants:** provide species and strain, ecotype and cultivar where relevant, unique accession number if available, and source (including location for collected wild specimens).	Not Applicable	
**Microbes:** provide species and strain, unique accession number if available, and source.	Not Applicable	

Human research participants	Information included in the manuscript?	In which section is the information available? (Reagents and Tools Table, Materials and Methods, Figures, Data Availability Section)
If collected and within the bounds of privacy constraints report on age, sex and gender or ethnicity for all study participants.	Yes	Table EV3

Core facilities	Information included in the manuscript?	In which section is the information available? (Reagents and Tools Table, Materials and Methods, Figures, Data Availability Section)
If your work benefited from core facilities, was their service mentioned in the acknowledgments section?	Yes	Acknowledgments

Design

Study protocol	Information included in the manuscript?	In which section is the information available? (Reagents and Tools Table, Materials and Methods, Figures, Data Availability Section)
If study protocol has been **pre-registered, provide DOI in the manuscript**. For clinical trials, provide the trial registration number **OR** cite DOI.	Not Applicable	
Report the **clinical trial registration number** (at ClinicalTrials.gov or equivalent), where applicable.	Not Applicable	

Laboratory protocol	Information included in the manuscript?	In which section is the information available? (Reagents and Tools Table, Materials and Methods, Figures, Data Availability Section)
Provide DOI OR other citation details if **external detailed step-by-step protocols** are available.	Not Applicable	

Experimental study design and statistics	Information included in the manuscript?	In which section is the information available? (Reagents and Tools Table, Materials and Methods, Figures, Data Availability Section)
Include a statement about **sample size** estimate even if no statistical methods were used.	Yes	Materials and Methods
Were any steps taken to minimize the effects of subjective bias when allocating animals/samples to treatment (e.g. **randomization procedure**)? If yes, have they been described?	Yes	Materials and Methods
Include a statement about **blinding** even if no blinding was done.	Yes	Materials and Methods
Describe **inclusion/exclusion criteria** if samples or animals were excluded from the analysis. Were the criteria pre-established?<br><br>If sample or data points were omitted from analysis, report if this was due to attrition or intentional exclusion and provide justification.	Yes	Materials and Methods
For every figure, are **statistical tests** justified as appropriate? Do the data meet the assumptions of the tests (e.g., normal distribution)? Describe any methods used to assess it. Is there an estimate of variation within each group of data? Is the variance similar between the groups that are being statistically compared?	Yes	Materials and Methods, Figure captions

Sample definition and in-laboratory replication	Information included in the manuscript?	In which section is the information available? (Reagents and Tools Table, Materials and Methods, Figures, Data Availability Section)
In the figure legends: state number of times the experiment was **replicated** in laboratory.	Yes	Figure captions
In the figure legends: define whether data describe **technical or biological replicates**.	Yes	Figure captions

Ethics

Ethics	Information included in the manuscript?	In which section is the information available? (Reagents and Tools Table, Materials and Methods, Figures, Data Availability Section)
Studies involving **human participants**: State details of **authority granting ethics approval** (IRB or equivalent committee(s), provide reference number for approval.	Yes	Materials and Methods
Studies involving **human participants**: Include a statement confirming that **informed consent** was obtained from all subjects and that the experiments conformed to the principles set out in the WMA Declaration of Helsinki and the Department of Health and Human Services Belmont Report.	Yes	Materials and Methods
Studies involving **human participants:** For publication of **patient photos**, include a statement confirming that consent to publish was obtained.	Not Applicable	
Studies involving experimental **animals**: State details of **authority granting ethics approval** (IRB or equivalent committee(s), provide reference number for approval. Include a statement of compliance with ethical regulations.	Not Applicable	
Studies involving **specimen and field samples:** State if relevant **permits** obtained, provide details of authority approving study; if none were required, explain why.	Not Applicable	

Dual Use Research of Concern (DURC)	Information included in the manuscript?	In which section is the information available? (Reagents and Tools Table, Materials and Methods, Figures, Data Availability Section)
Could your study fall under dual use research restrictions? Please check biosecurity documents and list of **select agents and toxins** (CDC): https://www.selectagents.gov/sat/list.htm	Not Applicable	
If you used a select agent, is the security level of the lab appropriate and reported in the manuscript?	Not Applicable	
If a study is subject to dual use research of concern regulations, is the name of the **authority granting approval and reference number** for the regulatory approval provided in the manuscript?	Not Applicable	

Reporting

The MDAR framework recommends adoption of discipline-specific guidelines, established and endorsed through community initiatives. Journals have their own policy about requiring specific guidelines and recommendations to complement MDAR.

Adherence to community standards	Information included in the manuscript?	In which section is the information available? (Reagents and Tools Table, Materials and Methods, Figures, Data Availability Section)
State if relevant guidelines or checklists (e.g., **ICMJE, MIBBI, ARRIVE, PRISMA**) have been followed or provided.	Yes	Author contributions, Data Availability
For **tumor marker prognostic studies,** we recommend that you follow the **REMARK** reporting guidelines (see link list at top right). See author guidelines, under 'Reporting Guidelines'. Please confirm you have followed these guidelines.	Yes	Material and Methods, Results, Figure Captions
For **phase II and III randomized controlled trials**, please refer to the **CONSORT** flow diagram (see link list at top right) and submit the CONSORT checklist (see link list at top right) with your submission. See author guidelines, under 'Reporting Guidelines'. Please confirm you have submitted this list.	Not Applicable	

Data Availability

Data availability	Information included in the manuscript?	In which section is the information available? (Reagents and Tools Table, Materials and Methods, Figures, Data Availability Section)
Have **primary datasets** been deposited according to the journal's guidelines (see 'Data Deposition' section) and the respective accession numbers provided in the Data Availability Section?	Yes	Data Availability
Were **human clinical and genomic datasets** deposited in a public access-controlled repository in accordance to ethical obligations to the patients and to the applicable consent agreement?	Yes	Data Availability
Are **computational models** that are central and integral to a study available without restrictions in a machine-readable form? Were the relevant accession numbers or links provided?	Not Applicable	
If publicly available data were reused, provide the respective **data citations in the reference list**.	Yes	Materials and Methods, References
